# Coralmycin Derivatives with Potent Anti-Gram Negative Activity Produced by the Myxobacteria *Corallococcus coralloides* M23

**DOI:** 10.3390/molecules24071390

**Published:** 2019-04-09

**Authors:** Bo-Min Kim, Nguyen Van Minh, Ha-Young Choi, Won-Gon Kim

**Affiliations:** 1 Superbacteria Research Center, Korea Research Institute of Bioscience and Biotechnology, Yusong, Daejeon 305-806, Korea; bobo0816@kribb.re.kr (B.-M.K.); vanminh.nguyenvm@gmail.com (N.V.M.); chy9274@kribb.re.kr (H.-Y.C.); 2 Department of Bio-Molecular Science, KRIBB School of Bioscience, Korea University of Science and Technology (UST), Yusong, Daejeon 305-806, Korea; 3 College of Pharmacy, Chungnam National University, Daejeon 34134, Korea

**Keywords:** *Corallococcus coralloides*, coralmycin, antibacterial, Gram-negative, DNA gyrase

## Abstract

Seven new coralmycin derivatives, coralmycins C (**1**), D (**2**), E (**3**), F (**4**), G (**5**), H (**6**), and I (**7**), along with three known compounds, cystobactamids 891-2 (8), 905-2 (9), and 507 (10), were isolated from a large-scale culture of the myxobacteria *Corallococcus coralloides* M23. The structures of these compounds, including their relative stereochemistries, were elucidated by interpretation of their spectroscopic and CD data. The structure-activity relationships of their antibacterial and DNA gyrase inhibitory activities indicated that the *para*-nitrobenzoic acid unit is critical for the inhibition of DNA gyrase and bacterial growth, while the nitro moiety of the *para*-nitrobenzoic acid unit and the isopropyl chain at C-4 could be important for permeability into certain Gram-negative bacteria, including *Pseudomonas aeruginosa* and *Klebsiella pneumoniae*, and the β-methoxyasparagine moiety could affect cellular uptake into all tested bacteria. These results could facilitate the chemical optimization of coralmycins for the treatment of multidrug-resistant Gram-negative bacteria.

## 1. Introduction

Concerns about multidrug-resistant (MDR) bacteria have increased worldwide. In particular, infections caused by MDR Gram-negative bacteria have become an urgent problem because no therapeutic agents active against carbapenem-resistant Gram-negative bacteria are currently available [1,2]. The “ESKAPE” pathogens (i.e., *Enterococcus faecium*, *Staphylococcus aureus*, *Klebsiella pneumoniae*, *Acinetobacter baumannii*, *Pseudomonas aeruginosa*, and *Enterobacter* spp.), MDR bacteria capable of “escaping” the biocidal action of current antibiotics, include four MDR Gram-negative bacteria (*K. pneumoniae*, *A. baumannii*, *P. aeruginosa*, and *Enterobacter* sp.) [3]. Thus, there is an urgent need for new drugs to treat infections caused by MDR Gram-negative pathogens.

Recently, our group reported a new class of antibiotics, coralmycins A (**12**) and B (**13**), from cultures of the myxobacteria *Corallococcus coralloides* M23 [4]. Compound **12** shows potent antibacterial activity against clinically important Gram-negative pathogens as well as Gram-positive bacteria. Muller’s group reported cystobactamids 507 (**10**) and 919-2 (**11**) isolated from another myxobacteria, *Cystobacter* sp. [5]. Compound **12**, a hydroxylated derivative of **11**, has 10 times higher antibacterial activity than **11**. Compound **10** shows weak antibacterial activity. Compound **11** and its structurally similar albicidin were reported to inhibit DNA gyrase [5,6]. Very recently, Muller’s group reported the discovery of new cystobactamids and **12** from a repeated-batch fermentation of another myxobacteria *Myxococcus* sp. for the sufficient production of cystobactamid derivatives occurred at very low product titers [7]. One of the new cystobactamids, cystobactamid 861-2, exhibit 4–10 times higher antibacterial activity against Gram-negative pathogens than **12**.

**Figure 1:**
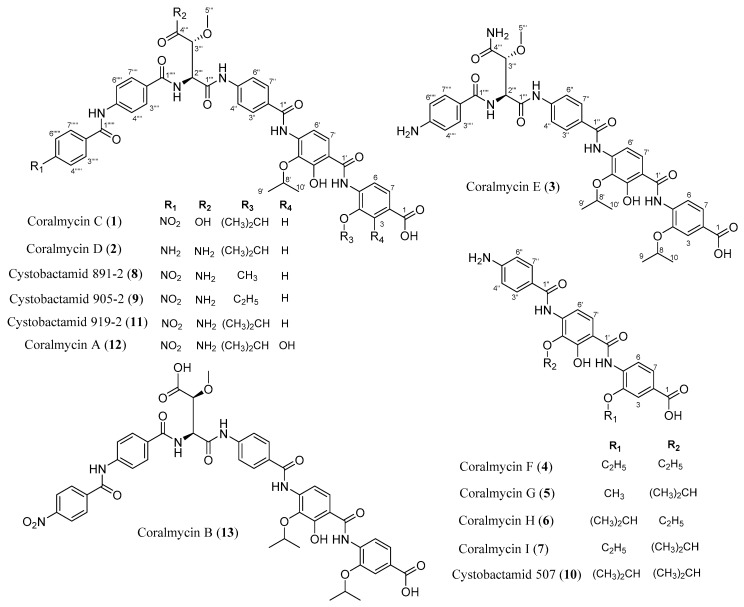
The structures of coralmycins C (**1**), D (**2**), E (**3**), F (**4**), G (**5**), H (**6**), and I (**7**), and related compounds.

Previously, compounds showing a UV absorption spectrum similar to those of the coralmycins were detected in minute amounts during isolation of **12** and **13**. From a large-scale culture of *Corallococcus coralloides* M23 for the production of sufficient amounts of the coralmycin derivatives, we isolated seven new compounds (coralmycins C (**1**), D (**2**), E (**3**), F (**4**), G (**5**), H (**6**), and I (**7**)) and three known compounds (cystobactamids 891-2 (**8**), 905-2 (**9**), and **10**) (Figure 1). Compound **1** was determined to be a new stereoisomer of **13** with the same configuration as **11**, **2** is a new derivative of **11** with an amino moiety instead of the nitro moiety present in **11**, and **3** is a new derivative of **11** that lacks the *para*-nitrobenzoic acid unit. Compounds **4**–**7** are new derivatives of **10** with variations on their two isopropyl chains. In this study, we report the fermentation, isolation, structural determination, and antibacterial and DNA gyrase inhibitory activities of **1**–**10**. 

## 2. Results and Discussion 

### 2.1. Structural Elucidation

The chemical structures of **8** and **9** were independently elucidated using HRESIMS and 1D and 2D NMR analyses (Figures S1–S8 and Table 1 and Table 2). Their β-methoxyaspargine moieties were determined to be anti-relative configuration based on their large ^3^*J*_HH_ coupling constant between H-2′′′ and H-3′′′. Additionally, the Cotton effects of **8** and **9** [[θ]^25^(nm)(MeOH): −703 (252), 508 (310)] and [[*θ*]^25^(nm)(MeOH): −746 (252), 488 (309), respectively], were almost the same as those of **11** [[*θ*]^25^(nm)(MeOH): −5344 (251), 3999 (305)] [4] in their circular dichroism (CD) spectra (Figure S9). Thus, **8** and **9** were identified as cystobactamids 891-2 and 905-2 [7], respectively. The complete assignments of the ^13^C-NMR data of **8** and **9** are reported for the first time in this study.

**Figure 2:**
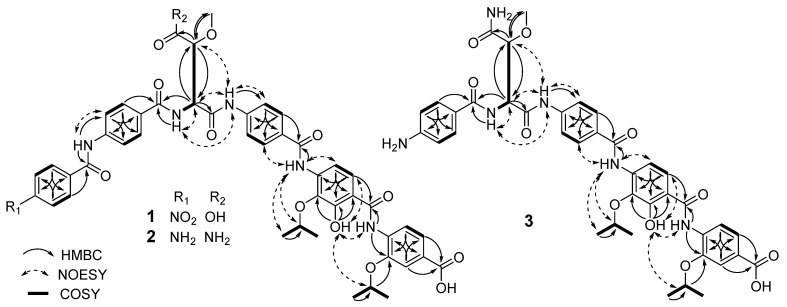
Key HMBC, COSY, and NOE correlations of **1**–**3**.

Compound **1** was not separated from **13** in SiO2 TLC but separated by ODS HPLC (Figure S10). Compound **1** showed the same molecular formula (C_46_H_44_N_6_O_15_) as that of **13** based on their HRESIMS data in combination with their ^1^H and ^13^C NMR data (Figures S11–S15 and Table 1 and Table 2). Various spectroscopic techniques, including 1D and 2D NMR experiments, were used for the structure elucidation of **1**. The ^1^H-NMR and HMQC spectra of **1** were very similar to those of **13**. The difference between the spectra of **1** and **13** is that the chemical shifts and coupling constants of H-2′′′ and H-3′′′ of the β-methoxyaspartic acid unit of **1** are more similar to those of **11** rather than **13** (Table 3), suggesting that **1** could be a stereoisomer of **13** at the β-methoxyaspartic acid moiety. The signals at δ_H_ 4.75 (1H, dt, *J =* 6.0, 12.1 Hz; δ_C_ 72.1), 4.31 (1H, dt, *J =* 6.1, 12.2 Hz; δ_C_ 76.1), 1.37 (6H, d, *J =* 6.0 Hz; δ_C_ 22.0), and 1.26 (6H, d, *J =* 6.1 Hz; δ_C_ 22.3) suggested the presence of two isopropyl groups. Additionally, the ^1^H-NMR, COSY, and HMQC spectra of **1** indicated the presence of two *para*-aminobenzoic acid units, a *para*-nitrobenzoic acid unit, a 4-amino-3-isopropoxybenzoic acid unit, a 4-amino-2-hydroxyl-3-isopropoxybenzoic acid unit, and a β-methoxyaspartic acid unit. The NH_2_ signal of the β-methoxyasparagine unit of **11** was not detected in the spectrum of **1** acquired in DMSO-*d*_6_ (Table 1), which, together with the molecular formula, suggested the presence of a β-methoxyaspartic acid fragment. The connectivities of these units were confirmed by the HMBC spectrum (Figure 2). Thus, the planar structure of **1** was elucidated to be the same as that of **13** [4]. 

The relative configuration at the β-methoxyaspartic acid unit of **1** was determined from ^3^*J*_HH_ and ^2^*J*_CH_ coupling constants and NOESY and NOE differential spectra (Table 2 and Figures S16–S18). The large coupling constant (8.1 Hz) between H-2′′′ and H-3′′′ indicated the anti-*c*onfiguration. The ^2^*J*_CH_ coupling constants were measured in DMSO-*d_6_* by a HECADE experiment (Figure S16). The large coupling constant (5.11 Hz) between H-2′′′ and C-3′′′ indicated that H-2′′′ and OMe are in a *gauche*. Together with these coupling constants, the NOEs between H-3′′′ and NH-6′′′, from NH-8′′ to H-3′′′ and OMe, and from H-2′′′ to OMe confirmed that **1** had the same relative configuration as **11** (Figure S19). An unexpected NOE from H-2′′′ to H-3′′′ suggested the presence of the other rotamer as a minor conformer as detected previously in **13** [4]. The Cotton effect [[*θ*]^25^(nm)(MeOH): −415 (250), 345 (304)] in the CD spectra of **1** was almost the same as that [[*θ*]^25^(nm)(MeOH): −5344 (251), 3999 (305)] of **11** [4] (Figure S9). These data clearly indicated that **1** had the same configuration (S*R*) as **11**. Thus, **1** was elucidated to be a stereoisomer of **13**.

The HRESIMS data of **2** gave a protonated molecule at *m/z* 890.3415 [M + H]^+^ (calcd. 890.3362 for C_46_H_48_N_7_O_12_), which in combination with ^1^H and ^13^C NMR spectra suggested a molecular formula of C_46_H_47_N_7_O_12_ (Figures S20–S24 and Table 1 and Table 2). The ^1^H NMR spectrum of **2** was similar to that of **11**. The difference was the appearance of the aromatic proton signals [δ_H_ 7.75 (2H, d, *J =* 8.8 Hz; δ_C_ 129.9) and 6.62 (2H, d, *J =* 8.6 Hz; δ_C_ 112.9)] for an 1,4-disubstituted phenyl moiety instead of signals (δ_H_ 8.39 and 8.21) for the *para*-nitrobenzoic acid unit in **11** (Table 1). In the HMBC spectrum (Figure 2), the proton at δ_H_ 6.62 showed long-range coupling to the carbons at δ_C_ 113.1 and 120.9, and the proton at δ_H_ 7.75 showed long-range coupling to the carbons at δ_C_ 165.9, 152.8, and 129.9. These ^13^C NMR data were consistent with those of *para*-aminobenzoic acid [8]. Together with the molecular formula, these spectroscopic data indicated the presence of a *para*-aminobenzoic acid unit instead of a *para*-nitrobenzoic acid unit. The connectivity of the remaining units was confirmed by the HMBC spectrum (Figure 2). Thus, the planar structure of **2** was identified as that of a new, C-5′′′′′ amino derivative of **11**. 

The relative configuration of the β-methoxyasparagine moiety of **2** was determined from ^3^*J*_HH_ and ^2^*J*_CH_ coupling constants and NOESY and NOE differential spectra (Table 1 and Figures S25–S27). The coupling constant between H-2′′′ and H-3′′′ was 8.0 Hz, indicating the *anti* configuration. Similar to **1**, the large ^2^*J*_CH_ coupling constant (5.49 Hz) between H-2′′′ and C-3′′′ indicated a *gauche* conformation of H-2′′′ and OMe. The NOEs between H-3′′′ and NH-6′′′, from NH-8′′ to H-3′′′ and OMe, and from H-2′′′ to OMe supported the same relative stereochemistry as **11**. The Cotton effect [[*θ*]^25^(nm)(MeOH): −1278 (256), 1301 (313)] of **2** was almost the same as that [[*θ*]^25^(nm)(MeOH): −5344 (251), 3999 (305)] of **11** in CD spectra (Figure S9). Thus, **2** was determined to be a new, C-5′′′′′ amino derivative of **11**.

The molecular formula of **3** was determined to be C_39_H_24_N_6_O_11_ based on its HRESIMS data in combination with its ^1^H and ^13^C NMR data (Figures S28–S30 and Table 1 and Table 2). The ^1^H NMR spectrum of **3** was similar to that of **2**. The difference between the spectra of **3** and **2** was that the overlapping aromatic proton signals [δ_H_ 7.84 (4H, brs)] and the amino signal [δ_H_ 10.02 (1H, s)] of the *para*-aminobenzoic acid unit in the spectrum of **2** were not observed in the spectrum of **3**. Additionally, detailed analysis of its ^1^H NMR, ^13^C NMR, COSY, and HMQC spectra suggested the presence of two *para*-aminobenzoic acid fragments, a 4-amino-3-isopropoxybenzoic acid fragment, a 4-amino-2-hydroxyl 3-isopropoxybenzoic acid fragment, and a β-methoxyasparagine fragment. These spectroscopic data suggested that **3** had one fewer *para*-aminobenzoic acid unit than were present in **2**. The aromatic proton at δ_H_ 7.58 (H-3′′′′ and H-7′′′′) of one *para-*aminobenzoic acid unit [δ_H_ 7.58 (2H, overlapped; δ_C_ 129.0) and 6.57 (2H, d, *J =* 8.1 Hz; δ_C_ 112.7)] and the amine proton at δ_H_ 8.00 (6′′′-NH) have HMBC correlations with the carbonyl carbon at δ_C_ 166.0 (C-1′′′′), which in turn showed long-range coupling with the α-proton at 4.82 (H-2′′′) of the β-methoxyasparagine unit (Figure 2). These HMBC data indicated that the terminal *para*-aminobenzoic acid unit of **2** was absent in **3**. The remaining structural fragments were confirmed by the HMBC spectrum (Figure 2). Thus, the planar structure of **3** was identified as that of a new derivative of **2** without the terminal *para*-aminobenzoic acid unit.

The relative configuration of the β-methoxyasparagine moiety of **3** was determined based on ^3^*J*_HH_ and ^2^*J*_CH_ coupling constants and NOESY and NOE differential spectra (Table 1 and Figures S34–S36). H-2′′′ and H-3′′′ were *anti* to each other based on their large ^3^*J_HH_* coupling constant (8.0 Hz). On the basis of the large ^2^*J*_CH_ coupling constant (6.09 Hz) between H-2′′′ and C-3′′, H-2′′′ and OMe are in a *gauche*. The NOEs between H-3′′′ and NH-6′′′, from NH-8′′ to H-3′′′ and OMe, and from H-2′′′ to OMe supported the same relative stereochemistry as **2**. The Cotton effects [[*θ*]^25^(nm)(MeOH): −707 (260), 976 (301)] of **3** were almost the same as those [[*θ*]^25^(nm)(MeOH): −1278 (256), 1301 (313)] of **2** (Figure S9). Thus, **3** was determined to be a new derivative of **2** without the terminal *para*-aminobenzoic acid unit.

**Figure 3:**
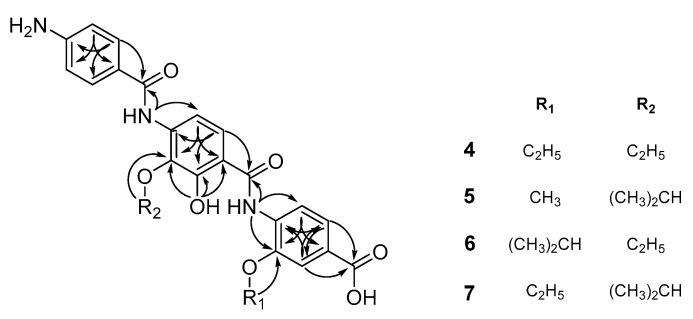
Key HMBC and COSY correlations of **4**–**7**.

Compound **10** was identified as cystobactamid 507 [5] based on its ESIMS data in combination with its ^1^H and ^13^C NMR data (Figures S37–S40 and Table 4 and Table 5). The molecular formula of **4** was determined to be C_25_H_25_O_7_N_3_ based on its HRESIMS data in combination with its ^1^H and ^13^C NMR spectra (Figures S41–S44 and Table 4 and Table 5). The ^1^H NMR data acquired in CD_3_OD revealed the presence of nine aromatic protons, two oxygenated methylenes at δ_H_ 4.29 (2H, q, *J* = 7.0 Hz) and 4.16 (2H, q, *J* = 7.0 Hz), and two methyl groups at δ_H_ 1.58 (3H, t, *J* = 7.0 Hz) and 1.45 (3H, t, *J* = 7.0 Hz) (Table 4). The ^1^H-NMR and HMQC spectra of **4** indicated the presence of 1,4-disubstituted benzene, 1,2,4-trisubstituted benzene, 1,2,3,4-tetrasubstituted benzene, and two ethoxy groups. This result suggested the presence of two ethoxy groups in **4** instead of the two isopropoxyl groups seen in **10**. This was confirmed by the HMBC correlations from the methylene protons at δ_H_ 4.16 of one ethoxy group to the carbon at δ_C_ 137.9 of the 1,2,4-trisubstituted benzene and from the methylene protons at δ_H_ 4.02 of the other ethoxy group to the carbon at δ_C_ 148.0 of the 1,2,4-trisubstituted benzene (Figure 3). Thus, **4** was determined to be a new, C-4 and C-4′ diethoxylated derivative at of **10**.

The molecular formula of **5** was determined to be C_25_H_25_O_7_N_3_ based on its HRESIMS data in combination with its ^1^H and ^13^C NMR data (Figures S45–S48 and Table 4 and Table 5). The ^1^H NMR and HMQC data of **5** were similar to those of **10**. The differences were that a signal for a methoxy group at δ_H_ 3.97 was observed instead of signals of an isopropoxy group. The location of the methoxy group was determined based on the HMBC correlation from the methoxy proton signal to the carbon at δ_C_ 149.4 of the 1,2,4-trisubstituted benzene (Figure 3). Thus, **5** was determined to be a new, C-4 methoxylated derivative of **10**.

The molecular formula of **6** was determined to be C_25_H_25_O_7_N_3_ based on its HRESIMS data in combination with ^1^H and ^13^C NMR data (Figures S49–S52 and Table 4 and Table 5). The NMR spectra of **6** were similar to those of **10** except for the presence of ethoxy signals [δ_H_ 4.02 (1H, q, *J =* 7.0 Hz; δ_C_ 68.9) and 1.34 (3H, t, *J =* 7.0 Hz; δ_C_ 15.6)] instead signals for an isopropyl fragment. In the HMBC spectrum, the methylene protons at δ_H_ 4.02 of the ethoxy group showed long-range coupling to the carbon at δ_C_ 138.6, which in turn was correlated with the amine proton at δ_H_ 9.14 and the aromatic proton at δ_H_ 7.68 (Figure 3). These correlations indicated the linkage of the ethoxy group to C-3 of the 1,2,3,4-tetrasubstituted benzene. The HMBC spectrum confirmed the remaining structural features of **6**. Thus, **6** was elucidated as a new, C-4′ ethoxylated derivative at of **10**.

The molecular formula of **7** was determined to be C_25_H_25_O_7_N_3_ based on its HRESIMS data in combination with its ^1^H and ^13^C NMR data (Figures S53–S58 and Table 4 and Table 5). The NMR spectra of **7** were similar to those of **6**. The major difference in the ^1^H NMR and HMQC data were that the methine proton of the isopropyl group of **6** was downfield-shifted from δ_H_ 4.77 to 4.36 in the spectra of **7**. This result suggested that the isopropyl group might be connected to the 1,2,3,4-tetrasubstituted benzene as it is in **5**. HMBC correlations were observed from the methine proton of the isopropyl group to the carbon at δ_C_ 138.1 (C-4′) of the 1,2,3,4-tetrasubstituted benzene and from the methylene proton of the ethoxy group to the carbon at δ_C_ 148.3 (C-4) of 1,2,4-trisubstituted benzene (Figure 3). The HMBC spectrum confirmed the remaining structural features of **7**. Thus, **7** was determined to be as a new, C-4 ethoxylated derivative of **10**.

### 2.2. Antibacterial and E. Coli DNA Gyrase-Inhibitory Activities of the New Compounds

The antibacterial activities of **1**–**10** against clinically important Gram-positive and Gram-negative pathogens were evaluated compared with those of **11**–**13** and ciprofloxacin (Table 6 and Table S1). Additionally, their effects on the supercoiling activity of *E. coli* DNA gyrase were investigated using ciprofloxacin and nalidixic acid, as positive controls, (Figure S59 and Table 7) because the main target of **11** was reported to be DNA gyrase [5]. Compound **1,** the β-methoxyaspartic acid derivative of **11,** showed weaker antibacterial activity against Gram-positive bacteria (MICs of 8–4 μg/mL) compared with that of **11**, and it exhibited no antibacterial activity against both Gram-negative bacteria at 32 μg/mL. However, **1** showed stronger *E. coli* DNA gyrase inhibition (IC_50_ of 0.42 μM) than was observed in **11** (0.95 μM). Considering that **13**, the stereoisomer of **1**, retained antibacterial activity against *E. coli*, these results suggested that the β-methoxyasparagine moiety and its stereochemistry in **11** influences cellular uptake in both Gram-positive and Gram-negative bacteria. The stronger antibacterial activity against *E. coli* of **12** could be due to its stronger *E. coli* DNA gyrase inhibition than **11**. Compound **2**, **8**, and **9** showed antibacterial activities similar to that of **11** against both Gram-positive and Gram-negative bacteria except against *P. aeruginosa* and *K. pneumonia*. Compound **2**, with an amino moiety instead of the nitro moiety on the *para*-nitrobenzoic acid unit (relative to **11**), did not inhibit the growth of *P. aeruginosa* or *K. pneumonia* at 32 μg/mL, while **8** and **9,** the derivatives in which the isopropyl chain at C-4 of **11** has been shortened, showed weak antibacterial activities against *P. aeruginosa* but did not inhibit the growth of *K. pneumonia* at 32 μg/mL. However, **2**, **8**, and **9** showed strong *E. coli* DNA gyrase inhibition with IC_50_ values of 0.05–0.51 μM, making them more potent than **11**. Considering that **11** was reported to inhibit DNA gyrases of both *E. coli* and *P. aeruginosa* with similar potencies [9], these results suggested that the nitro group and the isopropyl chain could be important for permeability into *P. aeruginosa* and *K. pneumonia.* Another explanation of the discrepancy between *E. coli* DNA gyrase inhibition and antibacterial activity of these respective compounds might also be due to an improved efflux. 

On the other hand, **3**, a derivative of **11** with the *para*-nitrobenzoic acid unit removed, exhibited no antibacterial activities against any of the tested bacterial strains at 32 μg/mL, which was consistent with its dramatically lower ability to inhibit DNA gyrase (IC_50_ of 39.1 μM). These results suggested that the *para*-aminobenzoic acid unit is critical to the inhibition of DNA gyrase and bacterial growth. Compounds **4**–**7** and **10**, derivatives missing the *para*-aminobenzoic acid unit and β-methoxyasparagine moiety, exhibited no antibacterial activities against any of the tested bacterial strains at 32 μg/mL and showed no DNA gyrase inhibition even at 300 μM. Although **10** was reported to show weak antibacterial and DNA gyrase-inhibitory activities against Gram-negative and Gram-positive bacteria (MICs of 4–65 μg/mL and IC_50_ of 20.2 μM, respectively) [5], **10** and its derivatives **4**–**7** did not display those activities in this study.

## 3. Materials and Methods

### 3.1. General Experimental Procedures

Optical rotations were determined on a JASCO P-1020 polarimeter (JASCO, Tokyo, Japan). UV spectra were measured on a Shimadzu UV-1601 UV−visible spectrophotometer (Shimadzu, Kyoto, Japan). IR spectra were obtained using a Bruker EQUINOX 55 spectrometer (Bruker Co., Ettlingen, Germany). NMR spectra were recorded on Bruker Biospin Avance 400, 500, 700, 800, or 900 MHz spectrometers (Korea Basic Science Institute, Ochang, Korea). HRESIMS data were recorded on a JEOL JMSHX110/110A mass spectrometer (JEOL, Tokyo, Japan).

### 3.2. Fermentation and Isolation

The 3000-L fermentation was carried out in a 5000-L fermenter using seed cultures from sequential 500-mL Erlenmeyer flask and 5-L, 50-L, and 500-L fermenters ((Supporting Information). The resin and cells were recovered and extracted twice with 100% acetone. The acetone was evaporated from the extract, and the remaining aqueous phase was extracted sequentially with chloroform and ethyl acetate. The ethyl acetate extract (8.1 g) was applied to Sephadex LH-20 column and eluted with MeOH to give four major fractions, Fr. I to IV, based on TLC analysis. Fr. II was purified by thin-layer chromatography (TLC) on silica gel 60 F_254_ plates (Merck No. 1.05715.0001, Darmstadt, Germany) with CHCl_3_:MeOH = 3:1 to give two major bands, Bands I and II, at *R_f_* values of 0.3 and 0.8, respectively. Band I was further purified by TLC on silica gel 60 RP-18 F_254_ plates (Merck No. 1.15389.0001) with CH_3_CN:H_2_O (55:45) to yield **13** (2.1 mg) and **1** (2.2 mg) at *R_f_* values of 0.60, and 0.64, respectively. In the same way, **12** (22.1 mg) was purified from band II at an *R_f_* value of 0.5. Compounds **11** (657 mg) and **12** (2.8 mg) were obtained from Fr. III by silica gel TLC with CHCl_3_:MeOH = 6:1. Fr. IV was also purified by silica gel TLC with CHCl_3_−MeOH (3:1) and then subjected to preparative HPLC (YMC column 150 × 20 mm I.D., flow rate 3.0 mL/min) eluting with MeOH:H_2_O (50:50) to afford **2** (4.1 mg), **8** (3.2 mg), **9** (2.2 mg)**,** and **11** (8.1 mg) at retention times of 19, 33, 43, and 52 min, respectively. The chloroform extract (500 g) was separated on a silica gel column eluted with a stepwise gradient of MeOH in CHCl_3_ containing 0.01% trifluoroacetic acid (TFA) to give 15 fractions, Fr. 1–16. Fr. 6 was purified by preparative HPLC (OP C18-101002510 column 250 × 10 mm I.D., flow rate 2.5 mL/min) eluting with CH_3_CN:H_2_O (50:50) containing 0.01% TFA to afford **4** (2.4 mg), **5** (3.5 mg), **6** (3.1 mg), **7** (6.2 mg)**,** and **10** (12.3 mg) at retention times of 12.5, 14.0, 15.0, 18.5, and 22.0 min, respectively. Fr. 9 was purified on a Sephadex LH-20 column eluted with MeOH, and then purified by RP-18 TLC developed with MeOH:H_2_O (70:30) containing 0.01% TFA to yield **11** (7.1 mg), **10** (3.2 mg), and **3** (8.4 mg) at *R_f_* values of 0.17, 0.30, and 0.38, respectively.

Compounds **1**, **2**, **3**, **8**, **9, 11**, **12**, and **13** appeared as single peaks at 220 nm using an analytical HPLC column (4.6 × 150 mm, S-4 μm, YMC C18) with CH_3_CN:H_2_O (50:50) containing 0.01% TFA at a flow rate of 0.8 mL/min with retention times of 18.2, 6.0, 5.1, 9.5, 12.8, 15.1, 19.8, and 18.3 min, respectively (Figure S60). Compounds **4**, **5**, **6**, **7**, and **10** were also determined to be single peaks under the HPLC conditions described above with retention times of 9.0, 9.9, 10.8, 13.6, and 16.7 min, respectively (Figure S61). 

*Coralmycin C* (compound **1**): yellow powder; ${\left[\alpha \right]}_{D}^{25}$ = +24.8 (*c* 0.064, MeOH); UV (MeOH) *λ*_max_(log ε) 213 (4.80), 266 (sh) (4.13), 301 (4.26), 319 (4.23) nm; IR (KBr) ν_max_ 3425, 2918, 2850, 1684, 1516, 1431, 1274, 1205, 1138, 1050, 1027, 1002 cm^−1^; CD (MeOH) [θ]^25^ −415 (250), 345 (304) nm; HRESIMS *m/z* 921.2941 (M + H)^+^ (cald for C_46_H_45_N_6_O_15_, 921.2937).

*Coralmycin D* (compound **2**): yellow powder; ${\left[\alpha \right]}_{D}^{25}$ = +16.0 (*c* 0.032, MeOH); UV (MeOH) *λ*_max_(log ε) 212 (4.73), 306 (4.24), 314 (4.24) nm; IR (KBr) 3424, 2917, 2850, 1682, 1602, 1512, 1428, 1272, 1203, 1138, 1026, 999 cm^−1^; CD (MeOH) [θ]^25^ −1278 (256), 1301 (313) nm; HRESIMS *m/z* 890.3415 (M + H)^+^ (cald for C_46_H_48_N_7_O_12,_ 890.3362).

*Coralmycin E* (compound **3**): yellow powder; ${\left[\alpha \right]}_{D}^{25}$ = −20.0 (*c* 0.064, MeOH); UV (MeOH) *λ*_max_(log ε) 212 (4.56), 266 (sh) (3.99), 298 (4.17), 320 (4.12) nm; IR (KBr) 3426, 2958, 2918, 2850, 1683, 1603, 1538, 1432, 1277, 1204, 1139, 1052, 1027 cm^−1^; CD (MeOH) [θ]^25^ −707 (260), 976 (301) nm; HRESIMS *m/z* 771.2995 (M + H)^+^ (cald for C_39_H_43_N_6_O_11,_ 771.2984).

*Coralmycin F* (compound **4**): yellow powder; UV (MeOH) *λ*_max_(log ε) 212 (4.30), 263 (sh) (3.87), 323 (3.93) nm; IR (KBr) 2927, 1686, 1497, 1205 cm^−1^; HRESIMS *m/z* 480.1769 (M + H)^+^ (cald for C_25_H_26_N_3_O_7,_ 480.1765).

*Coralmycin G* (compound **5**): yellow powder; UV (MeOH) *λ*_max_(log ε) 210 (4.38), 263 (sh) (3.81), 318 (3.96) nm; IR (KBr) 3426, 2974, 2930, 1685, 1599, 1506, 1421, 1275, 1206, 1181, 1136 cm^−1^; HRESIMS *m/z* 480.1748 (M + H)^+^ (cald for C_25_H_26_N_3_O_7,_ 480.1765).

*Coralmycin H* (compound **6**): yellow powder; UV (MeOH) *λ*_max_(log ε) 213 (4.53), 263 (sh) (3.88), 325 (4.03) nm; IR (KBr) 3424, 2977, 2928, 1685, 1599, 1509, 1427, 1271, 1202, 1137, 1007 cm^−1^; HRESIMS *m/z* 494.1906 (M + H)^+^ (cald for C_26_H_28_N_3_O_7,_ 494.1922).

*Coralmycin I* (compound **7**): yellow powder; UV (MeOH) *λ*_max_(log ε) 210 (4.38), 263 (sh) (3.86), 319 (3.97) nm; IR (KBr) 3422, 2976, 2929, 1686, 1645, 1598, 1503, 1424, 1275, 1186, 1155, 1105, 1025, 1001 cm^−1^; HRESIMS *m/z* 494.1904 (M + H)^+^ (cald for C_26_H_28_N_3_O_7,_ 494.1922).

### 3.3. Determination of Antibacterial Susceptibility 

Whole-cell antimicrobial activities were determined using a broth microdilution method described previously [10]. Most of the test strains were grown to mid-log phase in Mueller–Hinton broth and diluted 1,000-fold in the same medium. Cells (10^5^/mL) were dispensed at 0.2 mL/well in 96-well microtiter plates. *Streptococcus pneumonia* and *Acinetobacter baumanii* were grown in Todd–Hewitt medium and nutrient broth, respectively. The test compounds and ciprofloxacin (Sigma St. Louis, MO, USA) were soluble in DMSO, the final concentration of which did not exceed 0.05% in the cells. Cells were treated with 0.05% DMSO as a vehicle control. The MICs were determined in triplicate by serial two-fold dilutions of the test compounds. The MIC was defined as the concentration of a test compound that completely inhibited cell growth during a 24-h incubation period at 37 °C. Bacterial growth was determined by measuring the absorption at 650 nm using a microtiter enzyme-linked immunosorbent assay (ELISA) reader (Molecular Devices Corporation, Sunnyvale, CA, USA).

### 3.4. DNA Gyrase Assay 

The inhibitory activities of coralmycins on the supercoiling activity of DNA gyrase were investigated using a ‘Purified *E. coli* DNA Gyrase Drug Screening Kit’ (TopoGEN #TG2001G, Buena Vista, CO, USA). The test compounds, ciprofloxacin (200–0.02 μM) and nalidixic acid (Sigma) (200–0.02 μM) were soluble in DMSO, the final concentration of which did not exceed 0.05% in the reactions (Figure S59). Relaxed plasmid (0.5 μg) was mixed with one unit of gyrase (~20.5 nM) and 1 μL of the test compound in the reaction buffer (final volume of 20 μL) and incubated at 37 °C for 30 min. The reaction was terminated by the addition of 2 μL of 10% (*w/v*) SDS and 2 μL of 10× DNA gel loading dye (bromophenol blue). Then, 20 μL of chloroform:isoamyl alcohol (24:1 mixture) was added, and the mixture was vortexed and then centrifuged. The samples in the blue phase were run on 1% (*w/v*) agarose gel and were visualized using ethidium bromide. All reactions were performed in triplicate. To calculate the IC_50_ values, the band densities were measured using ImageJ (National Institutes of Health, Bethesda, Maryland, MD, USA).

## 4. Conclusions

Seven new derivatives, **1**–**7**, were isolated from a large-scale culture of the myxobacteria *Corallococcus coralloides* M23. Compound **2** showed potent antibacterial activity against both Gram-positive and Gram-negative bacteria except against *P. aeruginosa* and *K. pneumonia* with potent DNA gyrase inhibition. Interestingly, **1** exhibited antibacterial activity against Gram-positive bacteria but not against Gram-negative bacteria, although it potently inhibited DNA gyrase. Their structure-activity relationships between the antibacterial activity and DNA gyrase inhibitory activity indicated that the para-nitrobenzoic acid unit is critical for the inhibition of both DNA gyrase and bacterial growth, while the nitro moiety of the para-nitrobenzoic acid unit and the isopropyl chain at C-4 could be important for the permeability of the compound into certain Gram-negative bacteria, including *P. aeruginosa* and *K. pneumonia*, and the β-methoxyasparagine moiety could influence cellular uptake into all tested bacteria. 

## Figures and Tables

**Table 1:** ^1^H NMR data (500 MHz, DMSO-*d_6_*) for **1**–**3**.

Position	11	1	2	3	8	9
1	-	-	-	-	-	-
2	-	-	-	-	-	-
3	7.56, brs	7.57, s	7.59, s	7.58 *	7.57, s	7.55, s
4	-	-	-	-	-	-
5	-	-	-	-	-	-
6	8.50, d (8.3)	8.51, d (8.3)	8.52, d (8.4)	8.51, d (8.2)	8.39, d (8.3)	8.46, d (8.3)
7	7.58, d (8.3)	7.59, d (8.7)	7.60, d (8.3)	7.58 *	7.62, d (8.3)	7.61, d (8.3)
8	4.75, m	4.75, m	4.76, m	4.75, m	3.97, s	4.2, q (7.0)
9,10	1.37, d (6.0)	1.37, d (6.0)	1.38, d (6.0)	1.37, d (5.8)	-	1.46, t (7.1)
11-NH	10.97, brs	-	11.00, s	11.00, s	10.89, s	10.96, s
1′	-	-	-	-	-	-
2′	-	-	-	-	-	-
3′	-	-	-	-	-	-
3′-OH	11.21, brs	11.30, s	11.27, s	11.27, s	11.46, s	11.31, s
4′	-	-	-	-	-	-
5′	-	-	-	-	-	-
6′	7.50, d (8.5)	7.52, d (8.7)	7.54, d (8.8)	7.52, d (8.7)	7.55, m	7.54, d (8.7)
7′	7.80, d (8.5)	7.81, d (8.7)	7.84 *	7.81 *	7.82, m	7.82, d (7.6)
8′	4.32, m	4.31, m	4.33, m	4.30, m	4.38, m	4.33, m
9′,10′	1.26, d (6.1)	1.26, d (6.1)	1.28, d (6.1)	1.26, d (6.0)	1.27, d (6.4)	1.26, d (6.1)
11′-NH	9.58, s	9.60, s	9.61, s	9.60, s	9.58, s	9.59, s
1′′	-	-	-	-	-	-
2′′	-	-	-	-	-	-
3′′,7′′	7.97, d (8.6)	7.97, d (8.5)	7.98, d (8.8)	7.96, (8.3)	7.98, d (8.7)	7.97, d (8.8)
4′′,6′′	7.83, d (8.6)	7.84, d (8.5)	7.84 *	7.81 *	7.83, m	7.83, d (7.6)
5′′	-	-	-	-	-	-
8′′-NH	10.56, s	10.52, s	10.57, s	10.50, s	10.57, s	10.57, s
1′′′	-	-	-	-	-	-
2′′′	4.92, dd (8.0, 8.1)	5.07, t (8.3)	4.92, t (8.1)	4.82, t (7.9)	4.92, t (8.1)	4.92, t (8.1)
3′′′	4.09, d (8.0)	4.16, d (8.1)	4.11, d (8.0)	4.06, d (8.0)	4.09, d (8.1)	4.09, d (8.1)
4′′′-NH	7.47, brs 7.54, brs	-	7.48, brs	7.45, brs	7.48, brs	7.48, brs
5′′′	3.31, s	3.35, s	3.32, s	3.28, s	3.31, s	3.31, s
6′′′-NH	8.46, d (8.1)	8.72, d (8.5)	8.41, d (8.2)	8.00, d (8.0)	8.47, d (8.1)	8.47, d (8.1)
1′′′′	-	-	-	-	-	-
2′′′′	-	-	-	-	-	-
3′′′′,7′′′′	7.90, d *	7.94, d (8.6)	7.84 *	7.58 *	7.91 *	7.91 *
4′′′′,6′′′′	7.90, d *	7.90, d (8.6)	7.84 *	6.57, d (8.1)	7.91*	7.91*
5′′′′	-	-	-	-	-	-
8′′′′-NH	10.8, s	10.84, s	10.02, s	-	10.81, s	10.81, s
1′′′′′	-	-	-	-	-	-
2′′′′′	-	-	-	-	-	-
3′′′′′,7′′′′′	8.21, d (8.6)	8.21, d (8.6)	7.75, d (8.8)	-	8.21, d (8.8)	8.21, d (8.8)
4′′′′′,6′′′′′	8.38, d (8.6)	8.38, d (8.6)	6.62, d (8.6)	-	8.39, d (8.8)	8.39, d (8.8)
5′′′′′	-	-	-	-	-	-

* Overlapped.

**Table 2:** ^13^C NMR data (500 MHz, DMSO-*d_6_*) for **1**–**3**.

Position	11	1	2	3	8	9
1	166.9, C	167.4, C	167.3, C	167.0, C	166.8, C	166.9, C
2	125.7, C	126.2, C	126.2, C	125.8, C	126.1, C	126.3, C
3	113.9, CH	114.3, CH	114.4, CH	113.9, CH	111.7, CH	111.9, CH
4	146.3, C	146.8, C	146.8, C	146.4, C	148.4, C	148.0, C
5	133.3, C	133.7, C	133.4, C	133.3, C	131.4, C	132.6, C
6	119.6, CH	120.0, CH	120.1, CH	119.6, CH	120.4, CH	119.6, CH
7	122.6, CH	123.1, CH	123.0, CH	122.7, CH	122.4, CH	122.6, CH
8	71.7, CH	72.1, CH	72.2, CH	71.8, CH	56.5, CH_3_	64.1, CH_2_
9,10	21.6, CH_3_	22.0, CH_3_	22.0, CH_3_	21.7, CH_3_	-	14.3, CH_3_
1′	163.6, C	164.0, C	164.0, C	163.6, C	164.2, C	136.6, C
2′	116.4, C	116.9, C	117.0, C	116.6, C	115.5, C	116.4, C
3′	150.3, C	150.9, C	150.9, C	150.4, C	150.8, C	150.3, C
4′	138.4, C	138.9, C	139.0, C	138.5, C	138.5, C	138.4, C
5′	136.2, C	136.7, C	136.6, C	136.3, C	136.0, C	136.2, C
6′	115.3, CH	115.8, CH	115.6, CH	115.3, CH	115.1, CH	114.9, CH
7′	124.9, CH	125.4, CH	125.5, CH	125.0, CH	125.0, CH	124.9, CH
8′	75.6, CH	76.1, CH	76.1, CH	75.7, CH	75.1, CH	75.6, CH
9′,10′	22.0, CH_3_	22.3, CH_3_	22.3, CH_3_	22.0, CH_3_	21.8, CH_3_	22.0, CH_3_
1′′	164.3, C	164.8, C	164.8, C	164.4, C	164.1, C	164.7, C
2′′	128.6, C	129, C	129.3, C	128.5, C	128.3, C	128.6, C
3′′,7′′	128.4, CH	128.9, CH	128.9, CH	128.5, CH	128.3, CH	128.4, CH
4′′,6′′	118.8, CH	119.4, CH	119.3, CH	118.8, CH	118.6, CH	118.8, CH
5′′	142.1, C	142.5, C	142.6, C	142.2, C	142.0, C	142.1, C
1′′′	168.6, C	168.6, C	169.2, C	169.2, C	168.6, C	168.6, C
2′′′	55.7, CH	56.0, CH	56.1, CH	55.7, CH	55.6, CH	55.5, CH
3′′′	79.7, CH	80.2, CH	80.5, CH	80.0, CH	79.9, CH	79.8, CH
4′′′	170.8, C	171.4, C	171.3, C	171.0, C	171.2, C	170.8, C
5′′′	57.7, CH_3_	58.6, CH_3_	58.1, CH_3_	57.6, CH_3_	57.5, CH_3_	57.6, CH_3_
1′′′′	165.4, C	166.2, C	166.0, C	166.0, C	165.5, C	165.4, C
2′′′′	128.9, C	129.4, C	129.3, C	120.3, C	128.5, C	128.9, C
3′′′′,7′′′′	128.2, CH	129.0, CH	129.9, CH	129.0, CH	128.4, CH	128.2, CH
4′′′′,6′′′′	119.6, CH	120.0, CH	119.7, CH	112.7, CH	119.5, CH	117.6, CH
5′′′′	141.7, C	142.1, C	143.3, C	151.8, C	141.7, C	141.7, C
1′′′′′	164.2, C	164.6, C	165.9, C	-	164.0, C	164.2, C
2′′′′′	140.4, C	140.7, C	120.9, C	-	140.2, C	140.4, C
3′′′′′,7′′′′′	129.3, CH	129.8, CH	129.9, CH	-	129.2, CH	129.3, CH
4′′′′′,6′′′′′	123.5, CH	124.0, CH	113.1, CH	-	123.5, CH	123.5, CH
5′′′′′	149.2, C	149.7, C	152.8, C		149.1, C	149.2, C

**Table 3:** Comparison of the NMR data of the β-methoxyasparagine moiety of **1** with those of **11** and **13** (DMSO-*d_6_*).

Position	11		13		1	
	δ_C_	δ_H_	δ_C_	δ_H_	δ_C_	δ_H_
1′′′	168.6, C	-	168.7, C	-	168.6, C	-
2′′′	55.7, CH	4.92, t (8.0)	54.9, CH	4.87, brs	56.0, CH	5.07, t (8.3)
3′′′	79.7, CH	4.09, d (8.0)	82.6, CH	4.27, brs	80.2, CH	4.16, d (8.1)
4′′′	170.8, C	-	170.1, C	-	171.4, C	-
4′′′-NH_a_	-	7.47, s	-	-	-	-
4′′′-NH_b_	-	7.54, s	-	-	-	-
5′′′	57.7, CH_3_	3.31, s	58.9, CH_3_	3.44, s	58.6, CH_3_	3.35, s
6′′′-NH	-	8.46, d (8.1)	-	8.37, brs	-	8.72, d (8.5)

**Table 4:** ^1^H NMR data for **4**–**7**.

Position	10 ^a^	4 ^a^	5 ^b^	6 ^b^	7 ^b^
1		-	-	-	-
2		-	-	-	-
3	7.68, s	7.68, d (1.6)	7.59, d (1.7)	7.58, d (1.5)	7.55, d (1.7)
4		-	-	-	-
5		-	-	-	-
6	8.50, d (8.8)	8.53, d (8.4)	8.36, d (8.3)	8.54, d (8.3)	8.44, d (8.4)
7	7.69, d (7.4)	7.71, dd (1.7, 8.4)	7.63, dd (1.7, 8.3)	7.60, dd (1.7, 8.4)	7.61, dd (1.7, 8.4)
8	4.80, m	4.29, q (7.0)	3.97, s	4.77, m	4.21, q (6.9)
9,10	1.48, d (8.0)	1.58, t (7.0)	-	1.39, d (6.0)	1.47, t (6.9)
11-NH		-	10.83, s	10.96, s	10.93, s
1′		-	-	-	-
2′		-	-	-	-
3′		-	-	-	-
3′-OH		-	11.51, s	11.32, s	11.34, s
4′		-	-	-	-
5′		-	-	-	-
6′	7.80, d (8.6)	7.83, overlapped	7.69, d (8.8)	7.68, d (8.9)	7.64, d (8.8)
7′	7.77, d (9.0)	7.83, overlapped	7.81, d (8.8)	7.80, d (8.9)	7.81, d (8.8)
8′	4.55, m	4.16, q (7.0)	4.42, m	4.02, q (7.0)	4.36, m
9′,10′	1.37, d (6.14)	1.45, t (7.0)	1.28, d (6.1)	1.34, t (7.0)	1.28, d (6.2)
11′-NH		-	9.10, s	9.14, s	9.25, s
1′′		-	-	-	-
2′′		-	-	-	-
3′′,7′′	7.78, d (4.8)	7.77, d (8.7)	7.70, d (8.7)	7.71, d (8.7)	7.77, d (8.6)
4′′,6′′	6.88, d (8.5)	6.79, d (8.7)	6.64, d (8.6)	6.64, d (8.7)	6.81, d (8.2)
5′′		-	-	-	-

^a^ measured in CD_3_OD at 500 MHz; ^b^ measured in DMSO-*d_6_* at 500 MHz.

**Table 5:** ^13^C NMR data for **4**–**7**.

Position	10 ^a^	4 ^a^	5 ^b^	6 ^b^	7 ^b^
1	169.5, C	167.8, C	167.4, C	167.5, C	167.4, C
2	127.3, C	125.9, C	126.9, C	126.0, C	126.3, C
3	116.3, CH	111.8, CH	111.7, CH	114.3, CH	112.6, CH
4	148.4, C	148.0, C	149.4, C	146.4, C	148.3, C
5	134.4, C	132.1, C	131.9, C	133.8, C	132.5, C
6	121.2, CH	119.5, CH	120.8, CH	120.0, CH	120.4, CH
7	124.4, CH	122.7, CH	123.0, CH	122.9, CH	122.8, CH
8	73.3, CH	64.5, CH_2_	56.5, CH_3_	72.1, CH	64.8, CH_2_
9	22.3, CH_3_	13.5, CH_3_	-	22.0, CH_3_	14.9, CH_3_
10	22.3, CH_3_	-	-	-	-
1′	167.6, C	165.2, C	165.0, C	164.1, C	164.6, C
2′	116.4, C	114.9, C	114.8, C	116.0, C	116.0, C
3′	151.5, C	150.7, C	151.4, C	150.3, C	151.0, C
4′	138.3, C	137.9, C	137.9, C	138.6, C	138.1, C
5′	138.1, C	136.5, C	136.9, C	136.9, C	137.4, C
6′	114.5, CH	112.9, CH	113.9, CH	114.1, CH	114.5, CH
7′	125.5, CH	124.7, CH	125.1, CH	125.9, CH	125.2, CH
8′	77.2, CH	69.0, CH_2_	75.3, CH	68.9, CH	75.8, CH
9′	22.7, CH_3_	14.3, CH_3_	22.3, CH_3_	15.6, CH_3_	22.6, CH_3_
10′	22.7, CH_3_	-	22.3, CH_3_	15.6, CH_3_	22.6, CH_3_
1′′	167.0, C	166.4, C	164.9, C	165.3, C	165.1, C
2′′	124.1, C	121.4, C	120.5, C	120.2, C	122.9, C
3′′,7′′	130.3, CH	128.9, CH	129.5, CH	129.6, CH	129.5, CH
4′′,6′′	115.1, CH	113.7, CH	113.2, CH	113.2, CH	115.2, CH
5′′	152.9, C	152.2, C	152.9, C	152.9, C	150.0, C

^a^ measured in CD_3_OD at 500 MHz; ^b^ measured in DMSO-*d_6_* at 500 MHz.

**Table 6:** Antibacterial activities of **1**–**3** and related compounds.

Test Organisms	MIC (μg/mL)								
	11	12	13	1	2	3	8	9	Cip *
*Staphylococcus aureus* RN 4220	0.125	0.015	2	4	0.25	>32	0.25	0.125	0.125
MRSA CCARM 3167	0.25	0.015	1	4	0.5	>32	0.5	0.25	4
MRSA CCARM 3506	0.25	0.015	1	4	0.5	>32	0.125	0.25	2
QRSA CCARM 3505	1	0.125	-	4	1	>32	2	2	128
QRSA CCARM 3519	1	0.25	-	4	1	>32	1	1	128
*Streptococcus pneumonia* KCTC 5412	1	0.25	>16	4	2	>32	2	1	0.25
*Enterococcus faecalis* KCTC 5191	0.25	0.03	4	8	1	>32	1	0.5	0.5
*Acinetobacter baumannii* KCTC 2508	2	0.125	4	>32	4	>32	1	0.5	0.25
*E. coli* CCARM 1356	2	0.125	16	>32	1	>32	0.5	1	64
*E. coli* KCTC 1682	1	0.125	4	>32	1	>32	0.25	0.5	0.06
*Pseudomonas aeruginosa* KCTC 2004	4	4	>16	>32	>32	>32	4	8	0.03
*Klebsiella pneumoniae* KCTC 22057	4	2	>16	>32	>32	>32	>32	>32	0.015

* Ciprofloxacin.

**Table 7:** The inhibitory activity of **1**–**4** and related compounds on the supercoiling activity of *E. coli* DNA gyrase (IC_50_, μM) and their antibacterial activity on *E. coli* KCTC 1682 (MIC, μg/mL).

	11	12	13	1	2	3	4	8	9	10	Cip
IC_50_	0.95	0.08	1.07	0.42	0.51	39.1	>300	0.05	0.13	>300	0.30
MIC	1	0.25	4	>32	1	>32	>32	0.25	0.25	>32	0.06

## Supplementary Materials

The following are available online: 1D NMR, 2D NMR, and CD spectra (Figures S1–S58) of compounds **1**–**7** and related compounds.
