# Supplementary material for: Coralmycin Derivatives with Potent Anti-Gram Negative Activity Produced by the Myxobacteria Corallococcus coralloides M23
Source: Molecules. 2019 Apr 9;24(7):1390. doi: 10.3390/molecules24071390 (PMC6479757; doi:10.3390/molecules24071390)
Supplement: Supplementary file 1 [file molecules-24-01390-s001.pdf]

Coralmycin derivatives with potent anti-Gram negative activity produced by the myxobacteria *Corallococcus coralloides* M23

Bo-Min Kim,[1,2,#] Nguyen Van Minh,[1,3,#] Ha-Yung Choi,[1,2] and Won-Gon Kim[1,2,*]

[1] Superbacteria Research Center, Korea Research Institute of Bioscience and Biotechnology, Yusong, Daejeon 305-806, Korea

[2] Department of Bio-Molecular Science, KRIBB School of Bioscience, Korea University of Science and Technology (UST), Yusong, Daejeon 305-806, Republic of Korea

[3] College of Pharmacy, Chungnam National University, Daejeon 34134, Republic of Korea.

[#] These authors contributed equally.

* Correspondence: wgkim@kribb.re.kr; Tel.: +82-42-860-4298

Fermentation. Fermentation was carried out in CYS medium containing 0.5% casitone, 0.1% yeast extract, 0.3% soluble starch, 0.1% $MgSO_4 \cdot 7H_2O$, 0.05% $CaCl_2$, 50 mM 4-(2-hydroxyethyl)-1-piperazineethanesulfonic acid (HEPES), 0.4% trace element solution, and 0.5 μg/mL cyanocobalamin. The trace element solution contained 100 mg/L $MnCl_2 \cdot 4H_2O$, 20 mg/L $CoCl_2$, 10 mg/L $CuSO_4$, 10 mg/L $Na_2MoO_4 \cdot 2H_2O$, 20 mg/L $ZnCl_2$, 5 mg/L LiCl, 5 mg/L $SnCl_2 \cdot 2H_2O$, 10 mg/L H_3BO_3, 20 mg/L KBr, 20 mg/L KI, and 8 g/L EDTA Na-Fe^{3+} salt (trihydratepiece of agar from t). The mature plate culture of the producing strain was inoculated into a 500-mL Erlenmeyer flask containing 100 mL of sterile seed liquid medium with the above composition and cultured on a rotary shaker (150 rpm) at 28 °C for 3 days. The 300 mL of the seed culture was transferred into a 5-L Jar fermenter containing 2.5 L of the above medium containing 100 g of Amberlite XAD16 (Sigma, USA), and then cultivated at 28 °C for 2 days with an aeration rate of 0.4 v/min and agitation rate of 350 rpm. The resultant 3 L culture was transferred into a 50-L fermenter containing 30 L of the above medium containing 1.2 kg of Amberlite XAD16, and then cultivated at 28 °C for 3 days with an aeration rate of 0.4 v/min and agitation rate of 200 rpm. The resultant 30 L culture was transferred into a 500-L fermenter containing 300 L of the above medium containing 12 kg of Amberlite XAD16, and then cultivated at 28 °C for 3 days with an aeration rate of 0.4 v/min and agitation rate of 100 rpm. The resultant 300L culture was transferred into a 5000-L fermenter containing 3000 L of the above medium containing 120 kg of Amberlite XAD16, and then cultivated at 28 °C for 6 days with an aeration rate of 0.4 v/min and agitation rate of 70 rpm.

Table S1. Antibacterial activities of **4**–**7** and related compounds.

Test organisms	MIC (μg/mL)					
	**4**	**5**	**6**	**7**	**10**	Cip*
Staphylococcus aureus RN 4220	>32	>32	>32	>32	>32	0.125
MRSA CCARM 3167	>32	>32	>32	>32	>32	4
MRSA CCARM 3506	>32	>32	>32	>32	>32	2
QRSA CCARM 3505	>32	>32	>32	>32	>32	128
QRSA CCARM 3519	>32	>32	>32	>32	>32	128
Streptococcus pneumonia KCTC 5412	>32	>32	>32	>32	>32	0.25
Enterococcus faecalis KCTC 5191	>32	>32	>32	>32	>32	0.5
Acinetobacter baumannii KCTC 2508	>32	>32	>32	>32	>32	0.25
E. coli CCARM 1356	>32	>32	>32	>32	>32	64
E. coli KCTC 1682	>32	>32	>32	>32	>32	0.06
Pseudomonas aeruginosa KCTC 2004	>32	>32	>32	>32	>32	0.03
Klebsiella pneumoniae KCTC 22057	>32	>32	>32	>32	>32	0.015

*Ciprofloxacin

Legends for Figures

Figure S1. Positive HRESIMS spectrum of cystobactamid 891-2 (**8**). 7

Figure S2. ^{1}H-NMR spectrum of cystobactamid 891-2 (**8**) measured in DMSO-d_6 at 500 MHz. 8

Figure S3. HMQC spectrum of cystobactamid 891-2 (**8**) measured in DMSO-d_6 at 500 MHz. 9

Figure S4. HMBC spectrum of cystobactamid 891-2 (**8**) measured in DMSO-d_6 at 500 MHz. 10

Figure S5. Positive HRESIMS spectrum of cystobactamid 905-2 (**9**). 11

Figure S6. ^{1}H-NMR spectrum of cystobactamid 905-2 (**9**) measured in DMSO-d_6 at 500 MHz. 12

Figure S7. HMQC spectrum of cystobactamid 905-2 (**9**) measured in DMSO-d_6 at 500 MHz. 13

Figure S8. HMBC spectrum of cystobactamid 905-2 (**9**) measured in DMSO-d_6 at 500 MHz. 14

Figure S9. CD spectra of coralmycins C (**1**), D (**2**), and E (**3**), and cystobactamids 891-2 (**8**) and 905-2 (**9**). 15

Figure S10. HPLC profiles of coralmycins C (**1**) and B (**13**). A) compound **1**; B) compound **13**; C) mixture of **1** and **13** at ratio of 1:1. 16

Figure S11. Positive HRESIMS spectrum of coralmycin C (**1**). 17

Figure S12. ^{1}H-NMR spectrum of coralmycin C (**1**) measured in DMSO-d_6 at 500 MHz. 18

Figure S13. COSY spectrum of coralmycin C (**1**) measured in DMSO-d_6 at 500 MHz. 19

Figure S14. HMQC spectrum of coralmycin C (**1**) measured in DMSO-d_6 at 500 MHz. 20

Figure S15. HMBC spectrum of coralmycin C (**1**) measured in DMSO-d_6 at 500 MHz. 21

Figure S16. HECADE spectrum of coralmycin C (**1**) measured in DMSO-d_6 at 700MHz. 23

Figure S17. NOESY spectrum of coralmycin C (**1**) measured in DMSO-d_6 at 500 MHz. 233

Figure S18. The NOE differential spectra of coralmycin C (**1**) measured in DMSO-d_6 at 700MHz.. 24

Figure S19. NOE correlations of coralmycin C (**1**) in comparison with cystobactamid 919-2 and coralmycin B 25

Figure S20. Positive HRESIMS spectrum of coralmycin D (**2**). 266

Figure S21. ^{1}H-NMR spectrum of coralmycin D (**2**) measured in DMSO-d_6 at 500 MHz. 277

Figure S22. COSY spectrum of coralmycin D (**2**) measured in DMSO-d_6 at 500 MHz. 288

Figure S23. HMQC spectrum of coralmycin D (**2**) measured in DMSO-d_6 at 500 MHz. 299

Figure S24. HMBC spectrum of coralmycin D (**2**) measured in DMSO-d_6 at 500 MHz. 30

Figure S25. HETLOC spectrum of coralmycin D (**2**) measured in DMSO-d6 at 900MHz. 31

Figure S26. NOESY spectrum of coralmycin D (**2**) measured in DMSO-d_6 at 500 MHz. 32

Figure S27. The NOE differential spectra of coralmycin D (2) measured in DMSO-d6 at 800MHz. 33

Figure S28. Positive HRESIMS spectrum of coralmycin E (3). 34

Figure S29. ^{1}H-NMR spectrum of coralmycin E (**3**) measured in DMSO-d_6 at 500 MHz. 35

Figure S30. ^{13}C-NMR spectrum of coralmycin E (**3**) measured in DMSO-d_6 at 500 MHz. 366

Figure S31. COSY spectrum of coralmycin E (**3**) measured in DMSO-d_6 at 500 MHz. 37

Figure S32. HMQC spectrum of coralmycin E (**3**) measured in DMSO-d_6 at 500 MHz. 38

Figure S33. HMBC spectrum of coralmycin E (**3**) measured in DMSO-d_6 at 500 MHz. 39

Figure S34. HECADE spectrum of coralmycin E (**3**) measured in DMSO-d_6 at 700 MHz. 40

Figure S35. NOESY spectrum of coralmycin E (**3**) measured in DMSO-d_6 at 500 MHz. 41

Figure S36. The NOE differential spectra of coralmycin E (**3**) measured in DMSO-d6 at 800MHz. 42

Figure S37. Positive and negative ESIMS spectrum of cystobactamid 507 (**10**). 43

Figure S38. ^{1}H NMR spectrum of cystobactamid 507 (**10**) measured in CD3OD at 700 MHz. 44

Figure S39. ^{13}C-NMR spectrum of cystobactamid 507 (**10**) measured in CD3OD at 700 MHz. 45

Figure S40. HMBC spectrum of cystobactamid 507 (**10**) measured in CD3OD at 900 MHz. 46

Figure S41. Positive HRESIMS spectrum of coralmycin F (**4**). 47

Figure S42. ^{1}H NMR spectrum of coralmycin F (**4**) measured in CD_3OD at 800 MHz. 48

Figure S43. HMQC spectrum of coralmycin F (**4**) measured in CD_3OD at 500 MHz. 49

Figure S44. HMBC spectrum of coralmycin F (**4**) measured in CD_3OD at 800 MHz. 50

Figure S45. Positive HRESIMS spectrum of coralmycin G (**5**). 51

Figure S46. ^{1}H NMR spectrum of coralmycin G (**5**) measured in DMSO-d_6 at 800 MHz. 52

Figure S47. HMQC spectrum of coralmycin G (**5**) measured in DMSO-d_6 at 800 MHz. 53

Figure S48. HMBC spectrum of coralmycin G (**5**) measured in DMSO-d_6 at 800 MHz. 54

Figure S49. Positive HRESIMS spectrum of coralmycin H (**6**). 55

Figure S50. ^{1}H NMR spectrum of coralmycin H (**6**) measured in DMSO-d_6 at 800 MHz. 56

Figure S51. HMQC spectrum of coralmycin H (**6**) measured in DMSO-d_6 at 800 MHz. 57

Figure S52. HMBC spectrum of coralmycin H (**6**) measured in DMSO-d_6 at 800 MHz. 58

Figure S53. Positive HRESIMS spectrum of coralmycin I (**7**). 59

Figure S54. ^{1}H NMR spectrum of coralmycin I (**7**) measured in DMSO-d_6 at 500 MHz. 60

Figure S55. HMQC spectrum of coralmycin I (**7**) measured in DMSO-d_6 at 500 MHz. 61

Figure S56. Expansion of HMQC spectrum of coralmycin I (**7**). 62

Figure S57. HMBC spectrum of coralmycin I (**7**) measured in DMSO-d_6 at 500 MHz. 63

Figure S58. Expansion of HMBC spectrum of coralmycin I (**7**). 64

Figure S59. Agarose gels of E. coli gyrase supercoiling reactions inhibited by coralmycins (C (**1**), D (**2**), E (**3**), F (**4**), A (**12**), and B (**13**)) and cystobactamids (891-2 (**8**), 905-2 (**9**), 919-2 (**11**), and 507 (**10**)). (-), reaction without E. coli gyrase; (+), standard reaction in presence of 5% DMSO; rel, relaxed plasmid; SC, supercoiled plasmid......... 65

Figure S60. HPLC profile of coralmycins (C (**1**), D (**2**), E (**3**), A (**12**), and B (**13**)) and cystobactamids (891-2 (**8**), 905-2 (**9**), and 919-2 (**11**)). 66

Figure S61. HPLC profile of coralmycins (F (**4**), G (**5**), H (**6**), and I (**7**)) and cystobactamid 507 (**10**). 67

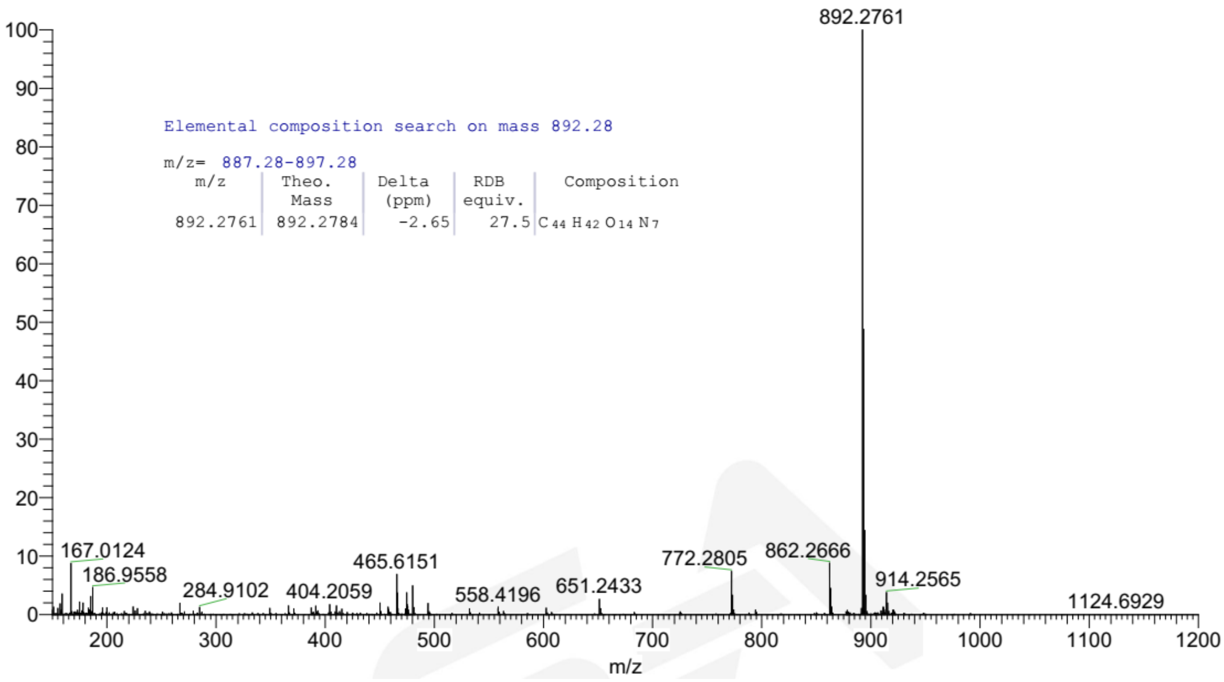

Figure S1. Positive HRESIMS spectrum of cystobactamid 891-2 (**8**).

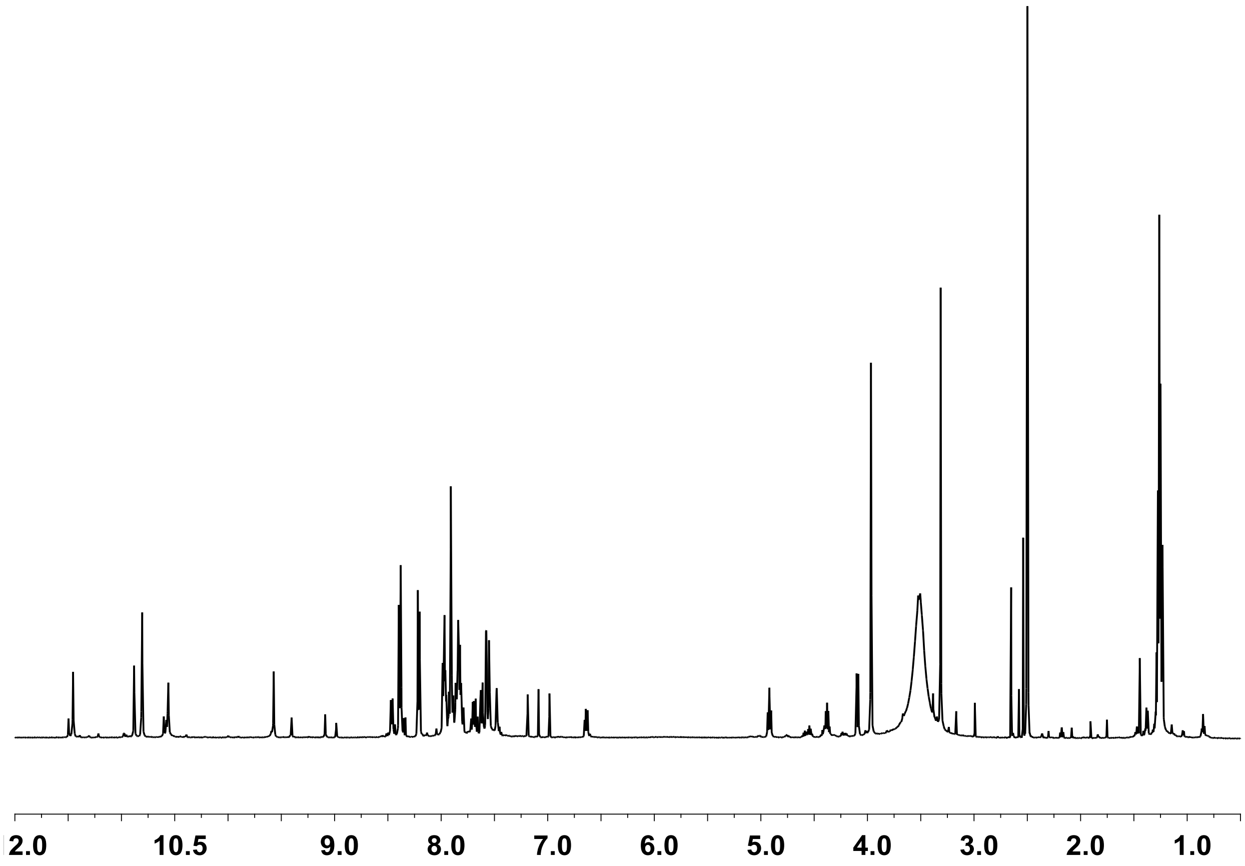

Figure S2. ^{1}H-NMR spectrum of cystobactamid 891-2 (**8**) measured in DMSO-*d*$_6$ at 500 MHz.

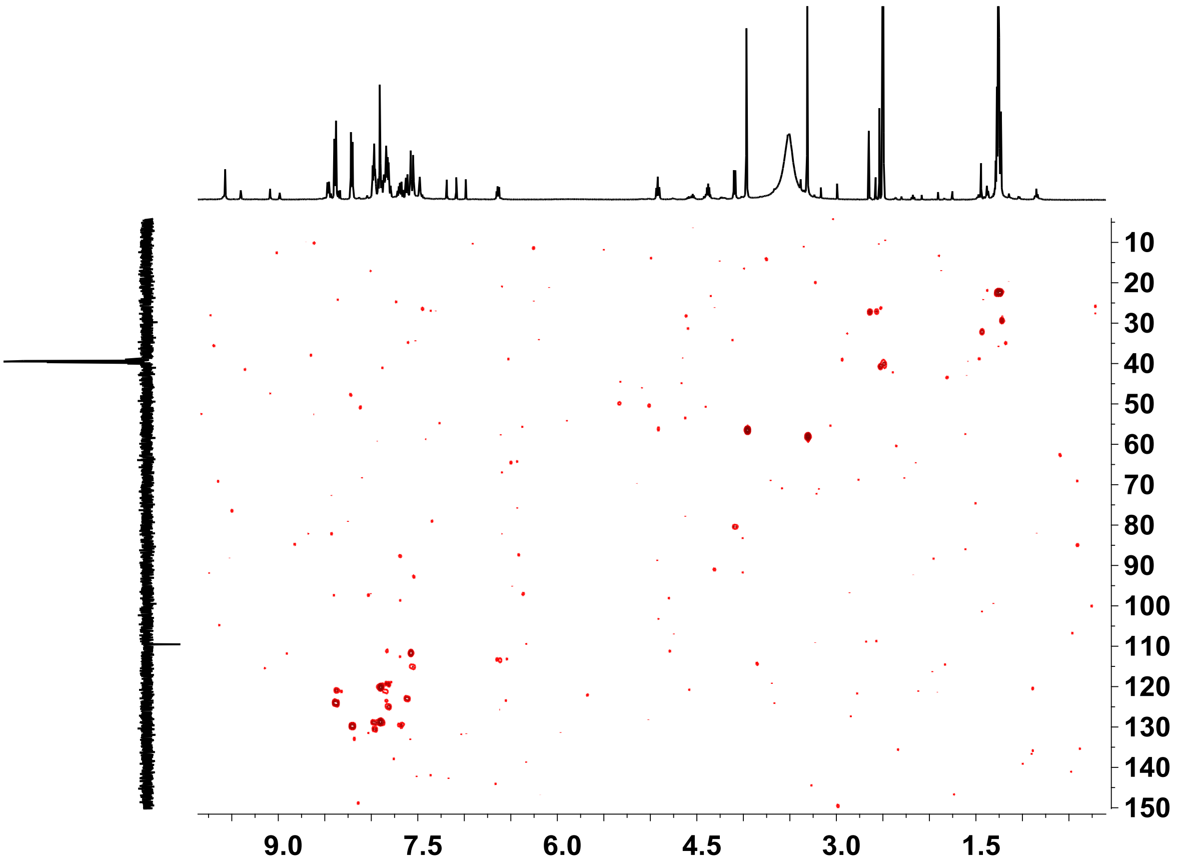

Figure S3. HMQC spectrum of cystobactamid 891-2 (**8**) measured in DMSO-d_6 at 500 MHz.

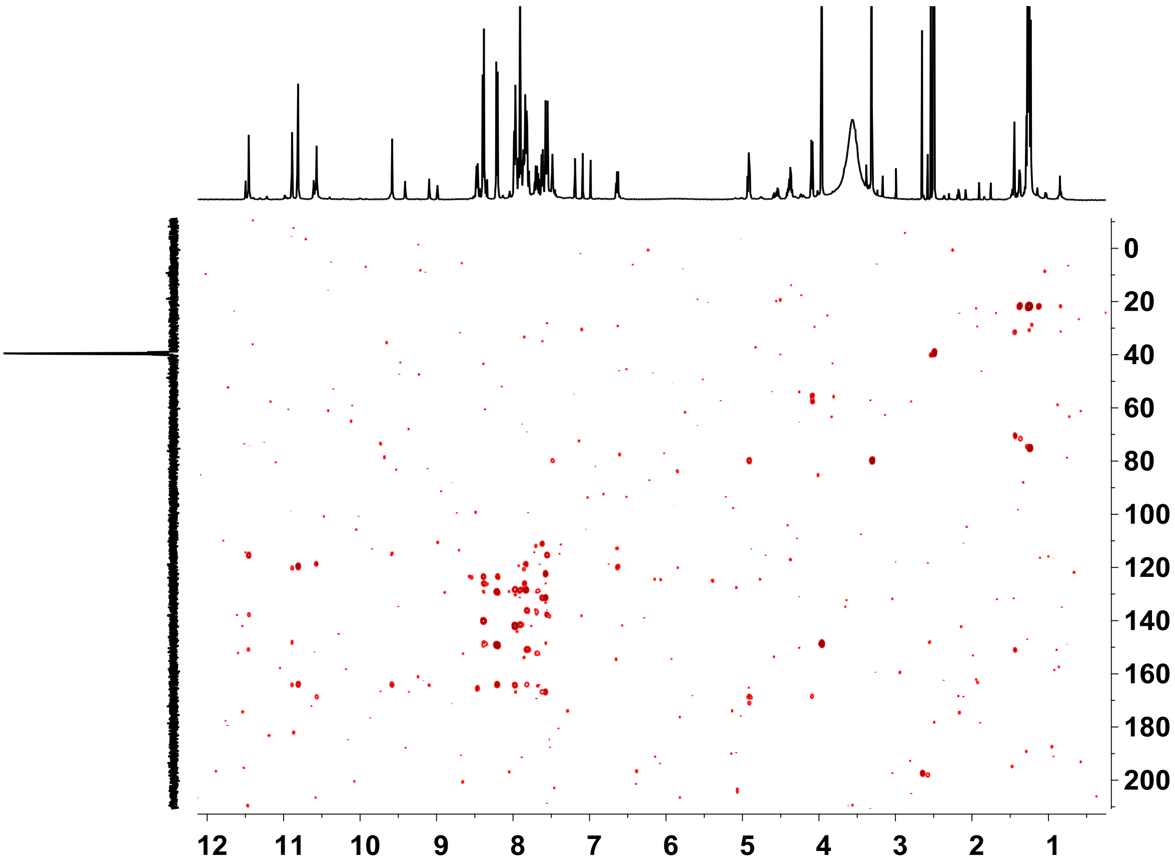

Figure S4. HMBC spectrum of cystobactamid 891-2 (**8**) measured in DMSO-d_6 at 500 MHz.

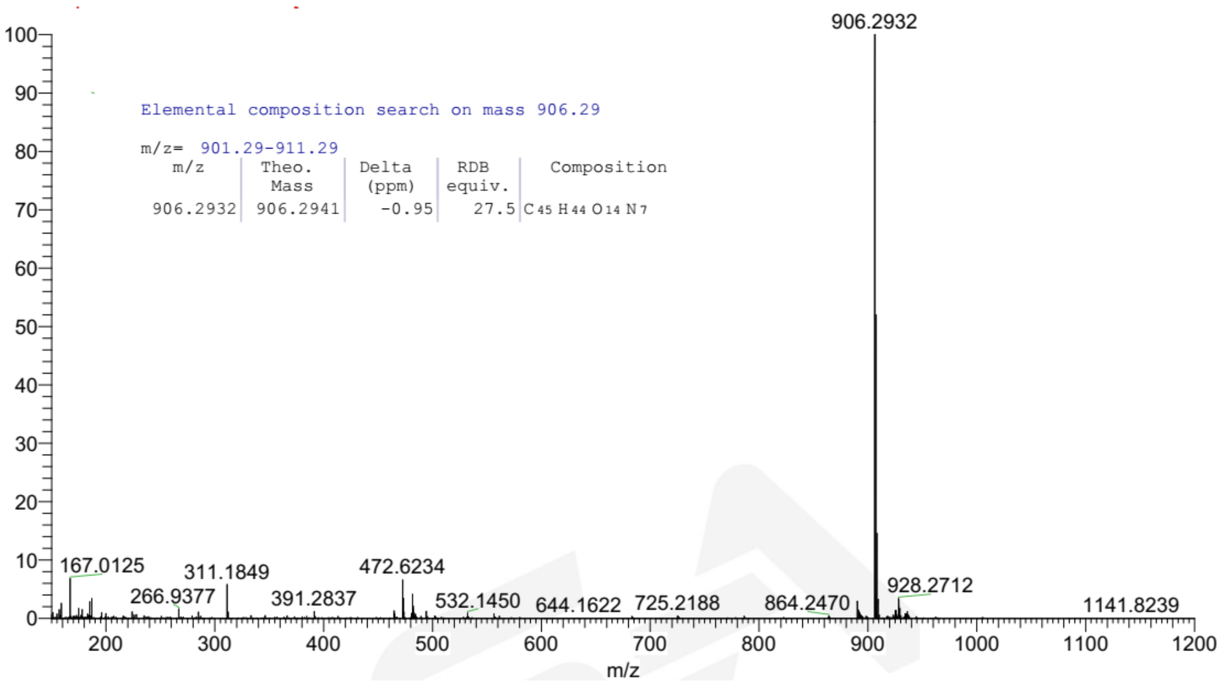

Figure S5. Positive HRESIMS spectrum of cystobactamid 905-2 (**9**).

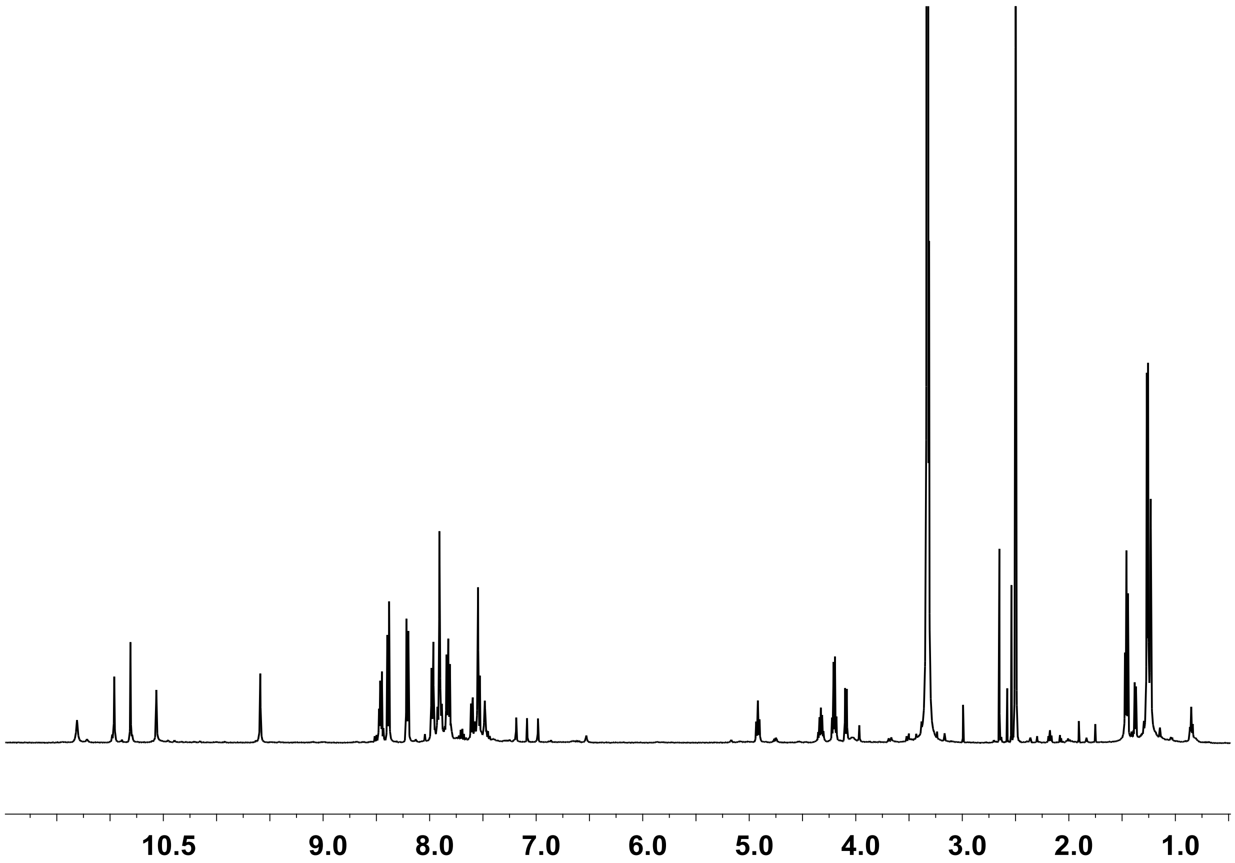

Figure S6. ^{1}H-NMR spectrum of cystobactamid 905-2 (**9**) measured in DMSO-d_6 at 500 MHz.

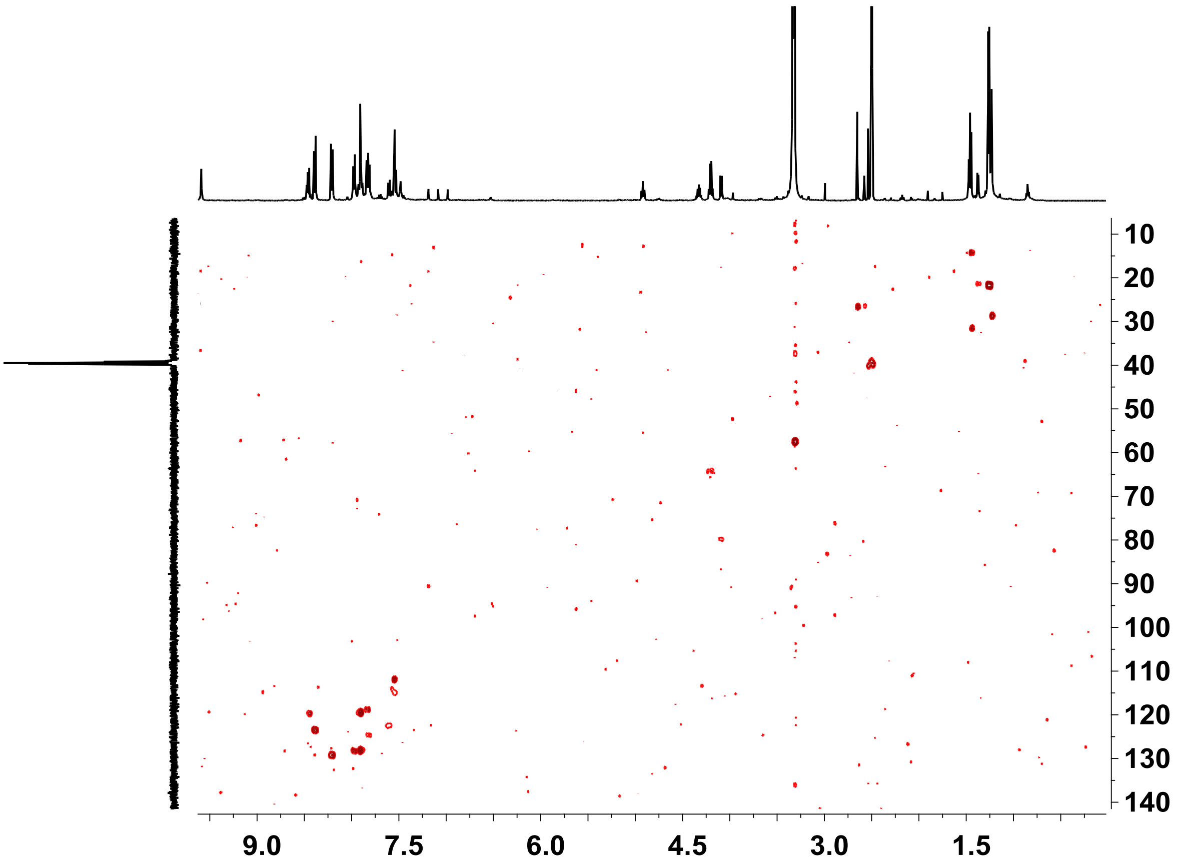

Figure S7. HMQC spectrum of cystobactamid 905-2 (**9**) measured in DMSO-d_6 at 500 MHz.

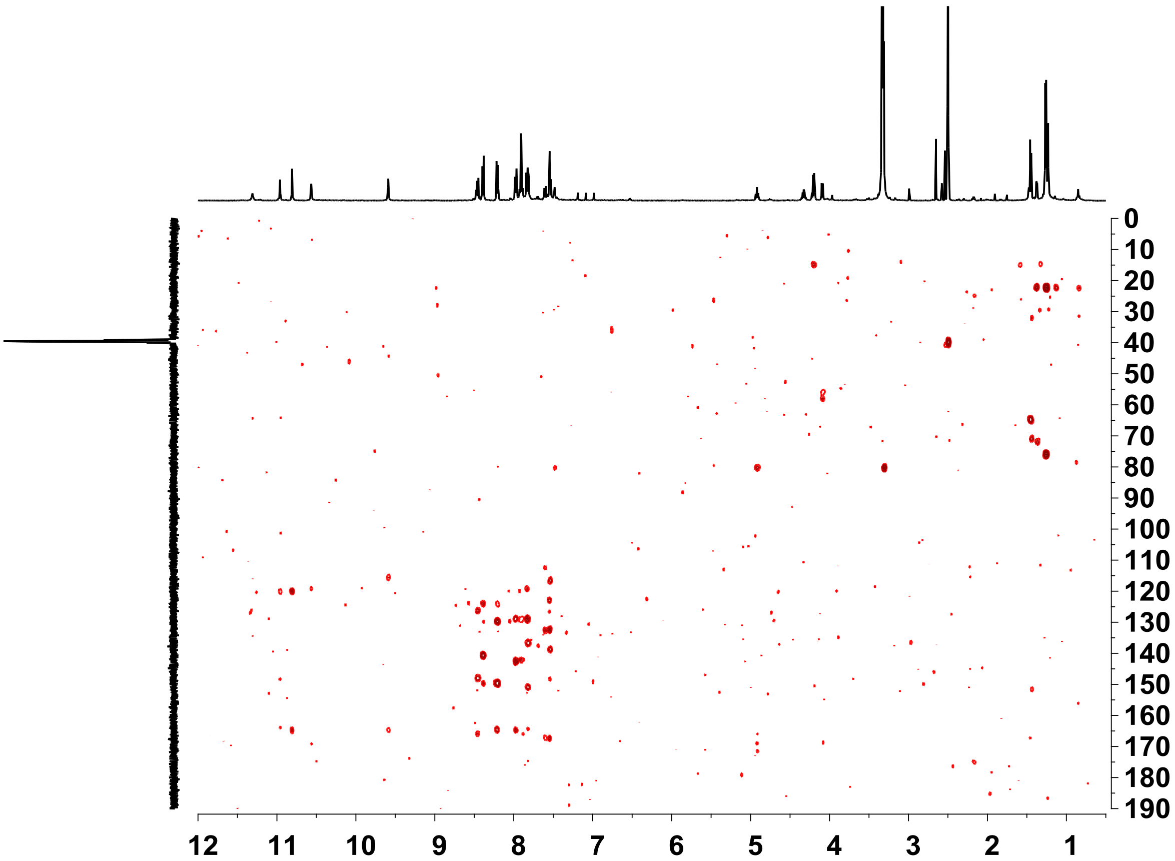

Figure S8. HMBC spectrum of cystobactamid 905-2 (**9**) measured in DMSO-d_6 at 500 MHz.

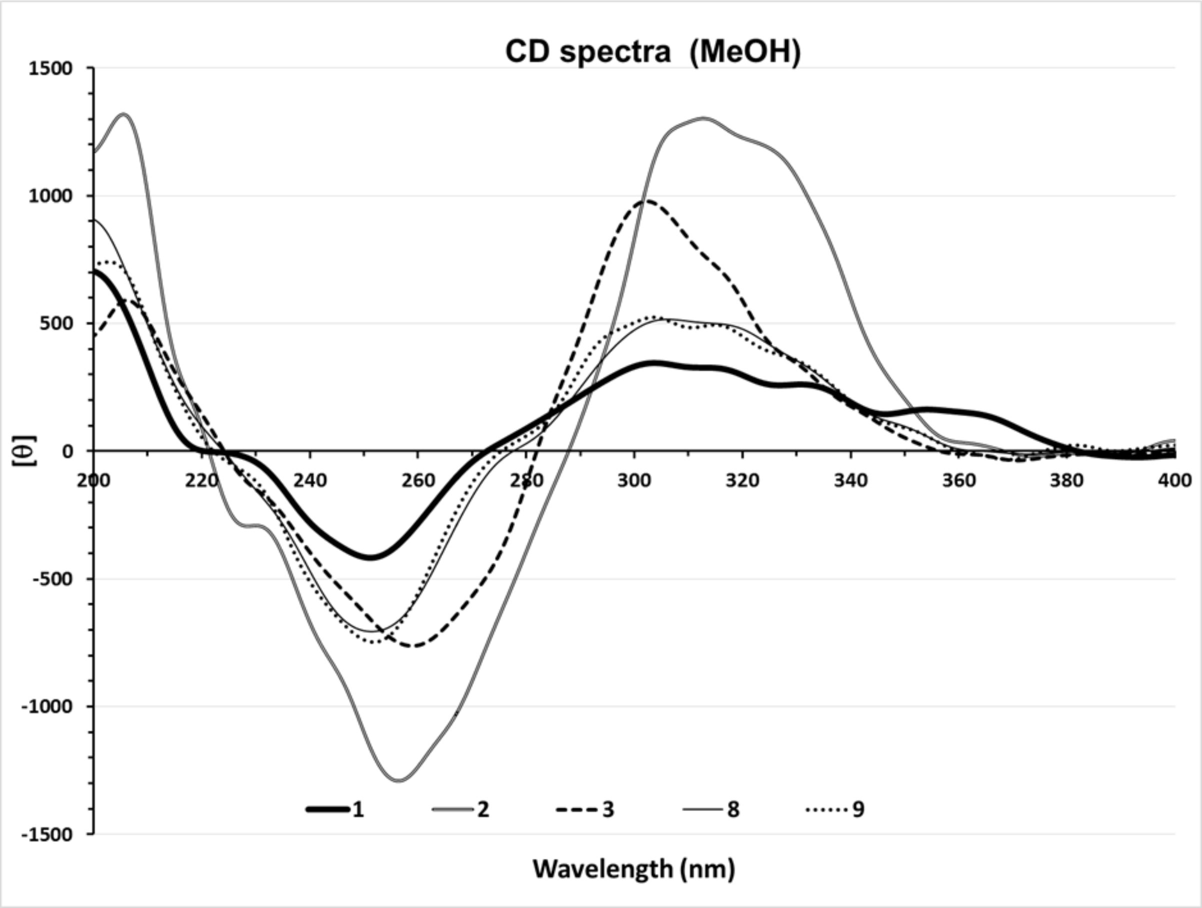

Figure S9. CD spectra of coralmycins C (**1**), D (**2**), and E (**3**), and cystobactamids 891-2 (**8**) and 905-2 (**9**).

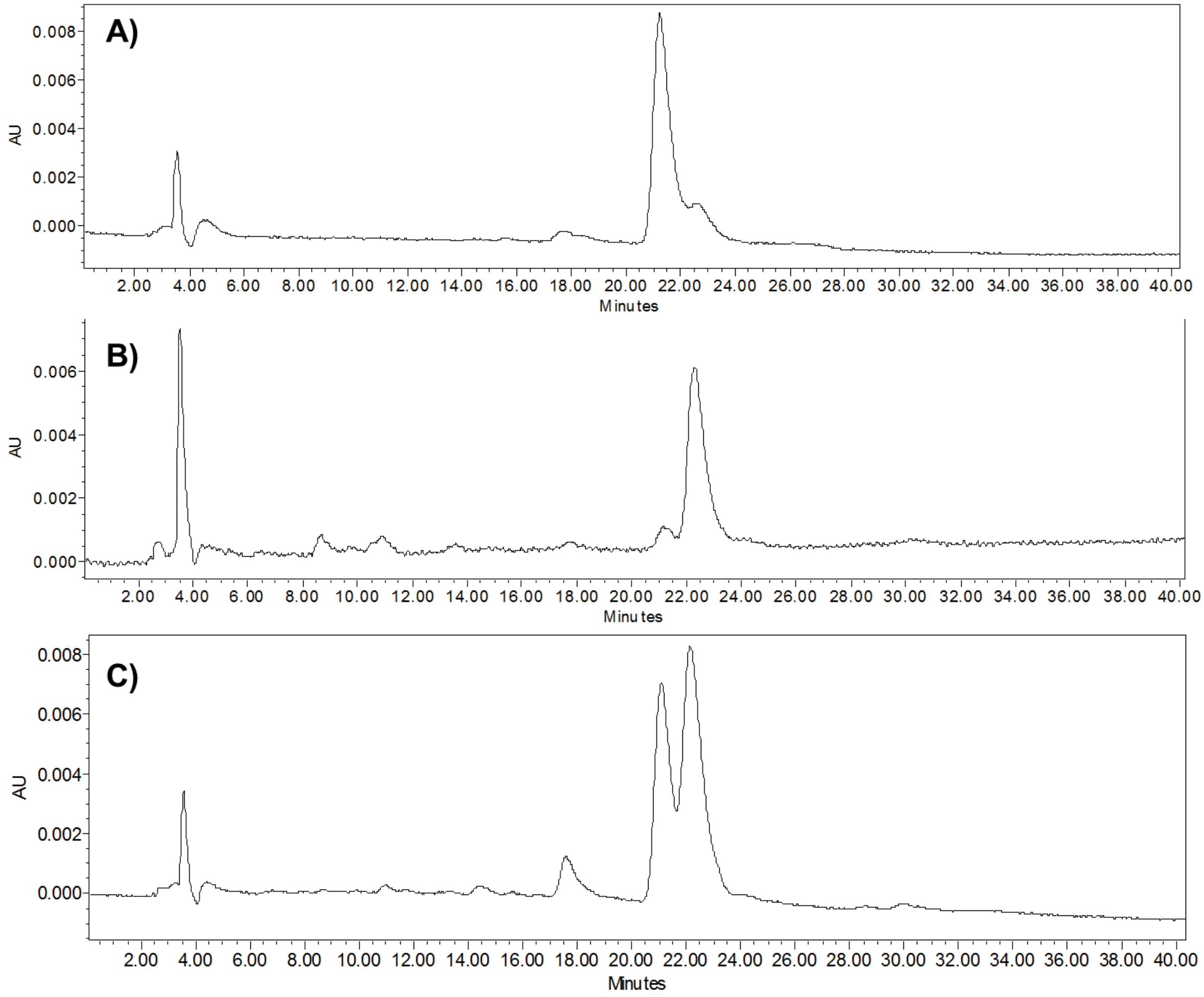

Figure S10. HPLC profiles of coralmycins C (**1**) and B (**13**)**.** HPLC condition: column YMC C18, S-4 μm, 4.6 x 250 mm; eluent: isocratic 50% CH_3CN in H_2O (v/v), containing 0.01% TFA at flow rate of 0.8 mL/min with PDA detector Waters 996. A) compound **1**; B) compound **13**; C) mixture of **1** and **13** at ratio of 1:1.

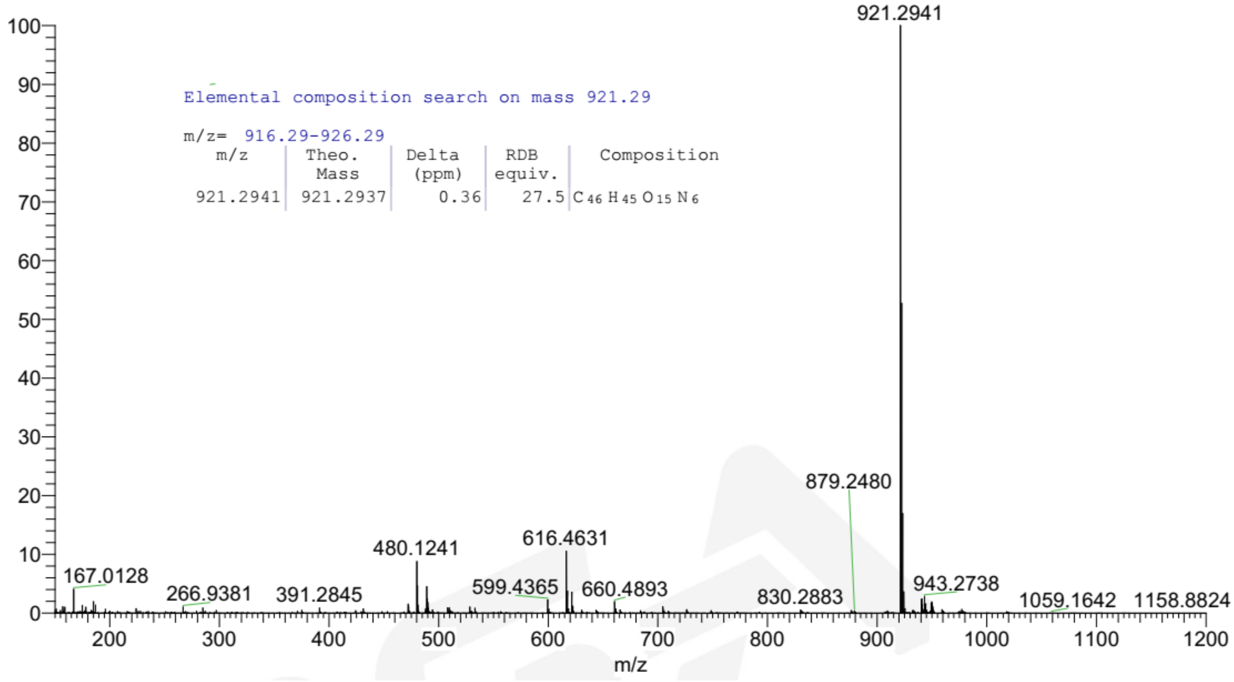

Figure S11. Positive HRESIMS spectrum of coralmycin C (**1**).

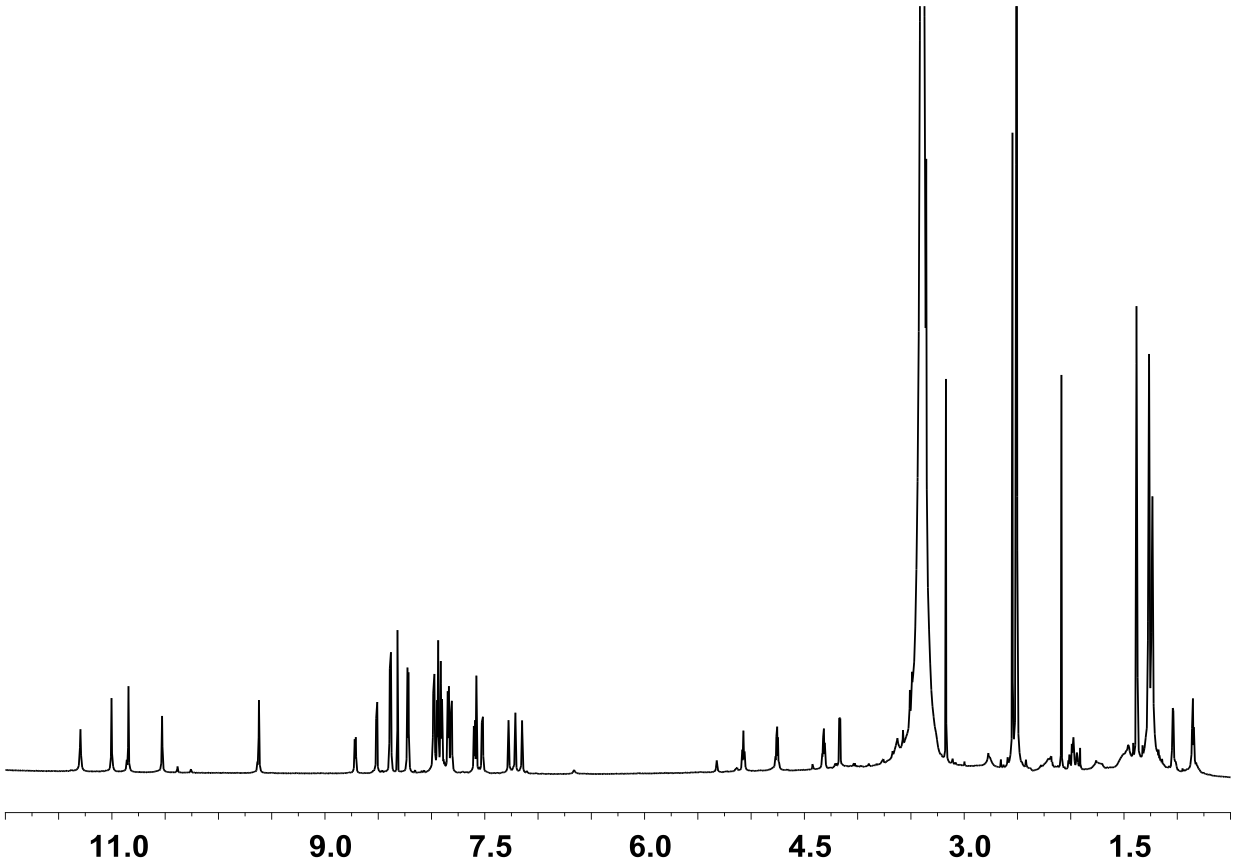

Figure S12. ^{1}H-NMR spectrum of coralmycin C (**1**) measured in DMSO-d_6 at 500 MHz.

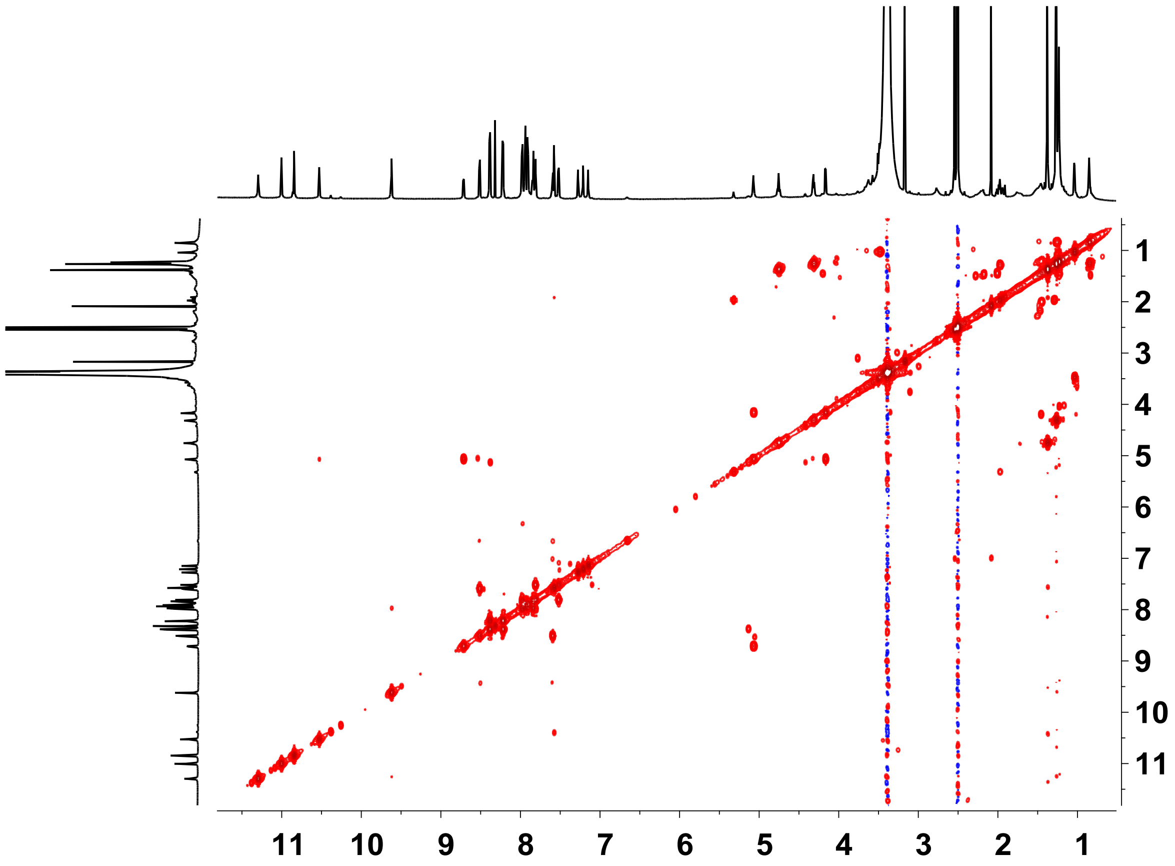

Figure S13. COSY spectrum of coralmycin C (**1**) measured in DMSO-d_6 at 500 MHz.

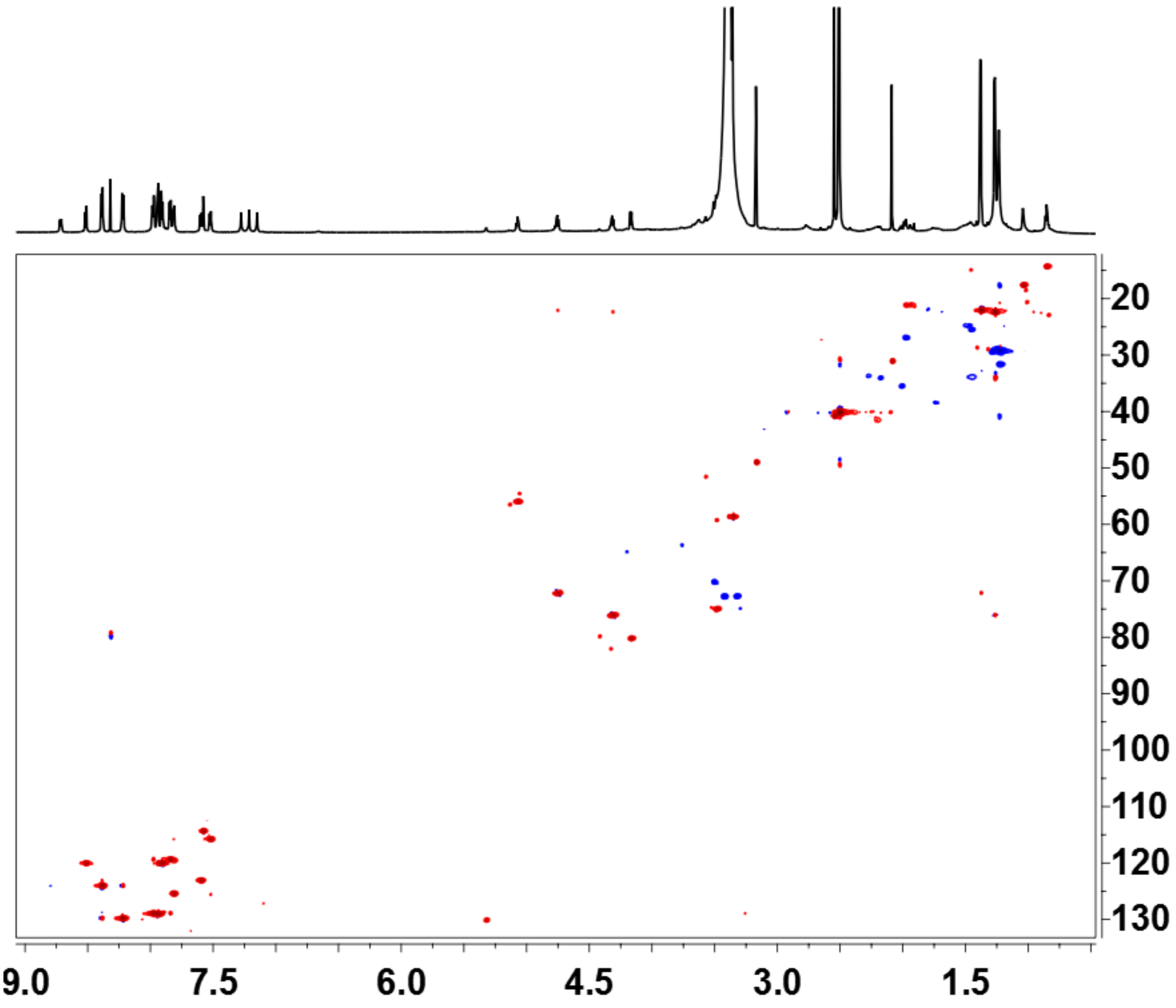

Figure S14. HMQC spectrum of coralmycin C (**1**) measured in DMSO-d_6 at 500 MHz.

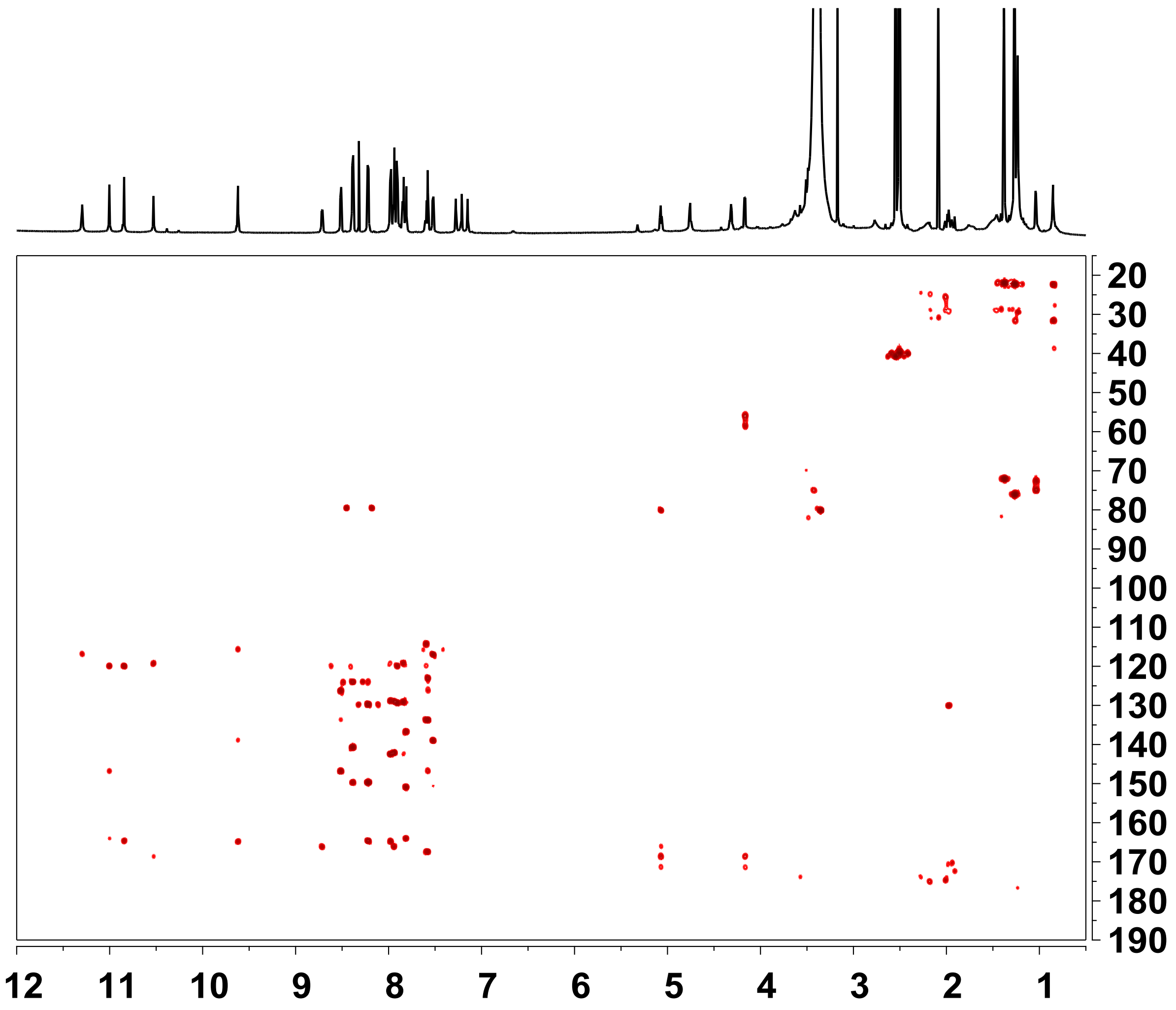

Figure S15. HMBC spectrum of coralmycin C (**1**) measured in DMSO-d_6 at 500 MHz.

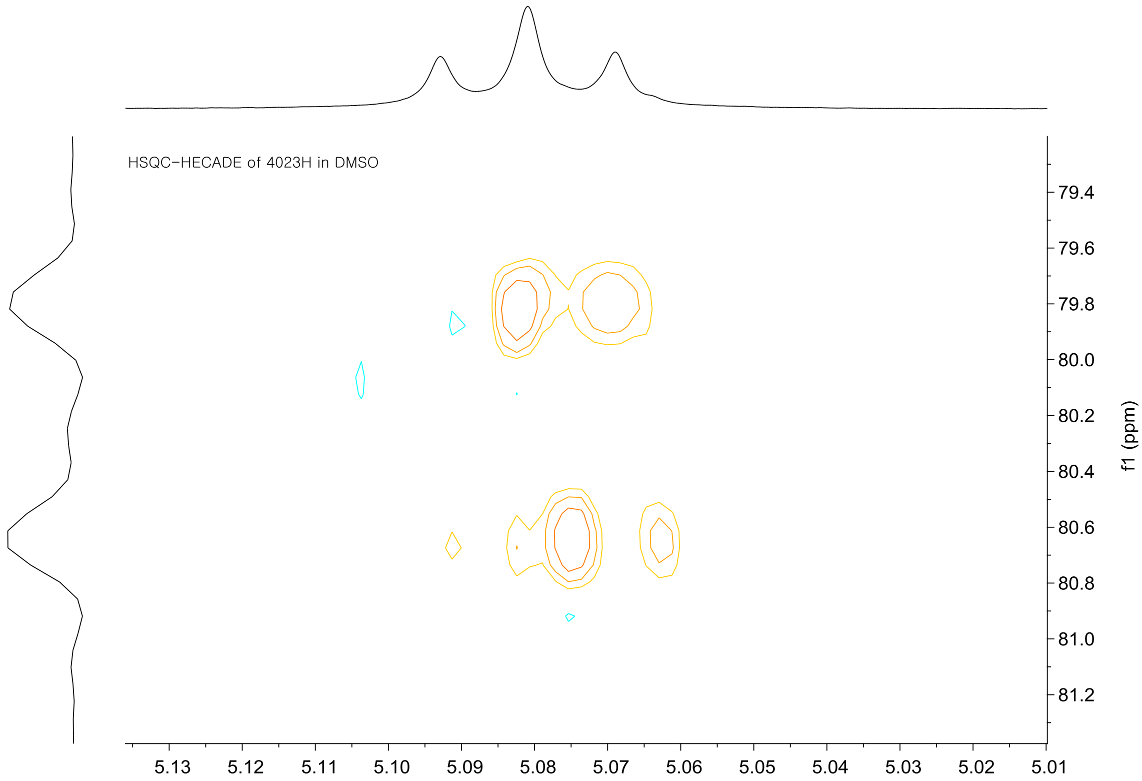

Figure S16. HECADE spectrum of coralmycin C (**1**) measured in DMSO-d_6 at 700 MHz.

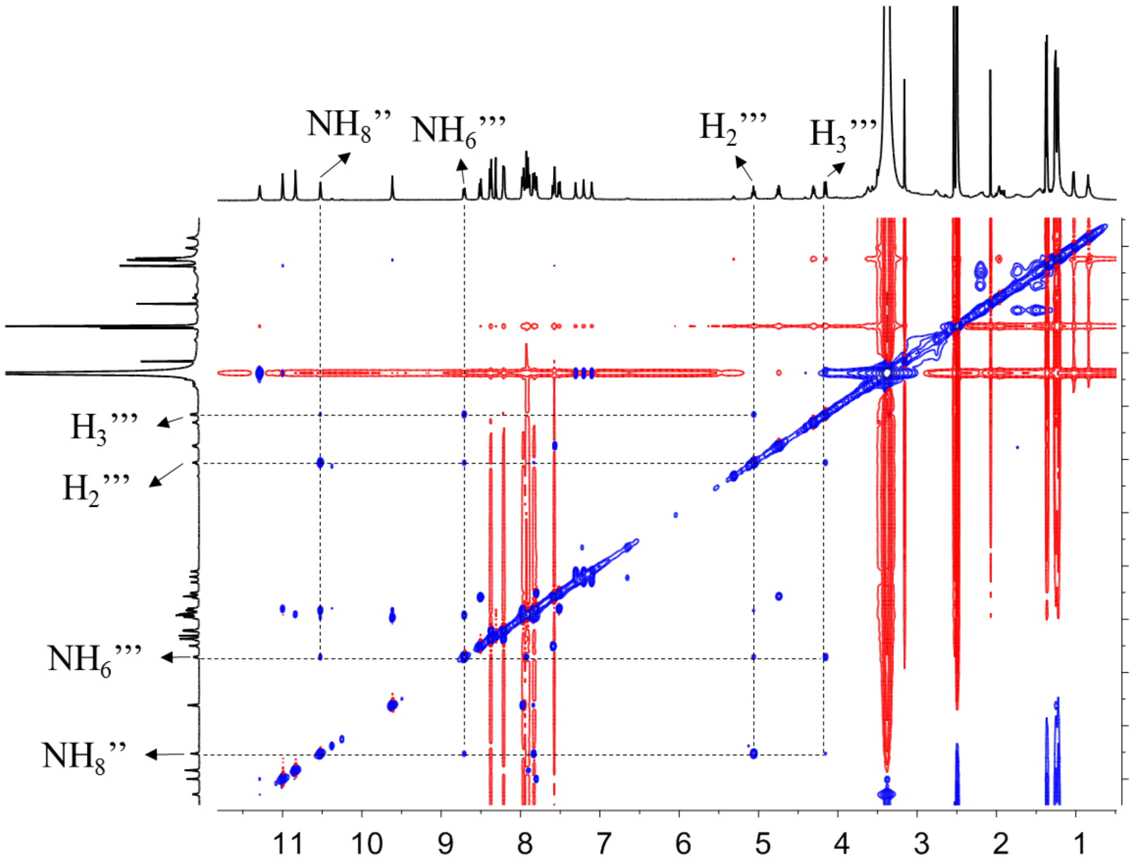

Figure S17. NOESY spectrum of coralmycin C (**1**) measured in DMSO-d$_6$ at 500 MHz.

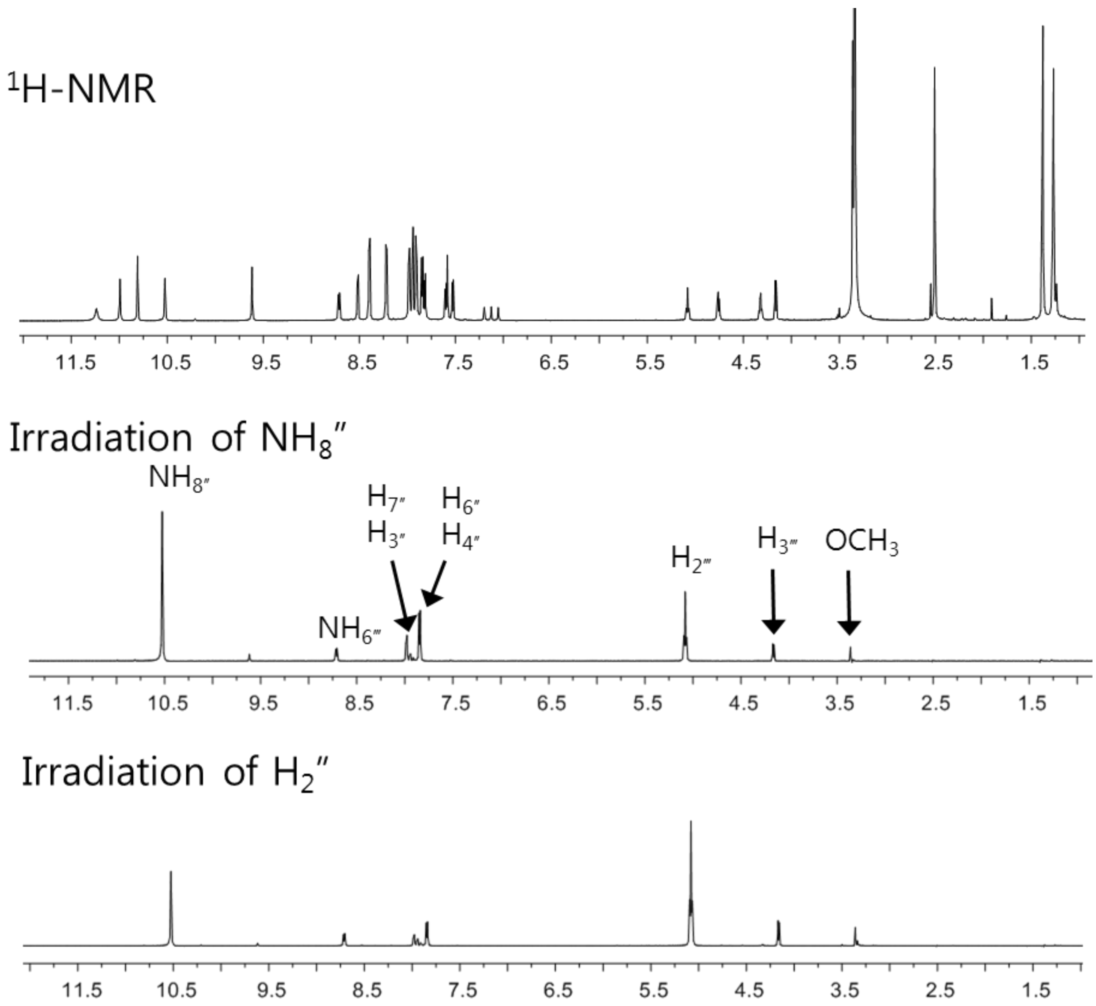

Figure S18. The NOE differential spectra of coralmycin C (**1**) measured in DMSO-d_6 at 700 MHz.

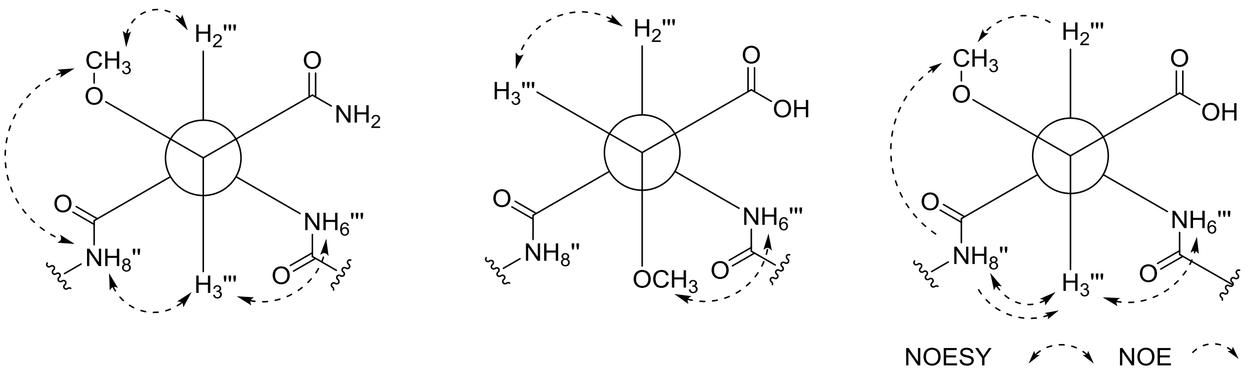

11 **13** **1**

Figure S19. NOE correlations of coralmycin C (**1**) in comparison with cystobactamid 919-2 (**11**) and coralmycin B (**13**).

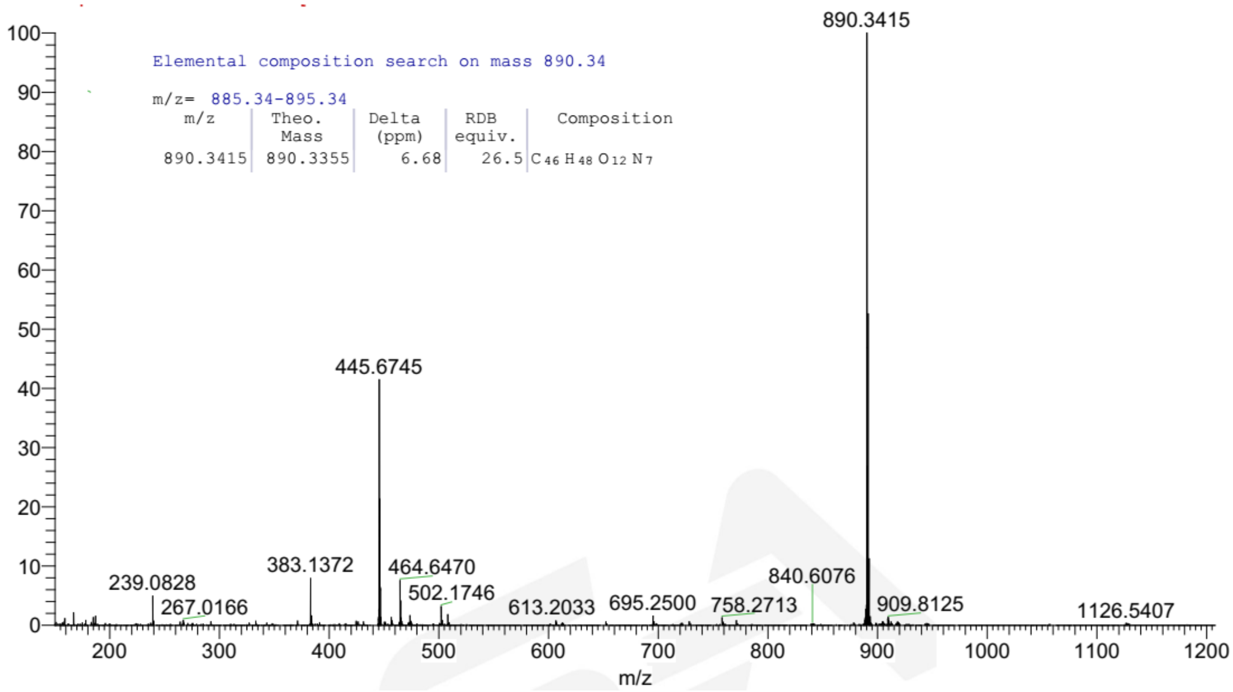

Figure S20. Positive HRESIMS spectrum of coralmycin D (**2**).

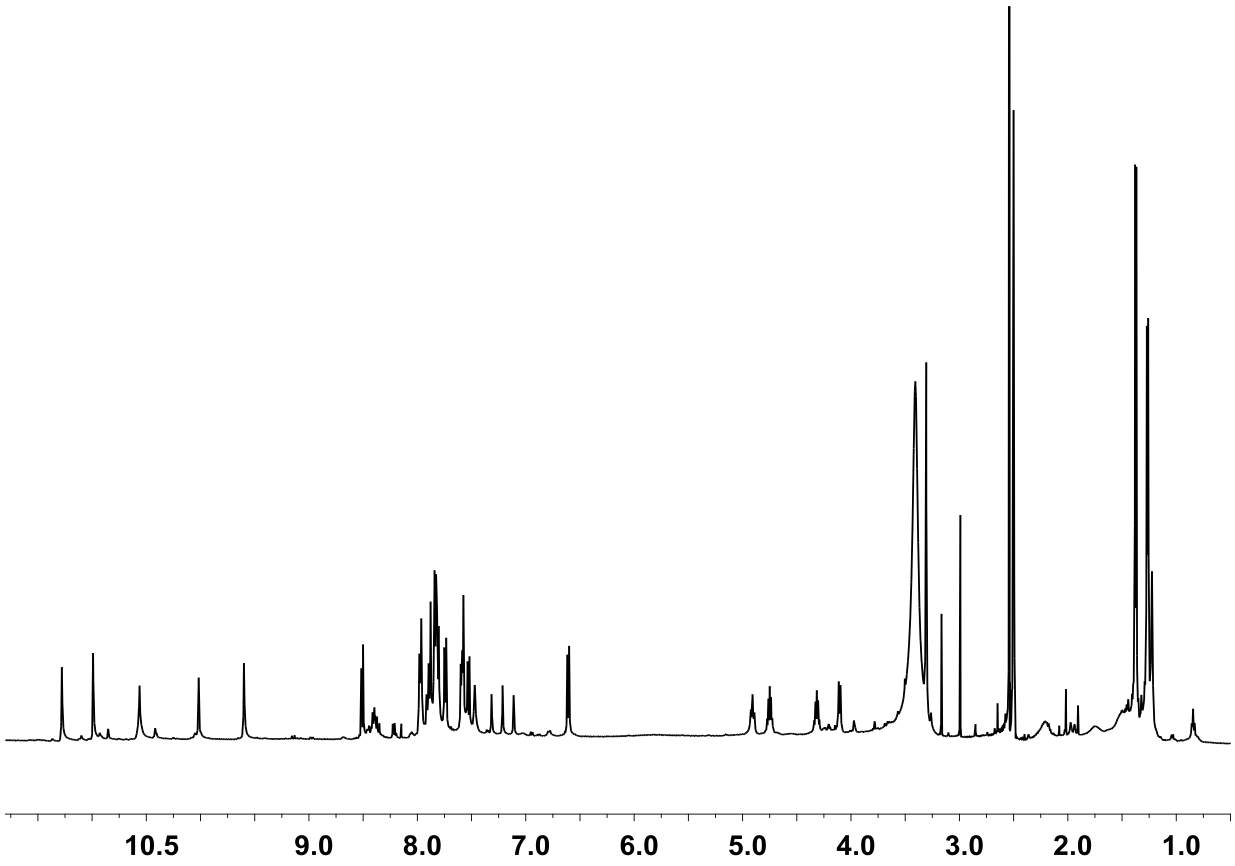

Figure S21. ^{1}H-NMR spectrum of coralmycin D (**2**) measured in DMSO-d_6 at 500 MHz.

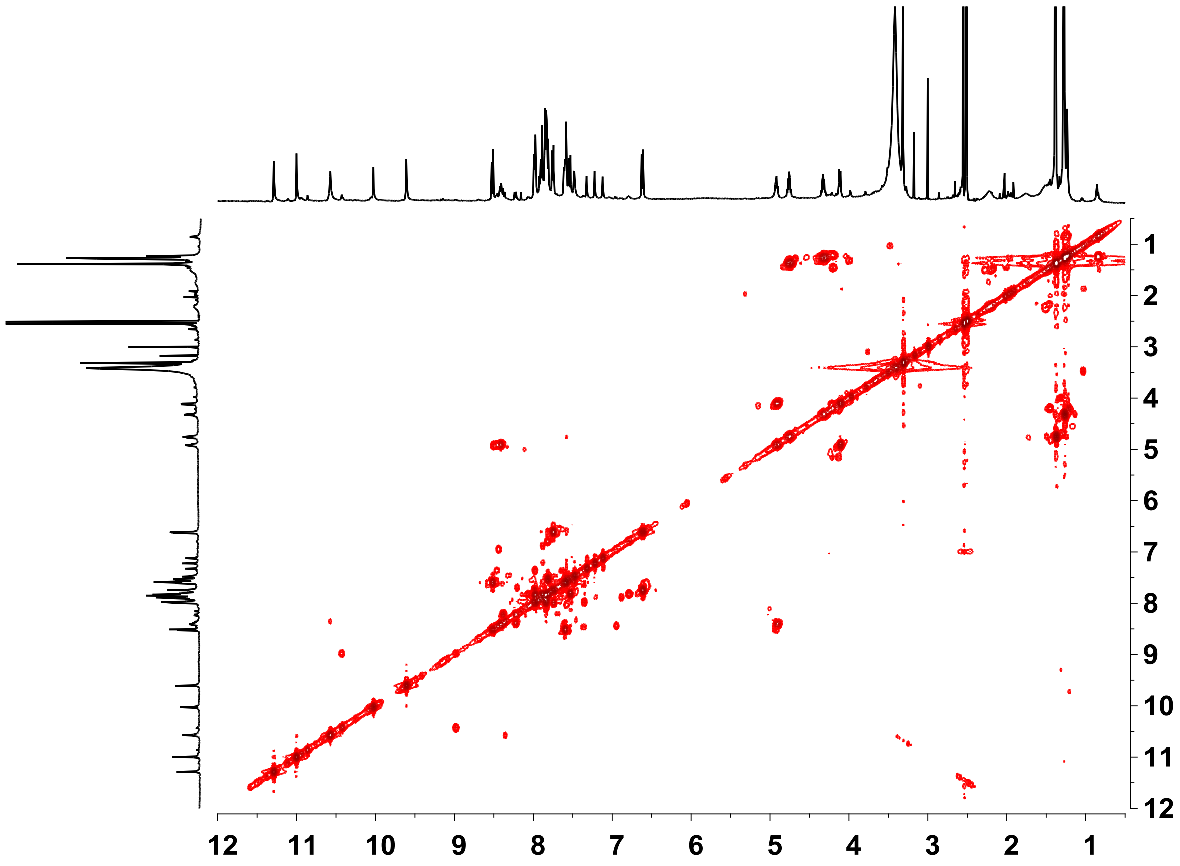

Figure S22. COSY spectrum of coralmycin D (**2**) measured in DMSO-d_6 at 500 MHz.

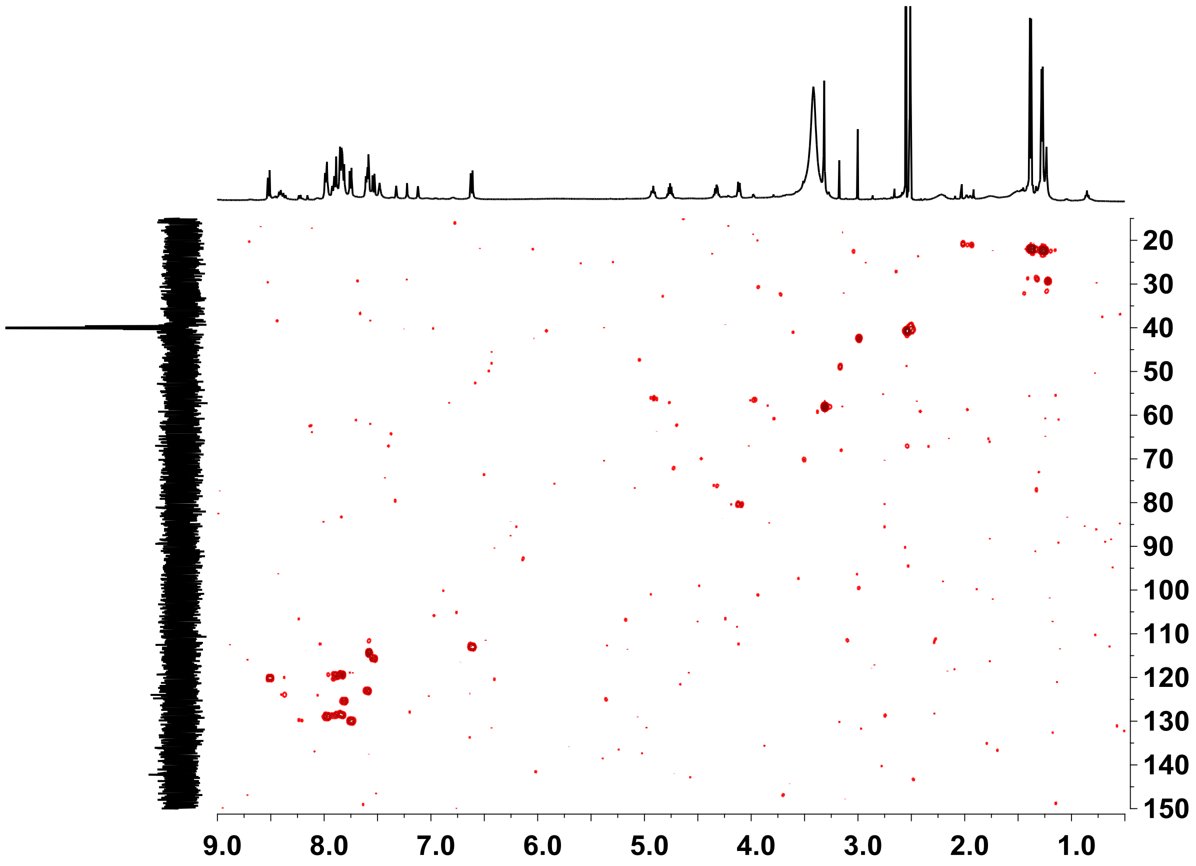

Figure S23. HMQC spectrum of coralmycin D (**2**) measured in DMSO-d_6 at 500 MHz.

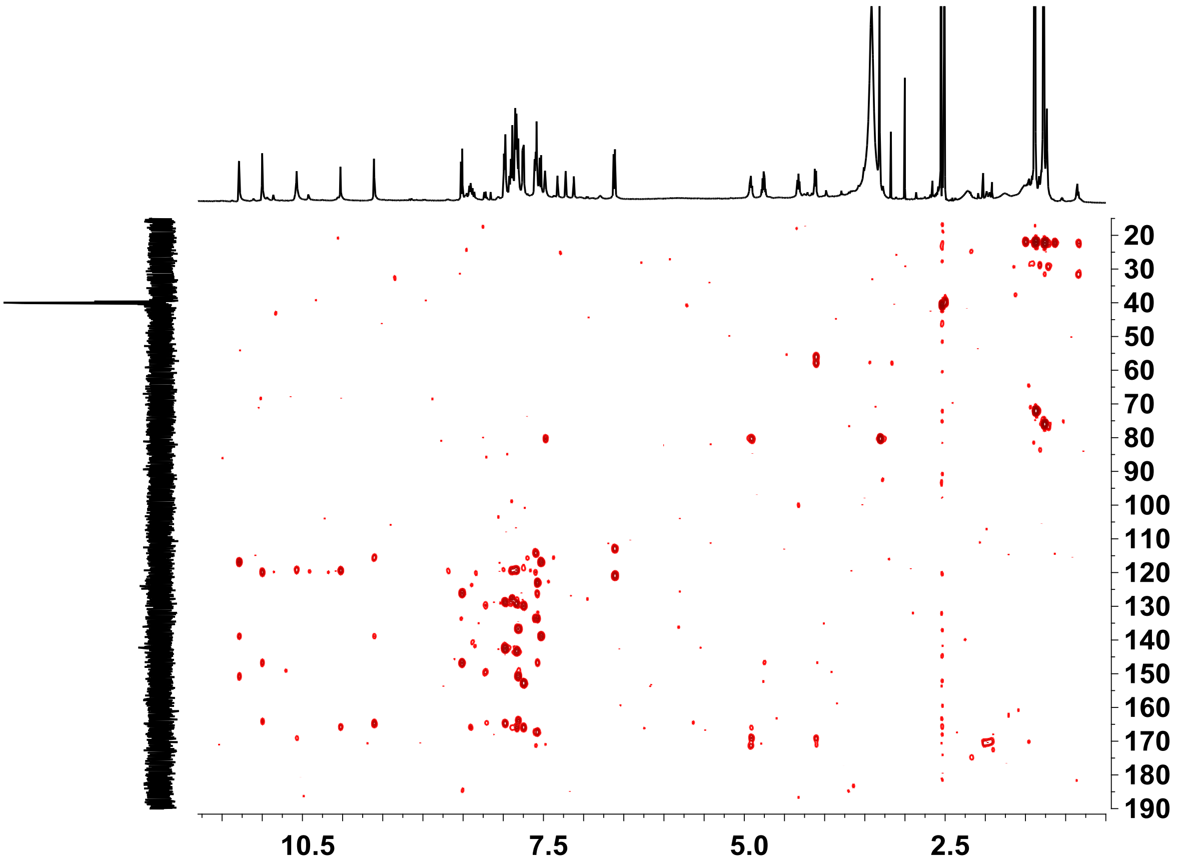

Figure S24. HMBC spectrum of coralmycin D (**2**) measured in DMSO-d_6 at 500 MHz.

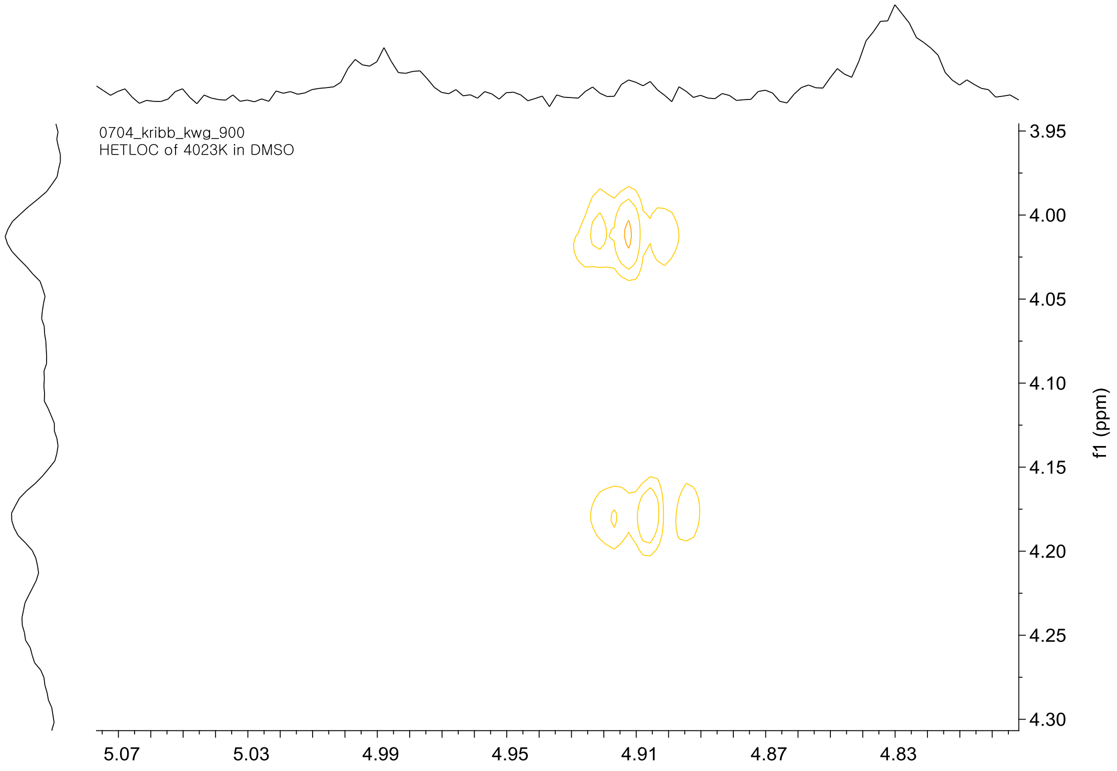

Figure S25. HETLOC spectrum of coralmycin D (**2**) measured in DMSO-d_6 at 900 MHz.

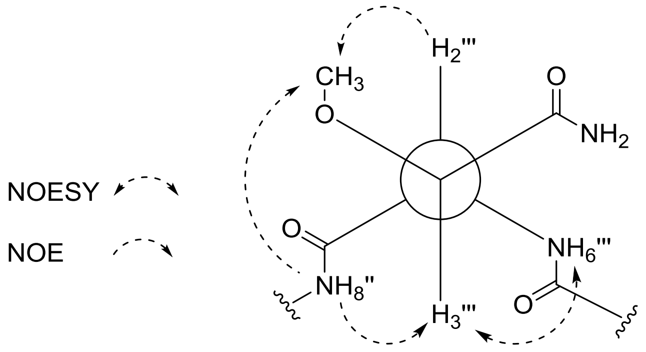

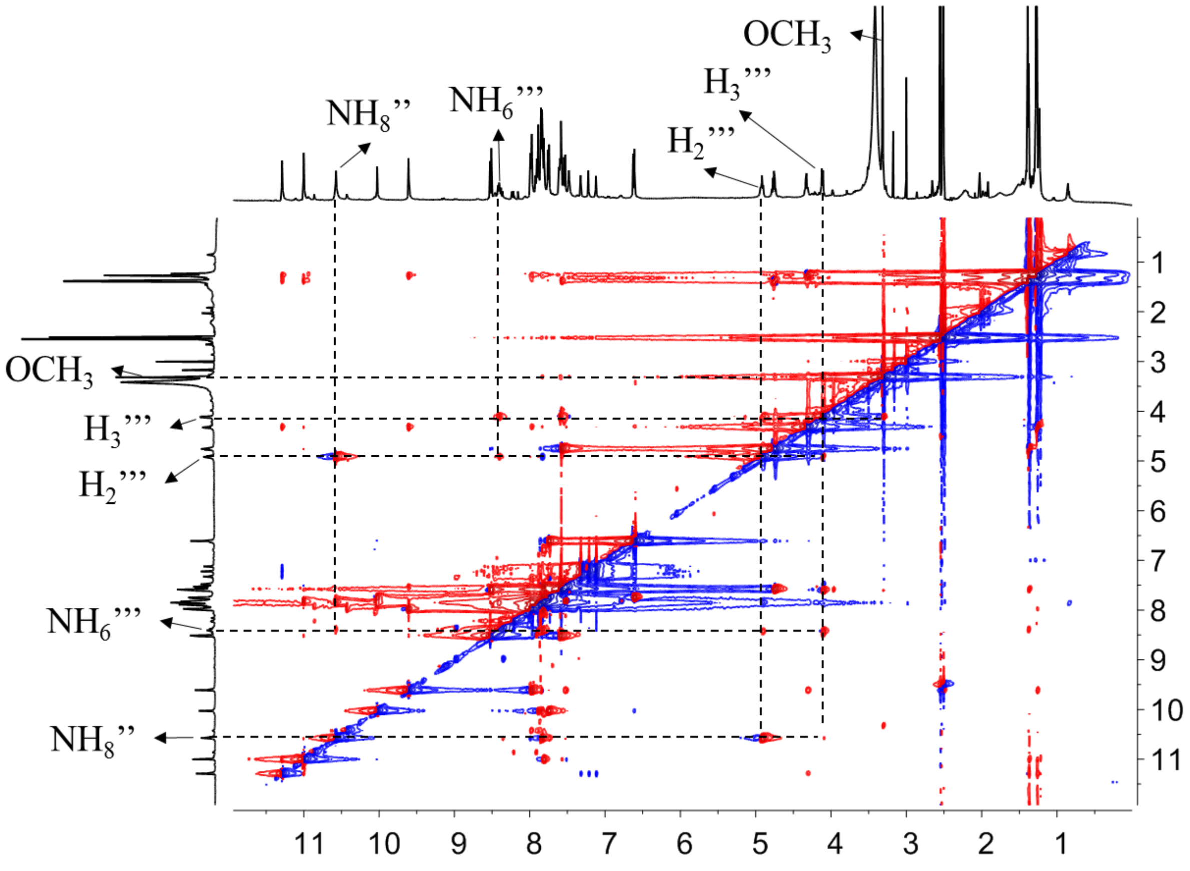

Figure S26. NOESY spectrum of coralmycin D (**2**) measured in DMSO-d_6 at 500 MHz.

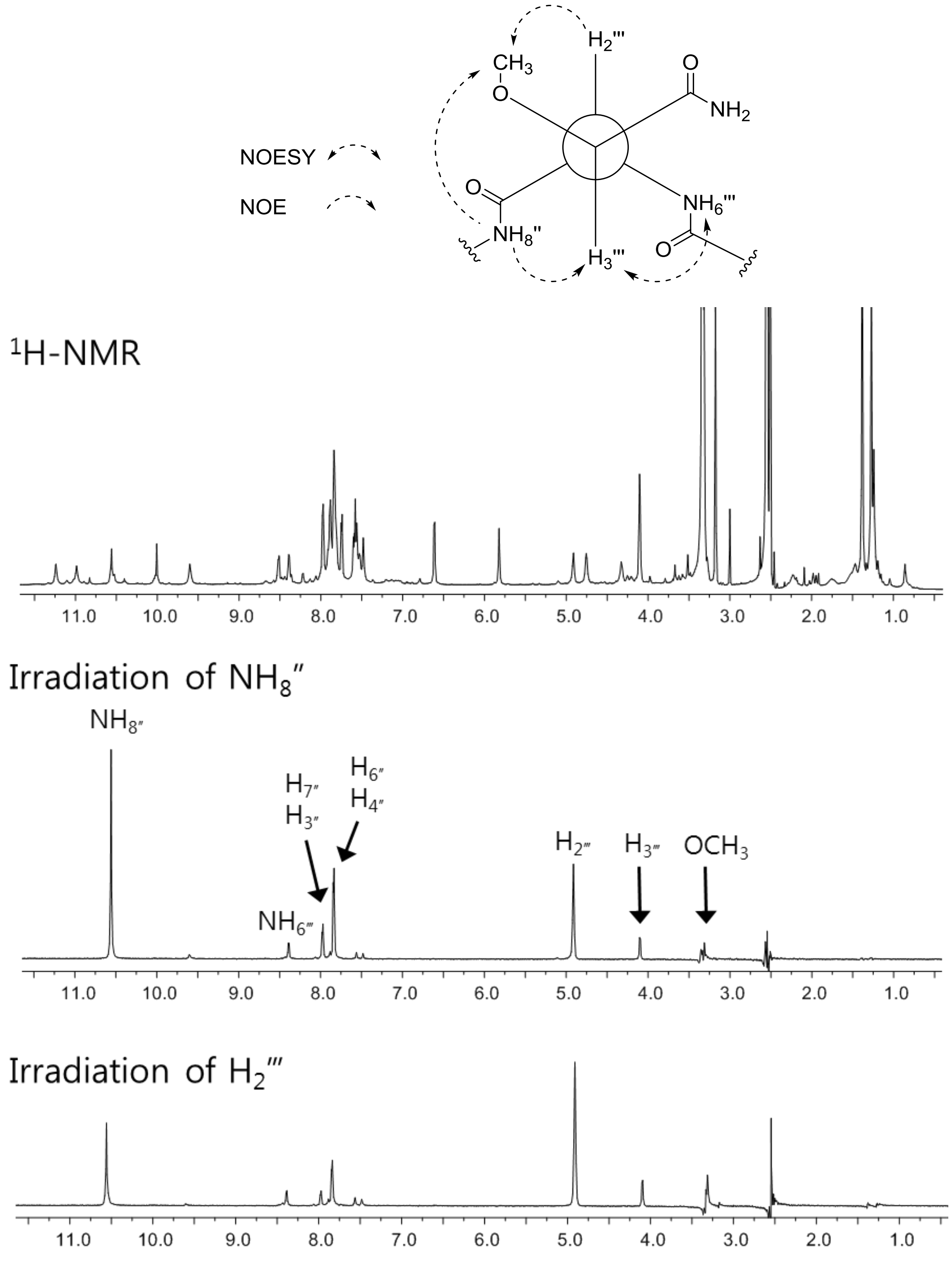

Figure S27. The NOE differential spectra of coralmycin D (**2**) measured in DMSO-d_6 at 800 MHz.

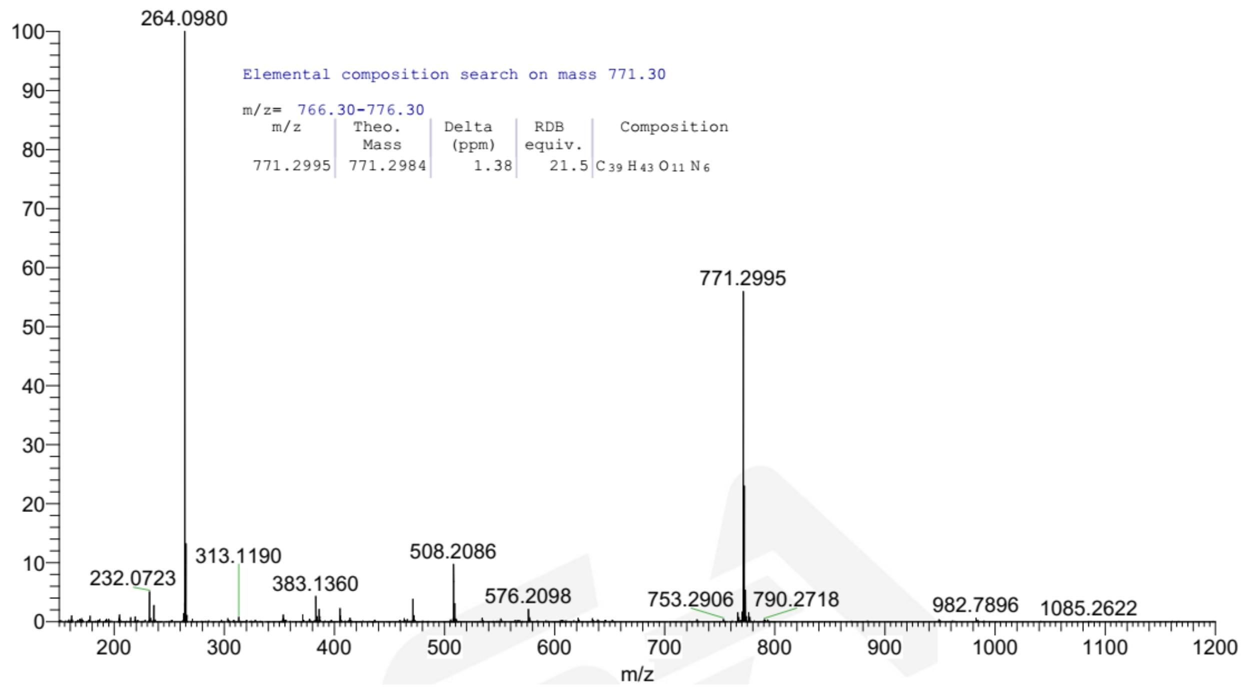

Figure S28. Positive HRESIMS spectrum of coralmycin E (**3**).

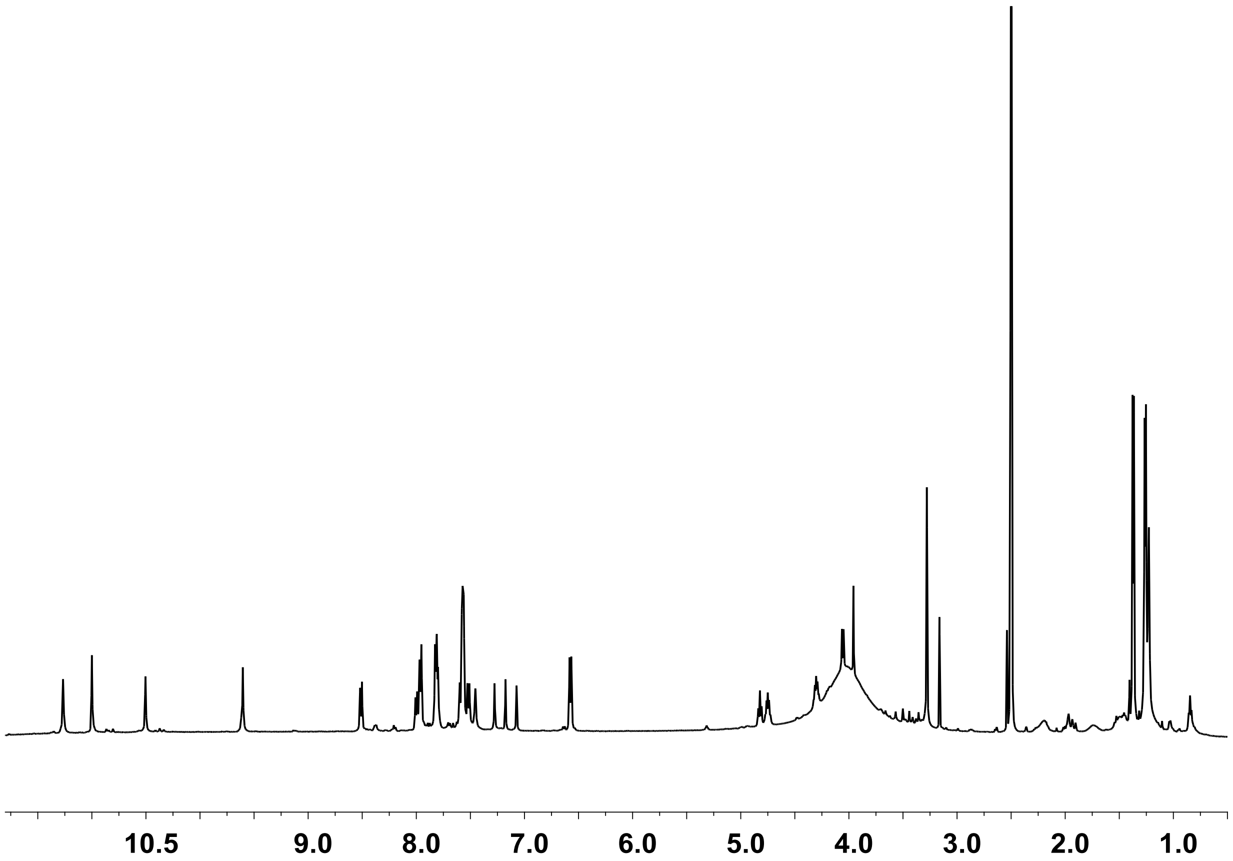

Figure S29. ^{1}H-NMR spectrum of coralmycin E (**3**) measured in DMSO-d_6 at 500 MHz.

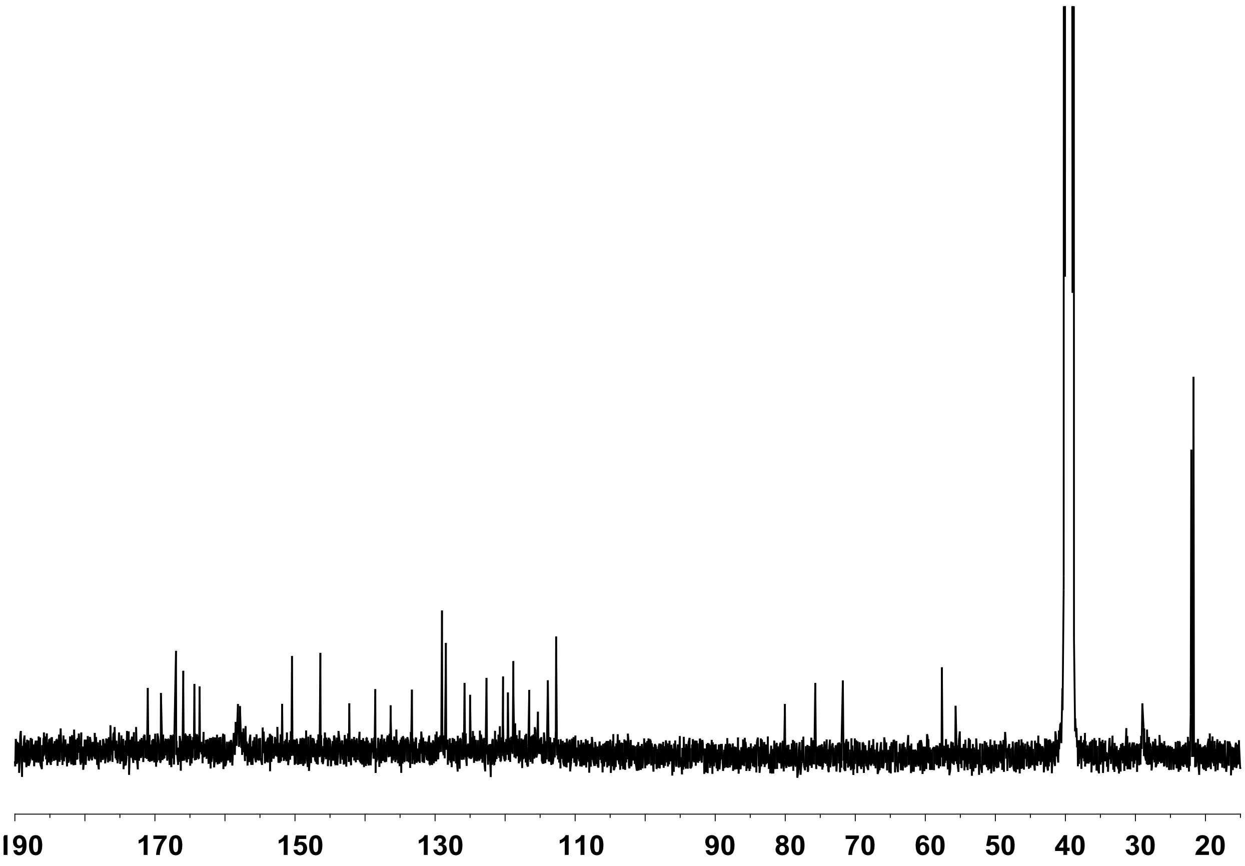

Figure S30. ^{13}C-NMR spectrum of coralmycin E (**3**) measured in DMSO-d_6 at 500 MHz.

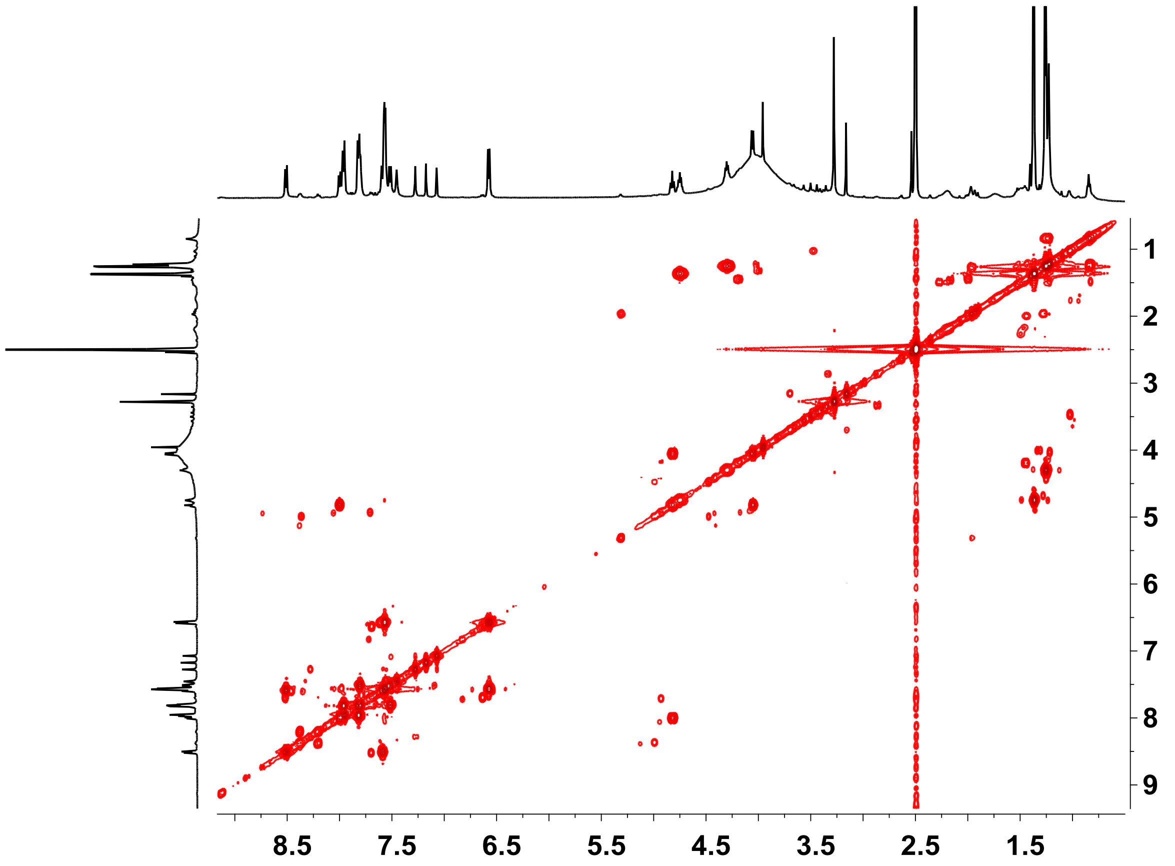

Figure S31. COSY spectrum of coralmycin E (**3**) measured in DMSO-d_6 at 500 MHz.

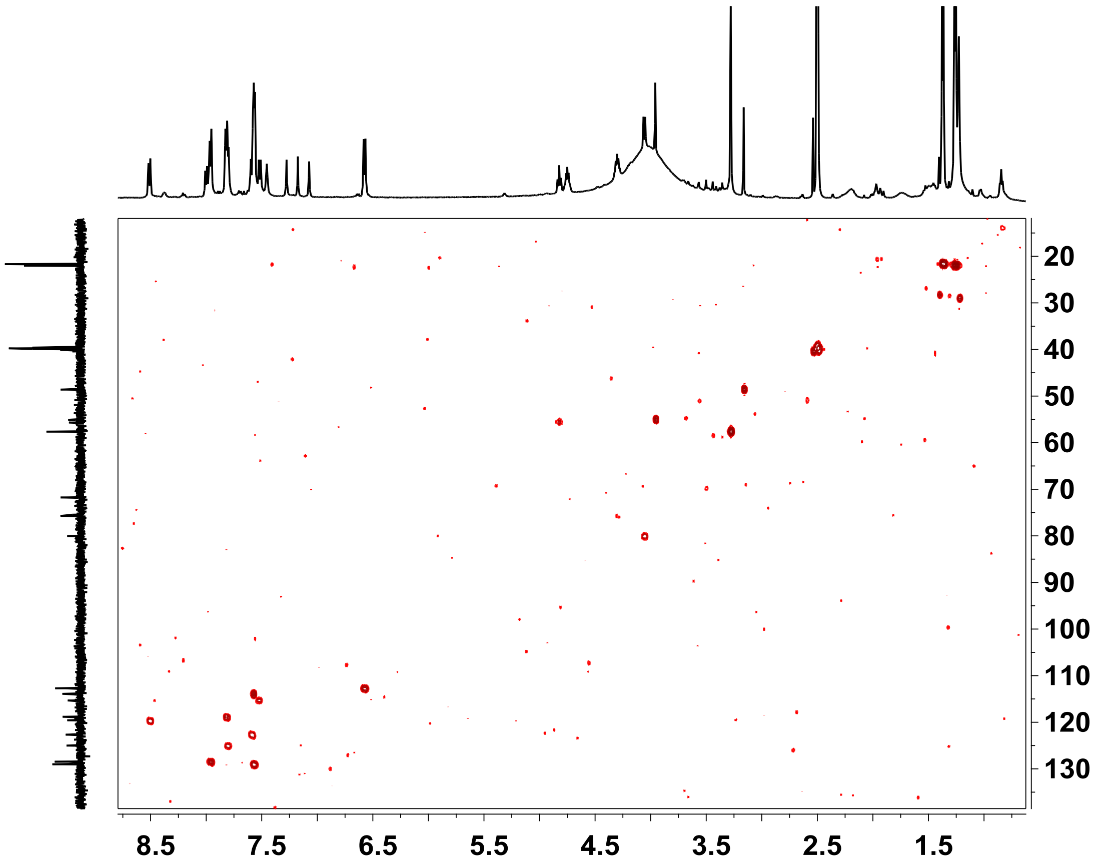

Figure S32. HMQC spectrum of coralmycin E (**3**) measured in DMSO-d_6 at 500 MHz.

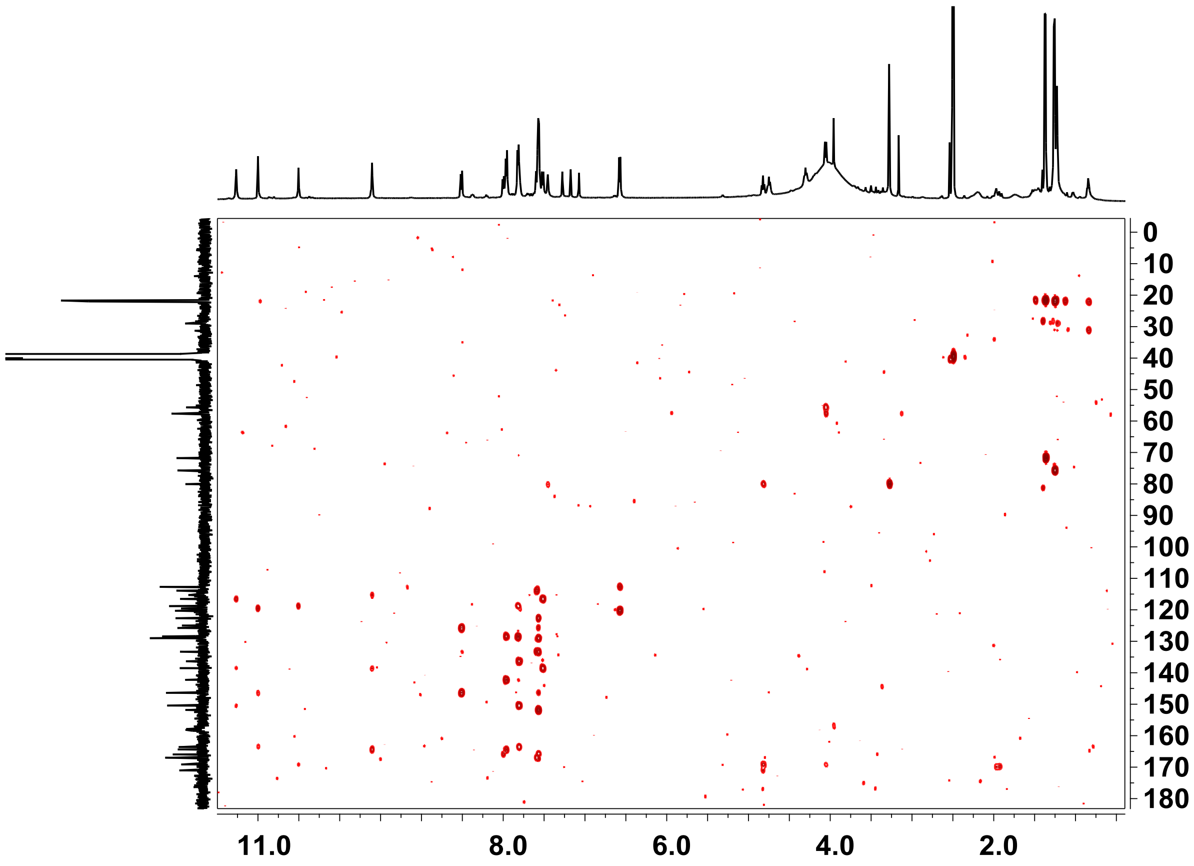

Figure S33. HMBC spectrum of coralmycin E (**3**) measured in DMSO-d_6 at 500 MHz.

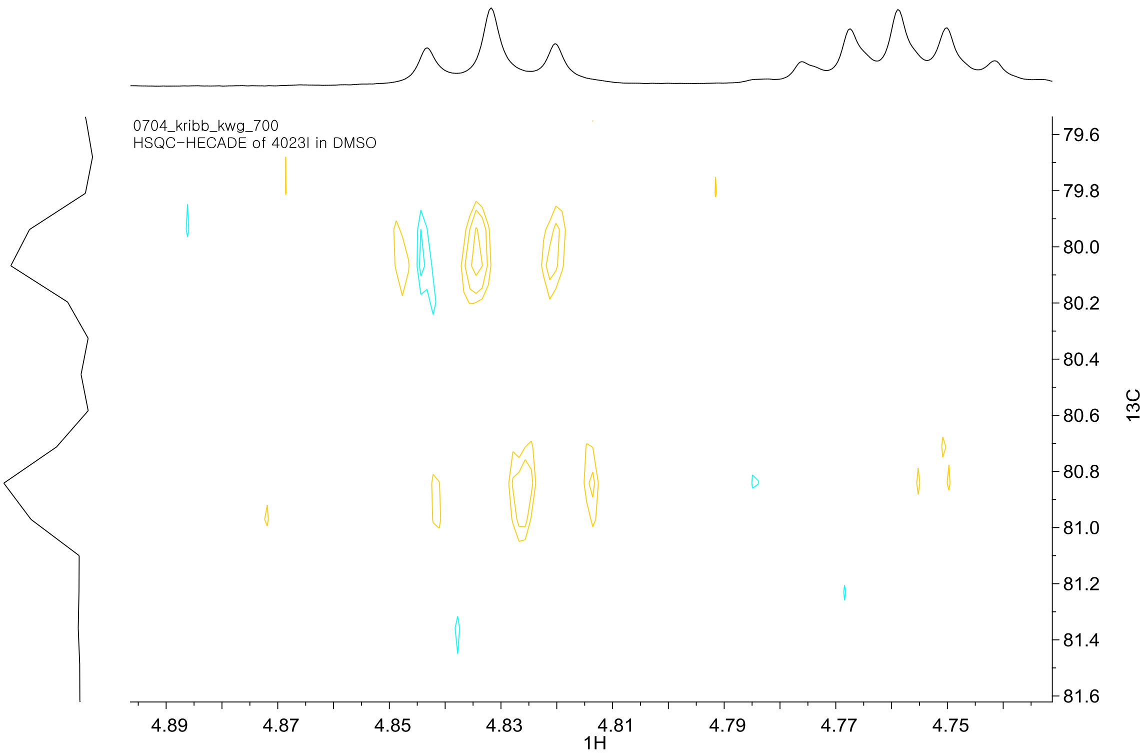

Figure S34. HECADE spectrum of coralmycin E (**3**) measured in DMSO-d_6 at 700 MHz.

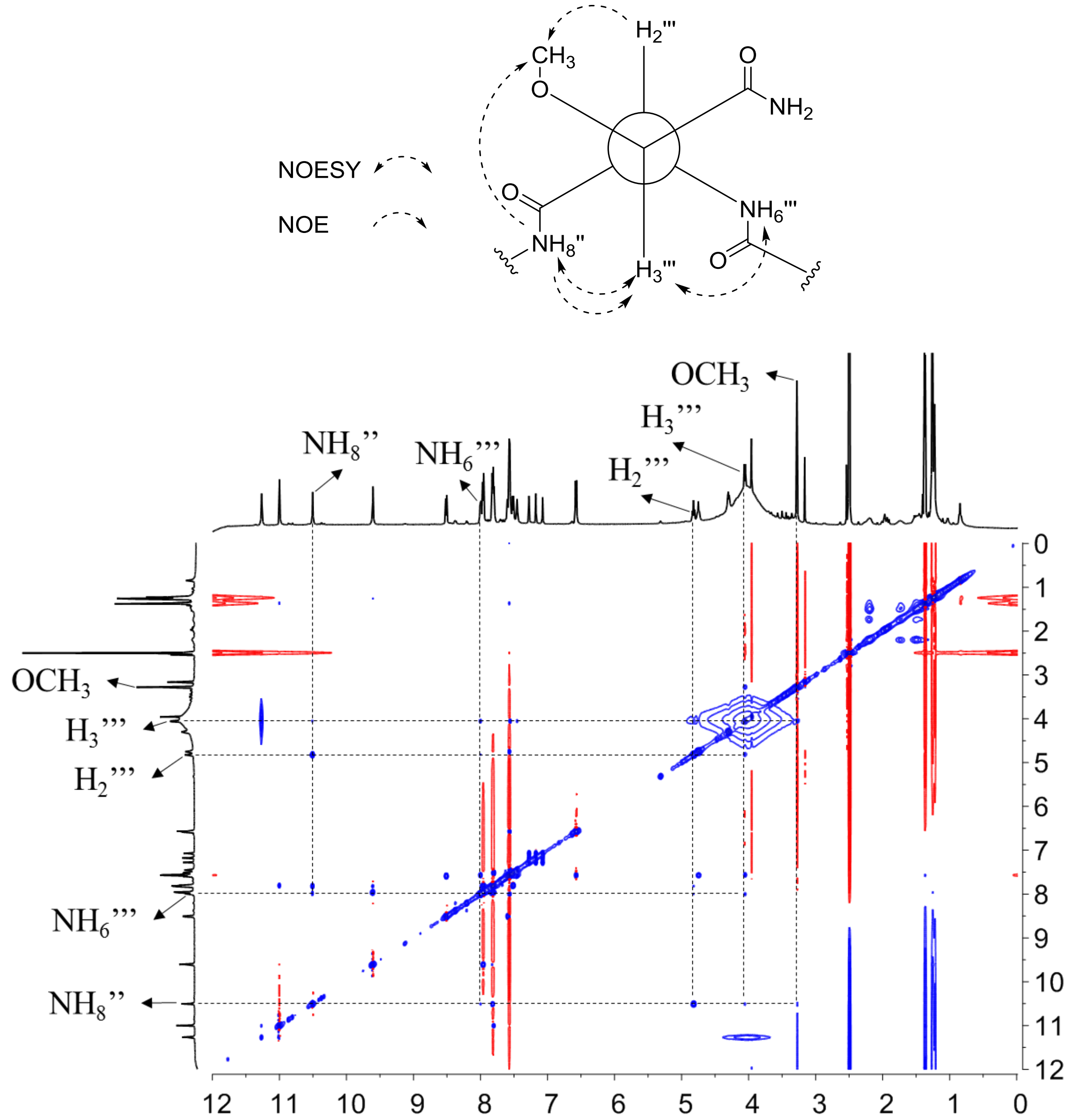

Figure S35. NOESY spectrum of coralmycin E (**3**) measured in DMSO-d_6 at 500 MHz.

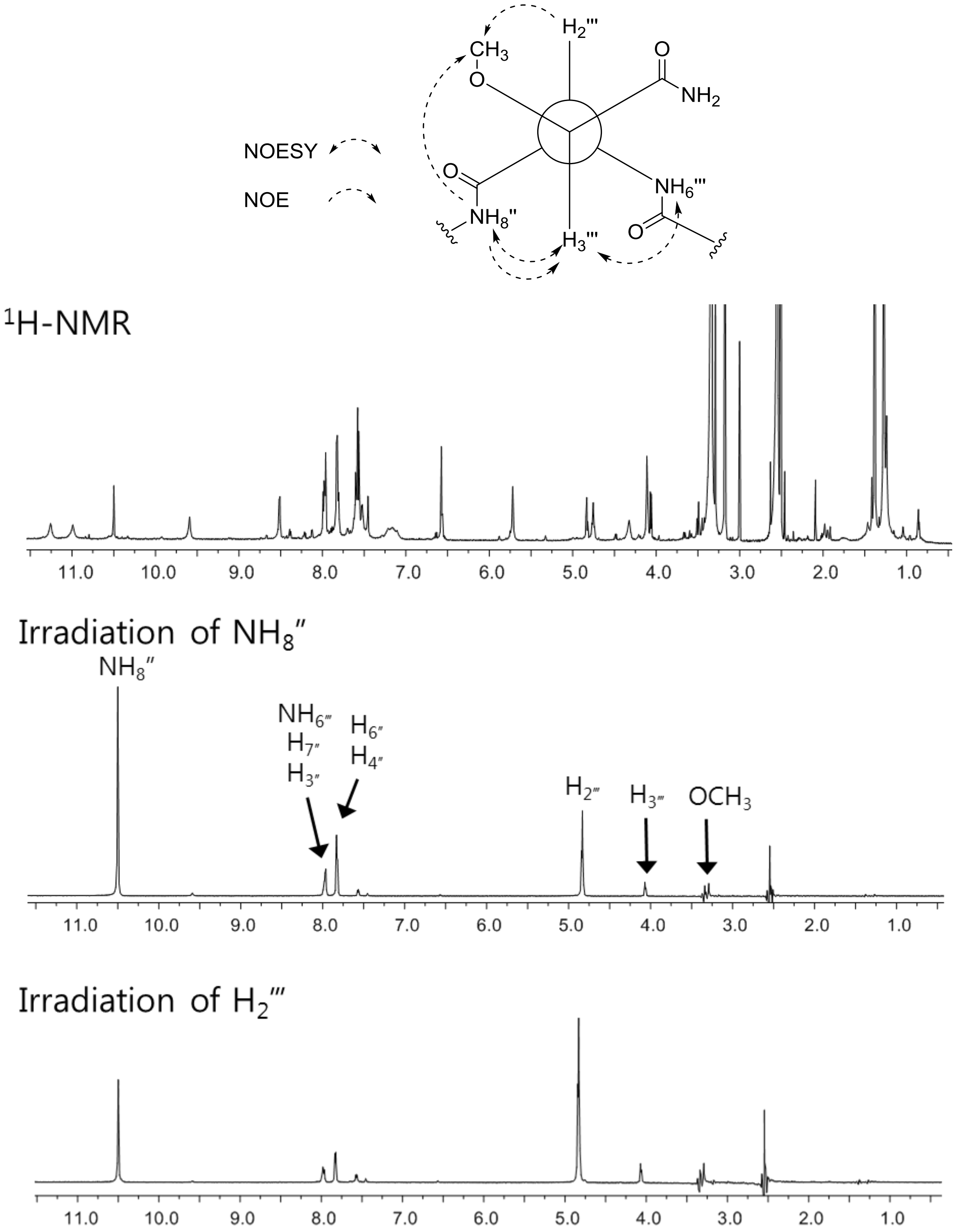

Figure S36. The NOE differential spectra of coralmycin E (**3**) measured in DMSO-d_6 at 800 MHz.

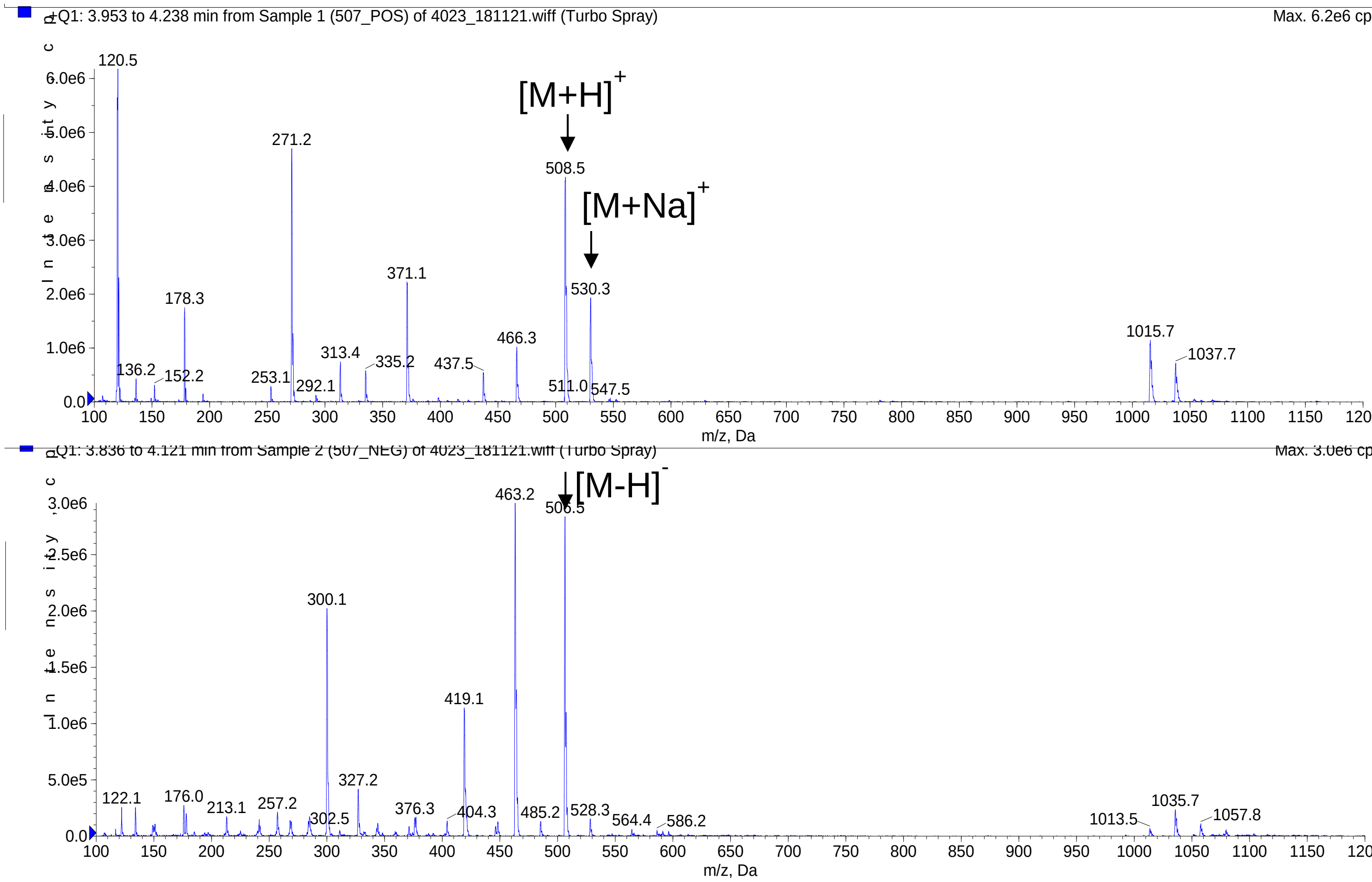

Figure S37. Positive and negative ESIMS spectrum of cystobactamid 507 (**10**).

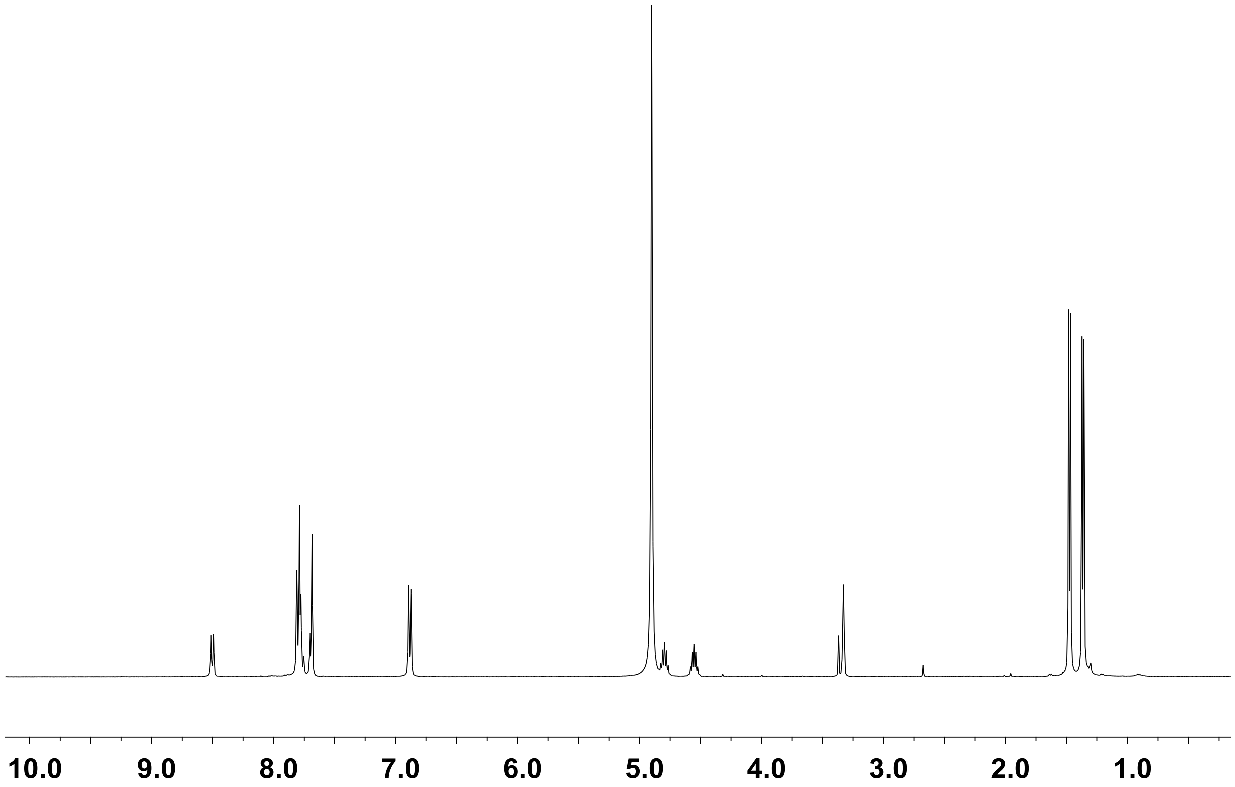

Figure S38. ^{1}H NMR spectrum of cystobactamid 507 (**10**) measured in CD_3OD at 700 MHz.

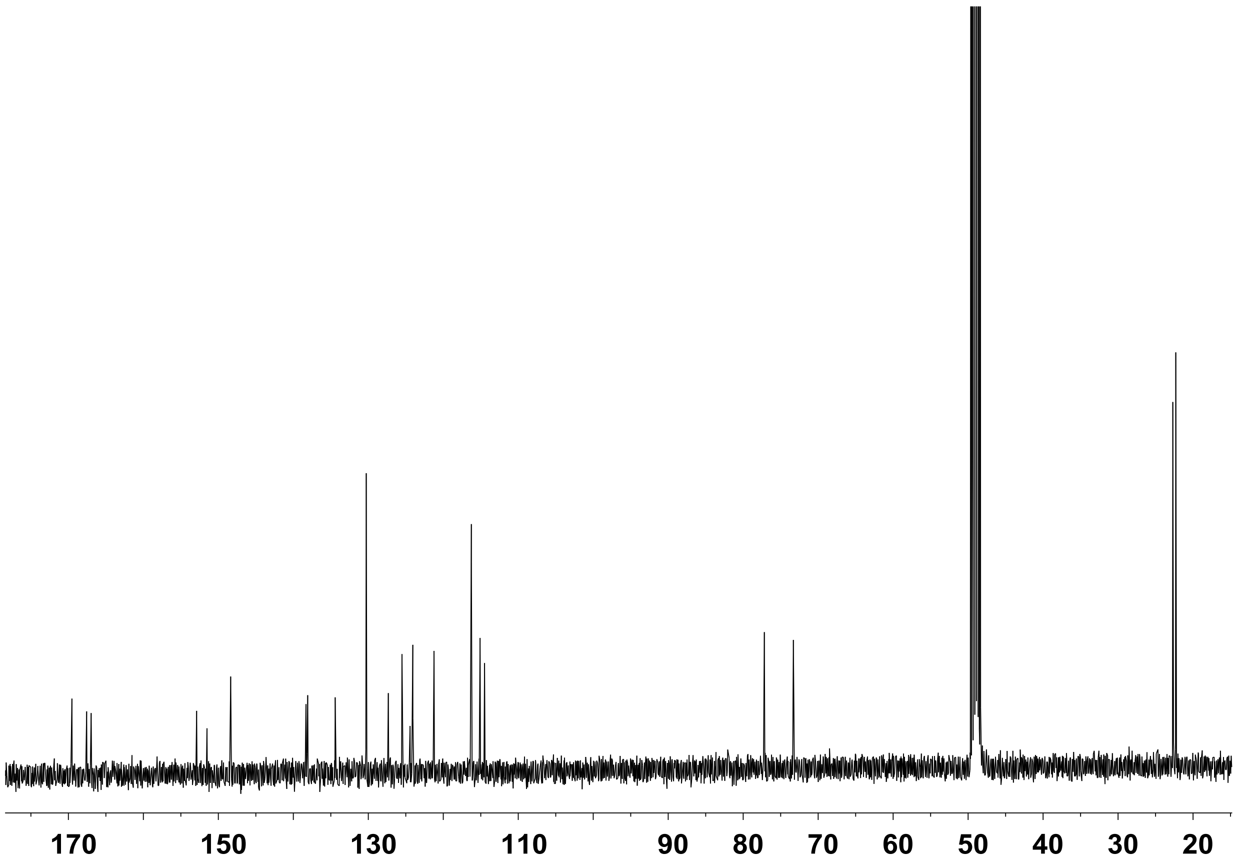

Figure S39. ^{12}C-NMR spectrum of cystobactamid 507 (**10**) measured in CD_3OD at 700 MHz.

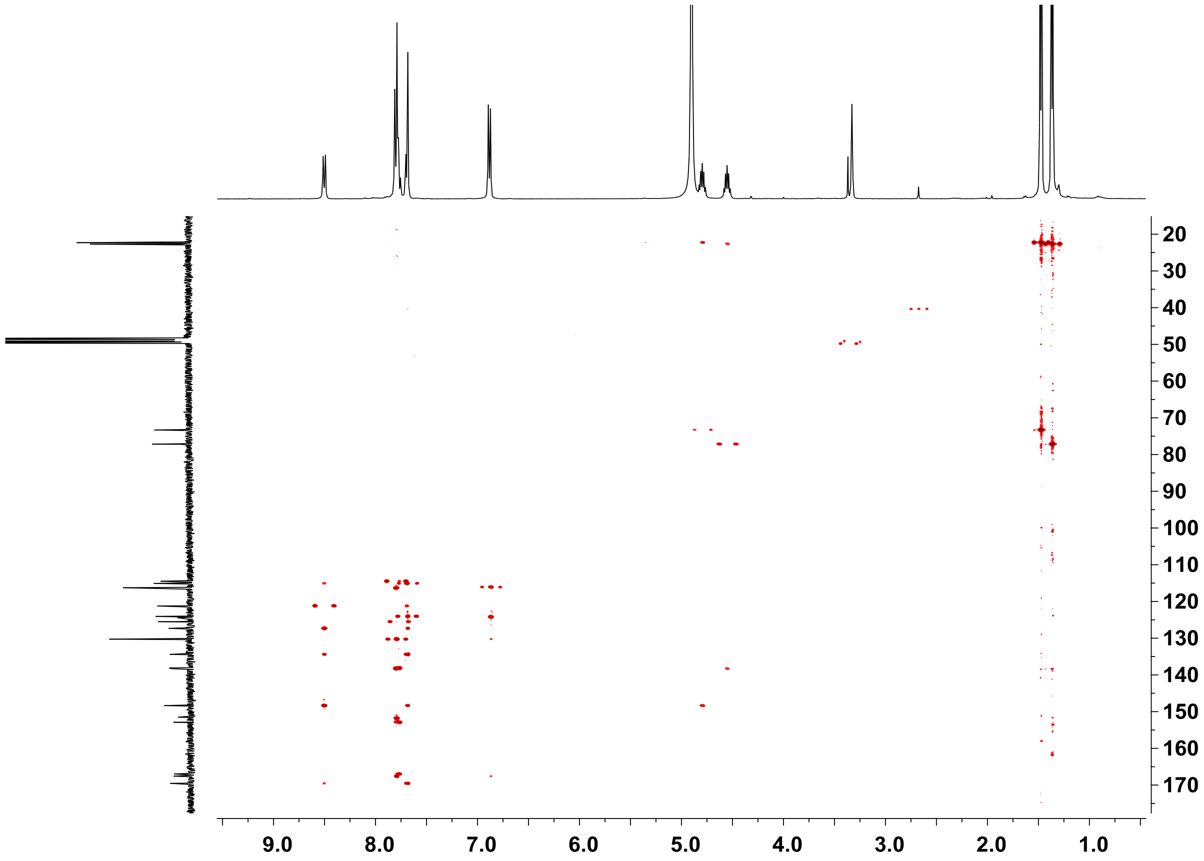

Figure S40. HMBC spectrum of cystobactamid 507 (**10**) measured in CD_3OD at 800 MHz.

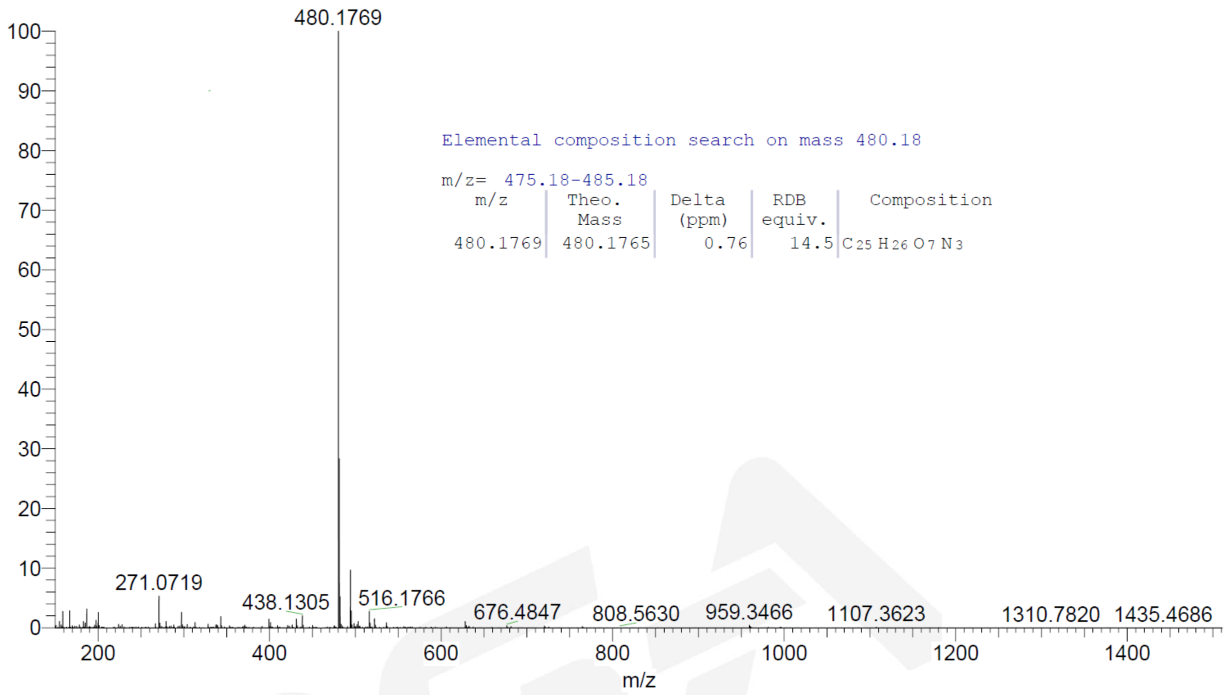

Figure S41. Positive HRESIMS spectrum of coralmycin F (**4**).

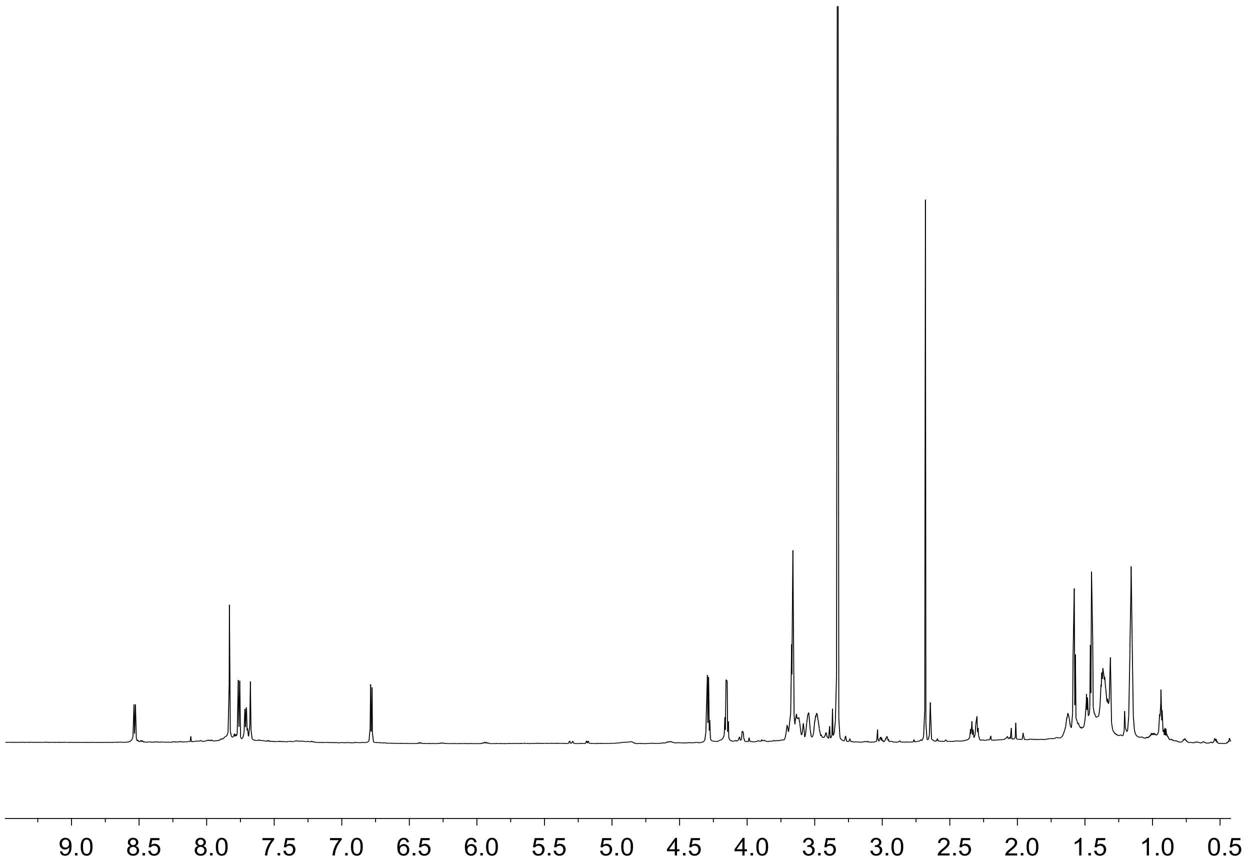

Figure S42. ^{1}H NMR spectrum of coralmycin F (**4**) measured in CD_3OD at 800 MHz.

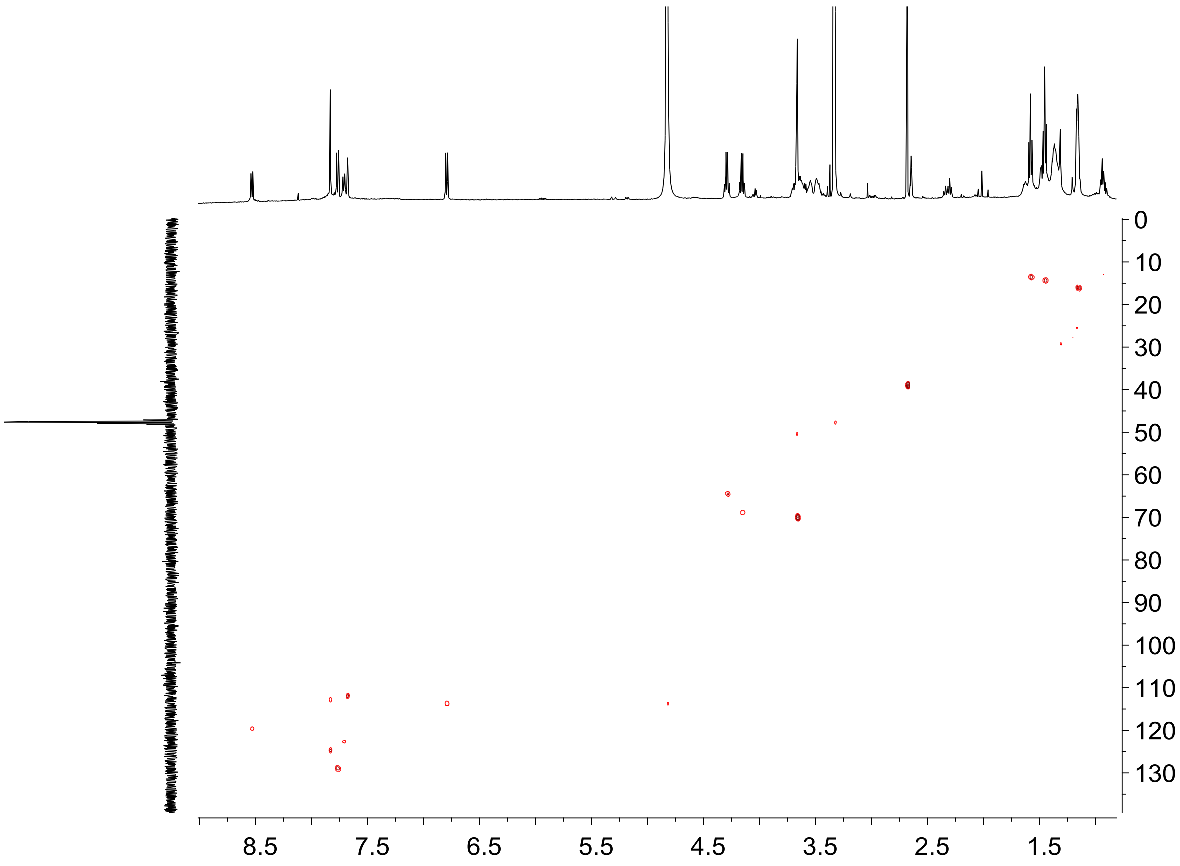

Figure S43. HMQC spectrum of coralmycin F (**4**) measured in CD_3OD at 500 MHz.

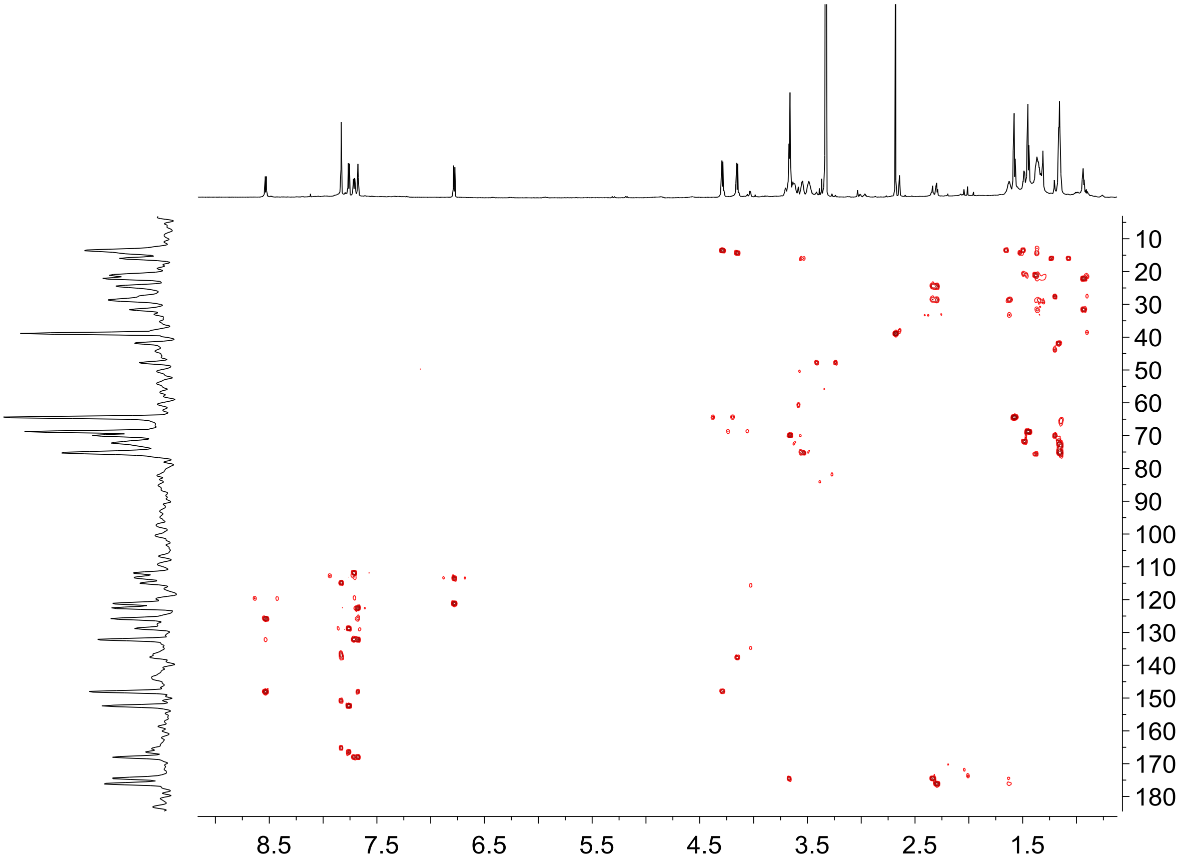

Figure S44. HMBC spectrum of coralmycin F (**4**) measured in CD_3OD at 800 MHz.

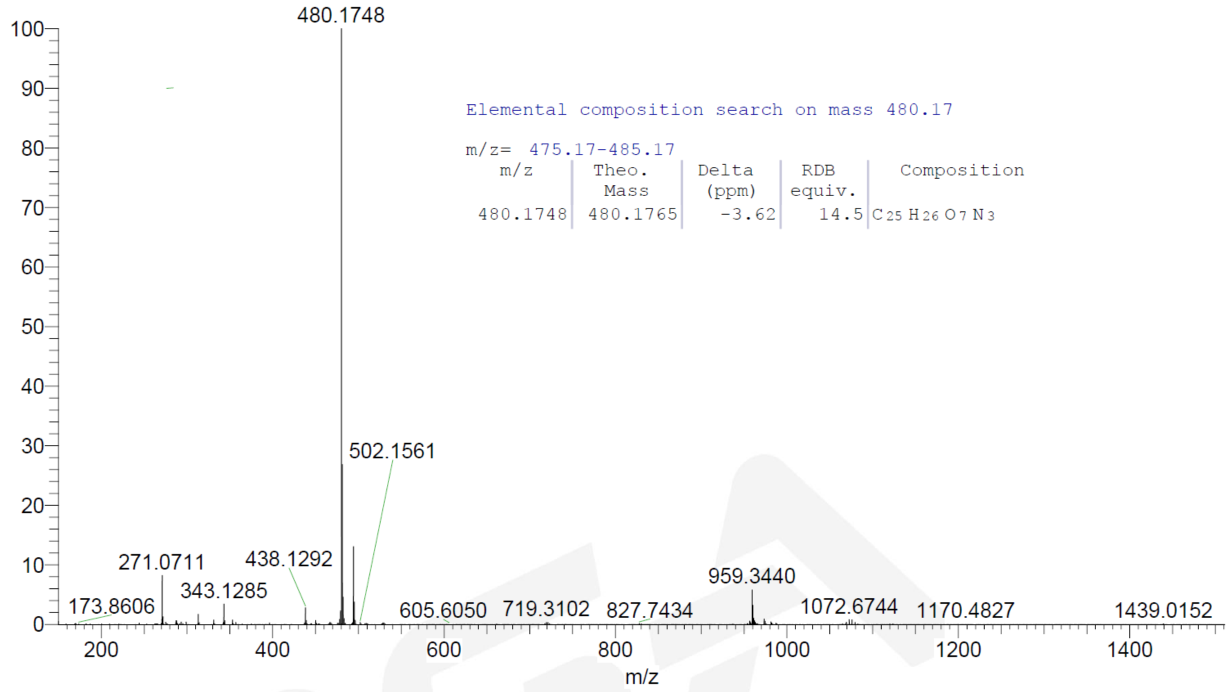

Figure S45. Positive HRESIMS spectrum of coralmycin G (**5**).

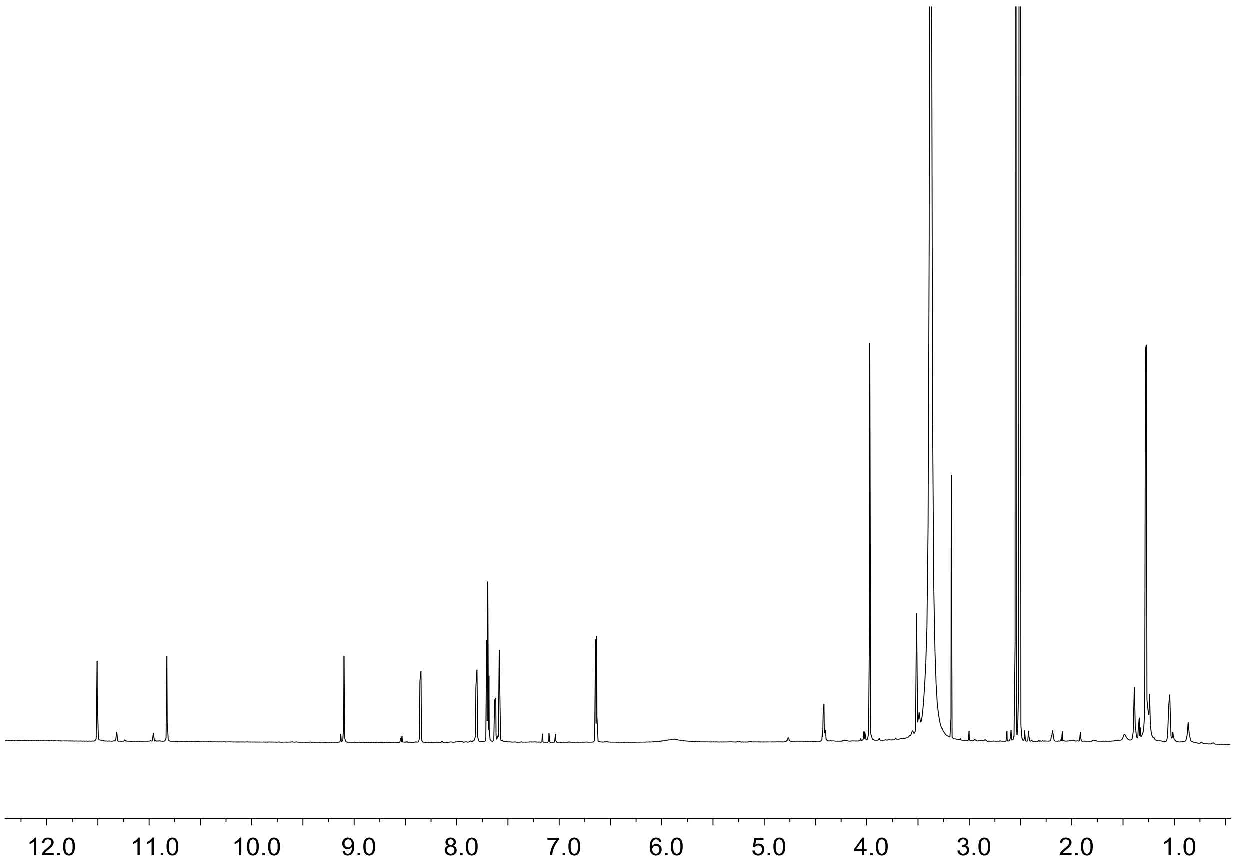

Figure S46. ^{1}H NMR spectrum of coralmycin G (**5**) measured in DMSO-d_6 at 800 MHz.

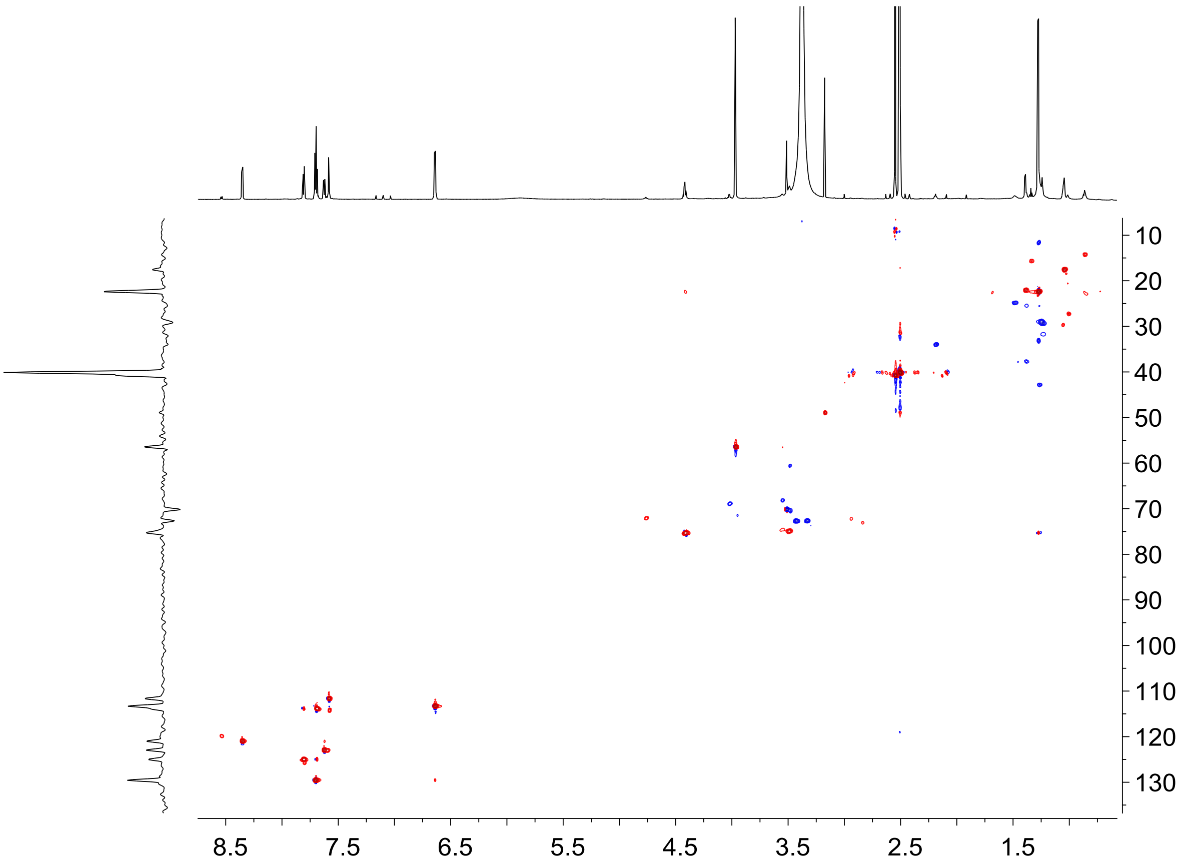

Figure S47. HMQC spectrum of coralmycin G (**5**) measured in DMSO-d_6 at 800 MHz.

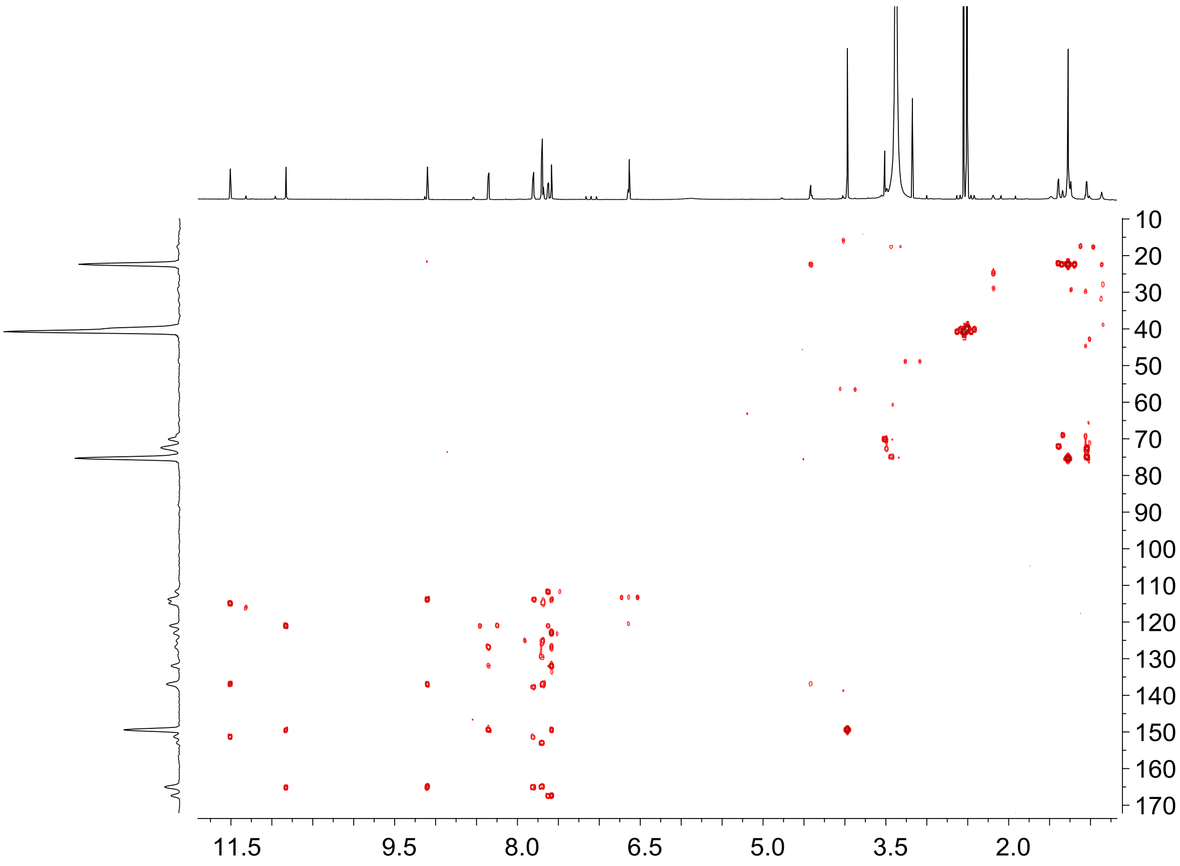

Figure S48. HMBC spectrum of coralmycin G (**5**) measured in DMSO-d_6 at 800 MHz.

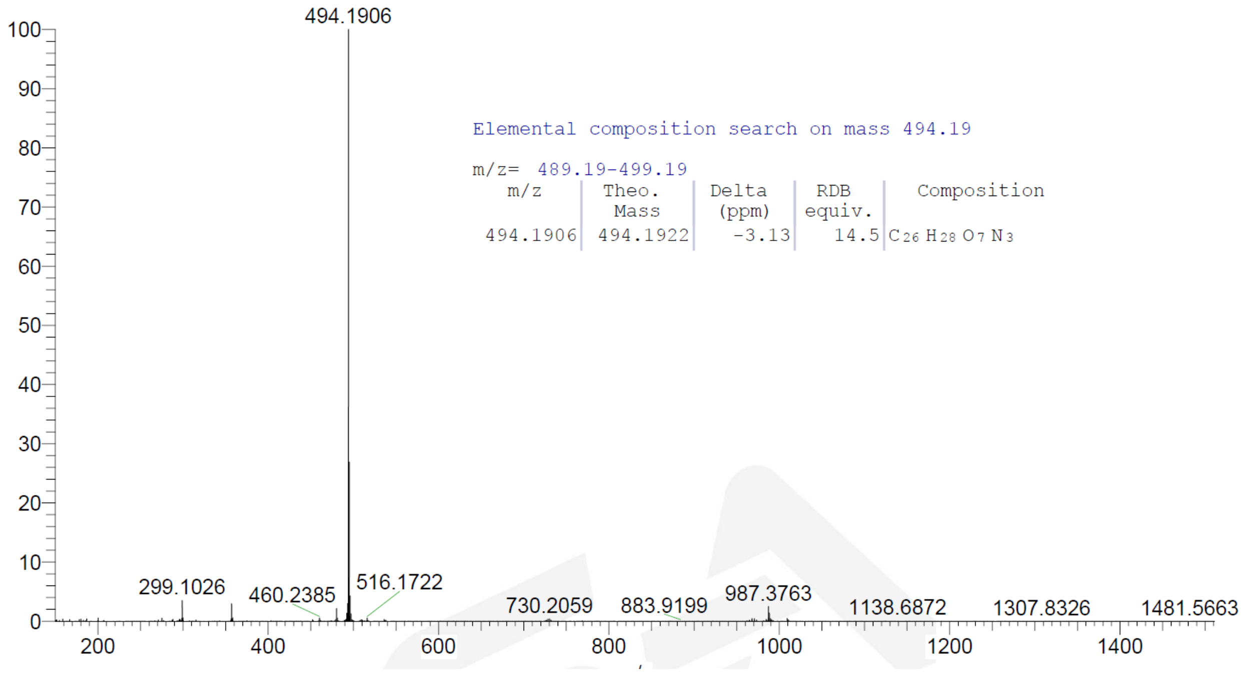

Figure S49. Positive HRESIMS spectrum of coralmycin H (**6**).

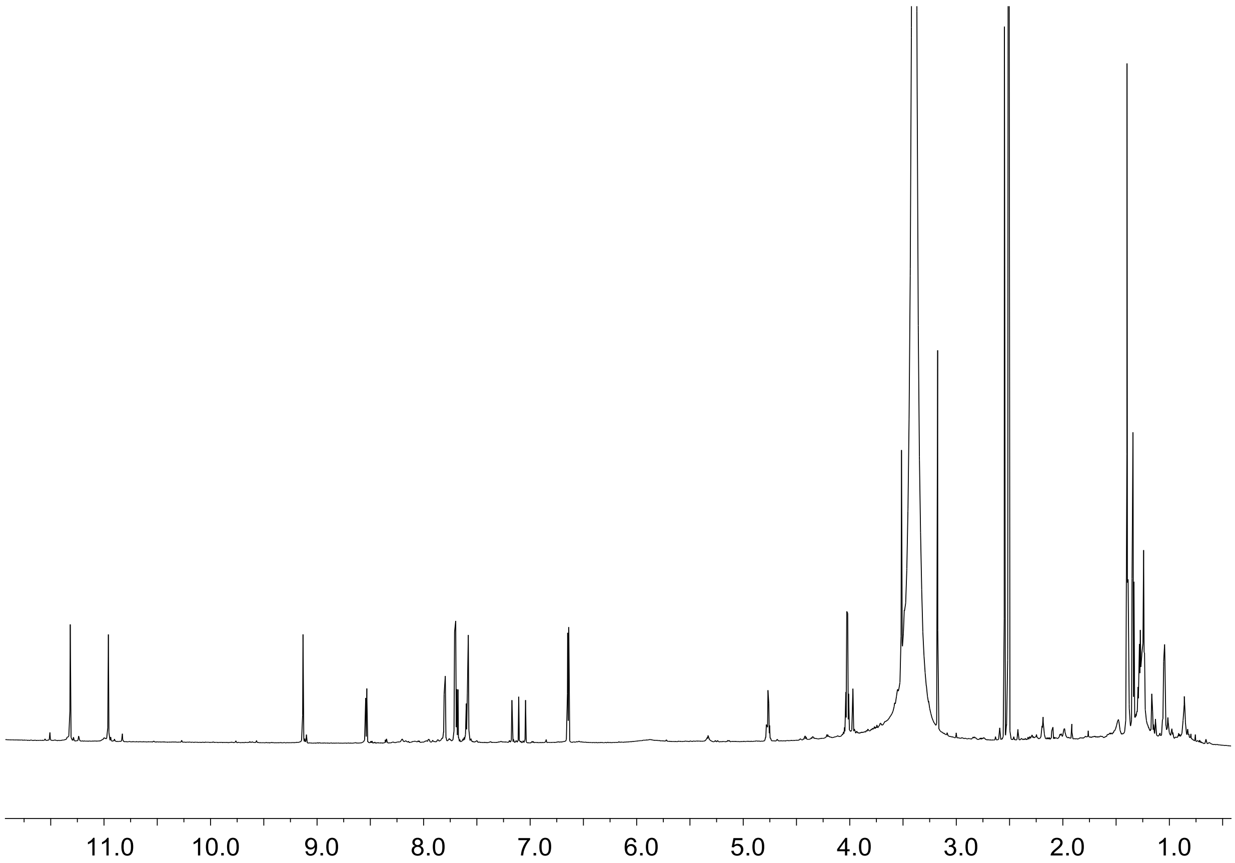

Figure S50. ^{1}H NMR spectrum of coralmycin H (**6**) measured in DMSO-d_6 at 800 MHz.

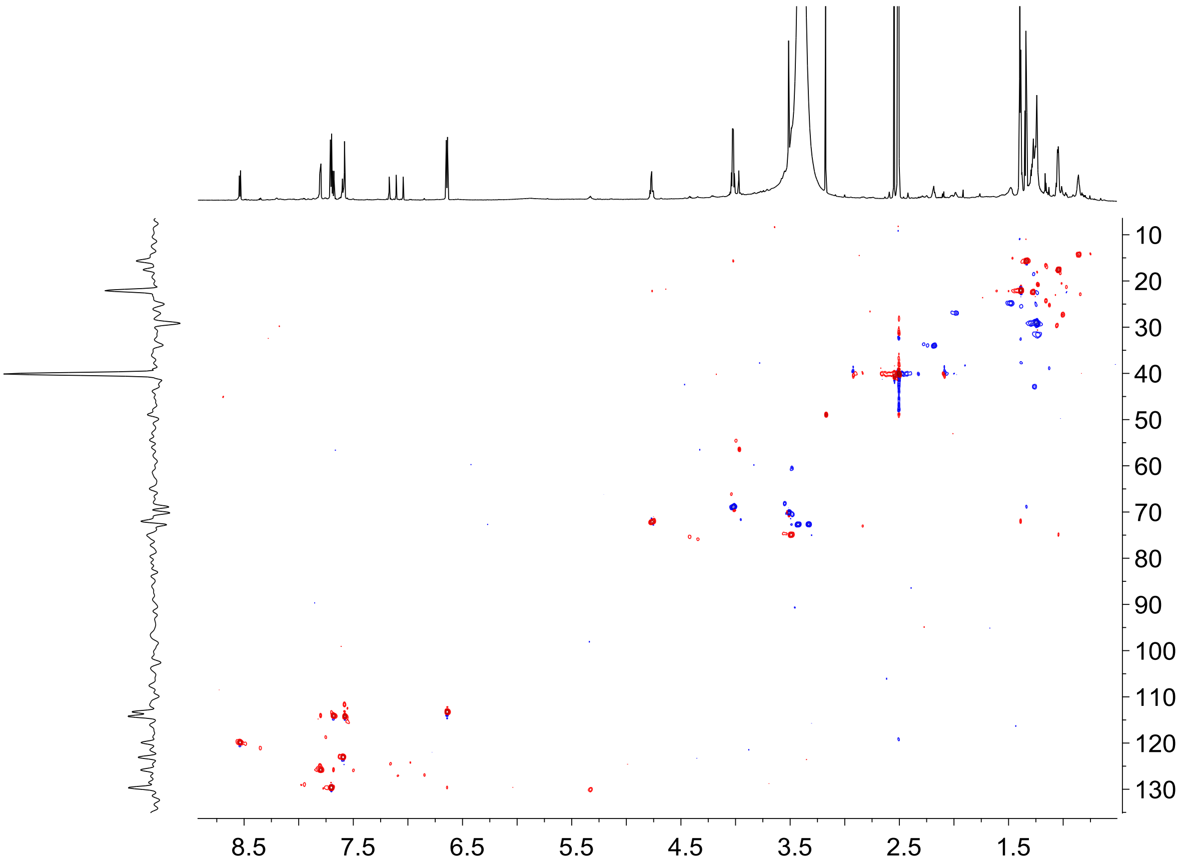

Figure S51. HMQC spectrum of coralmycin H (**6**) measured in DMSO-d_6 at 800 MHz.

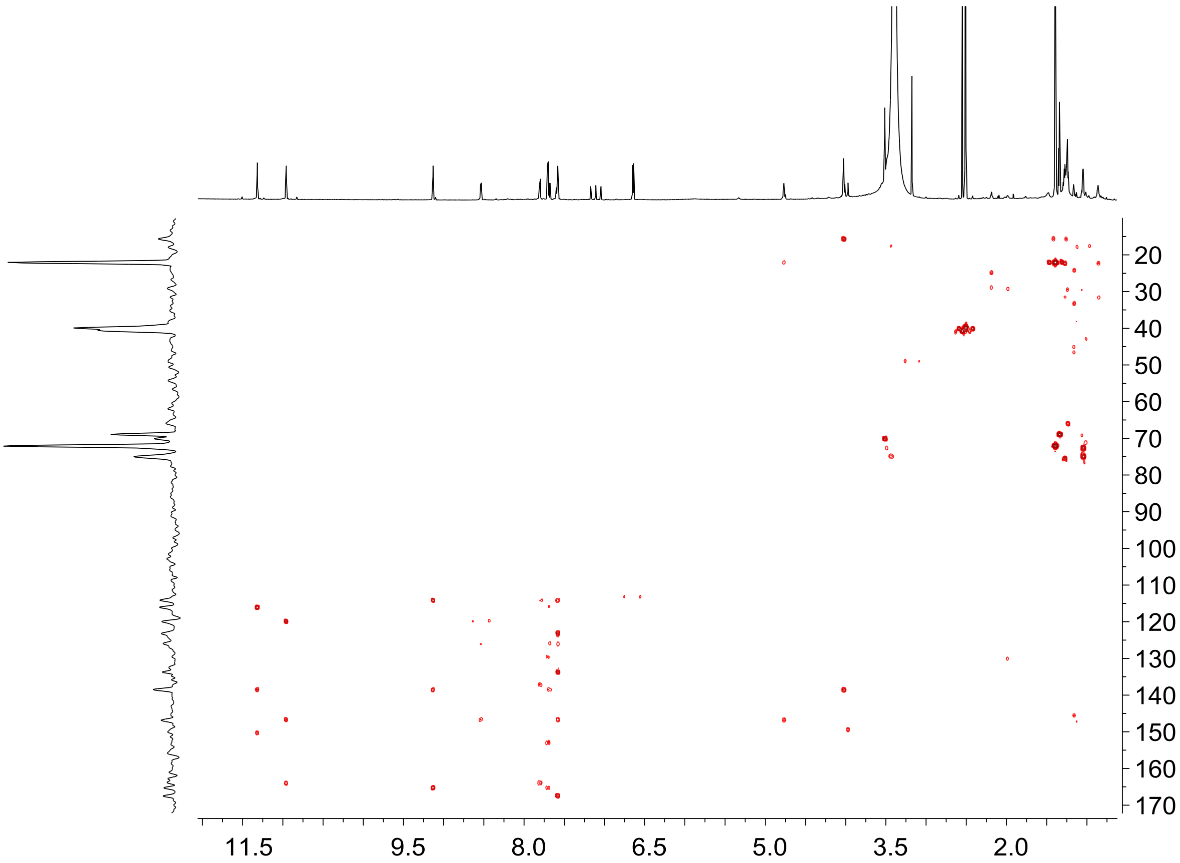

Figure S52 HMBC spectrum of coralmycin H (**6**) measured in DMSO-d_6 at 800 MHz.

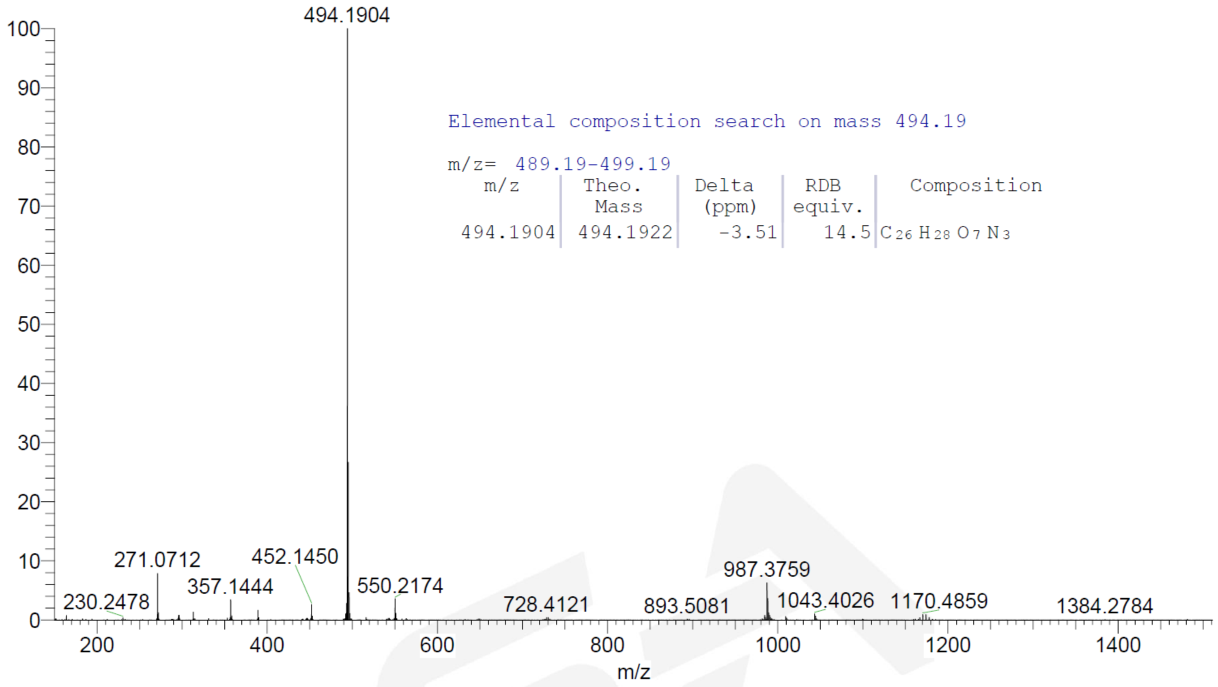

Figure S53. Positive HRESIMS spectrum of coralmycin I (**7**).

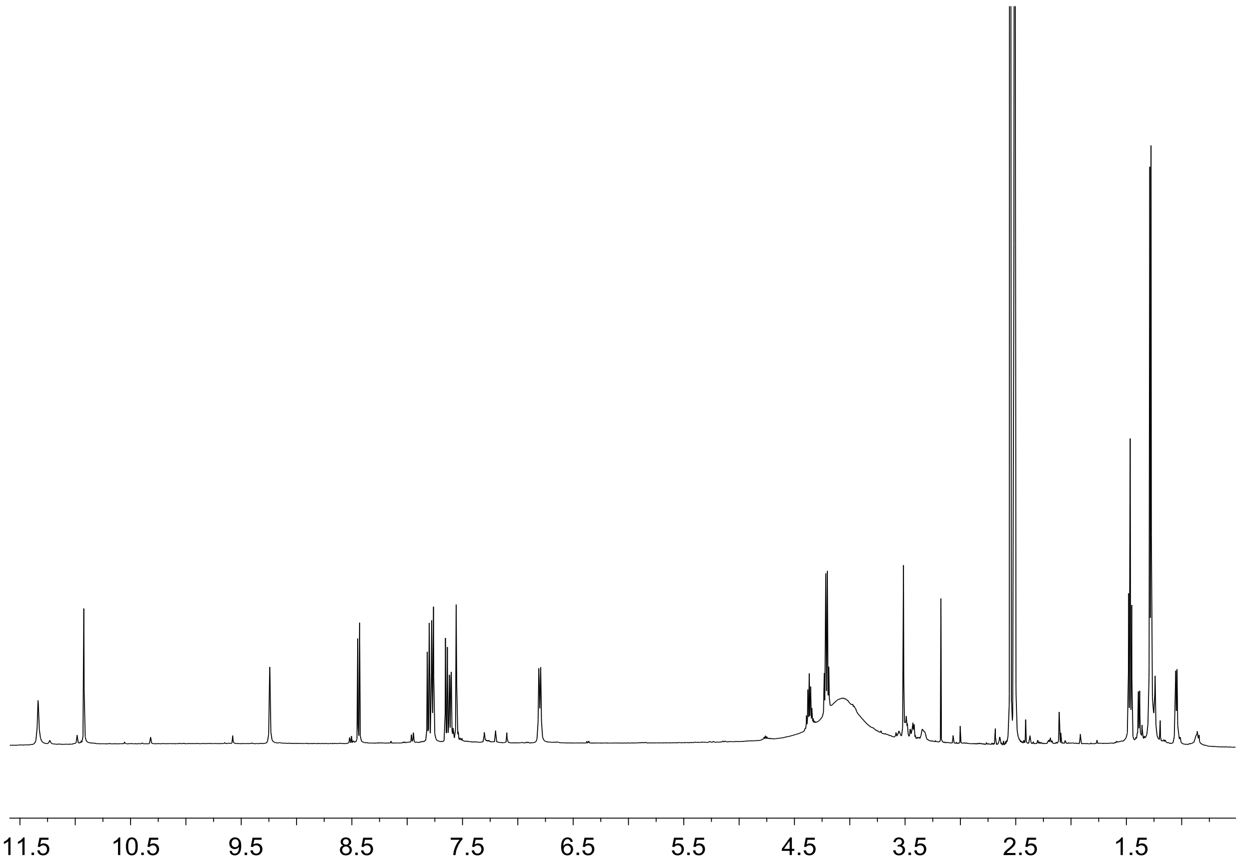

Figure S54. ^{1}H NMR spectrum of coralmycin I (**7**) measured in DMSO-d_6 at 500 MHz.

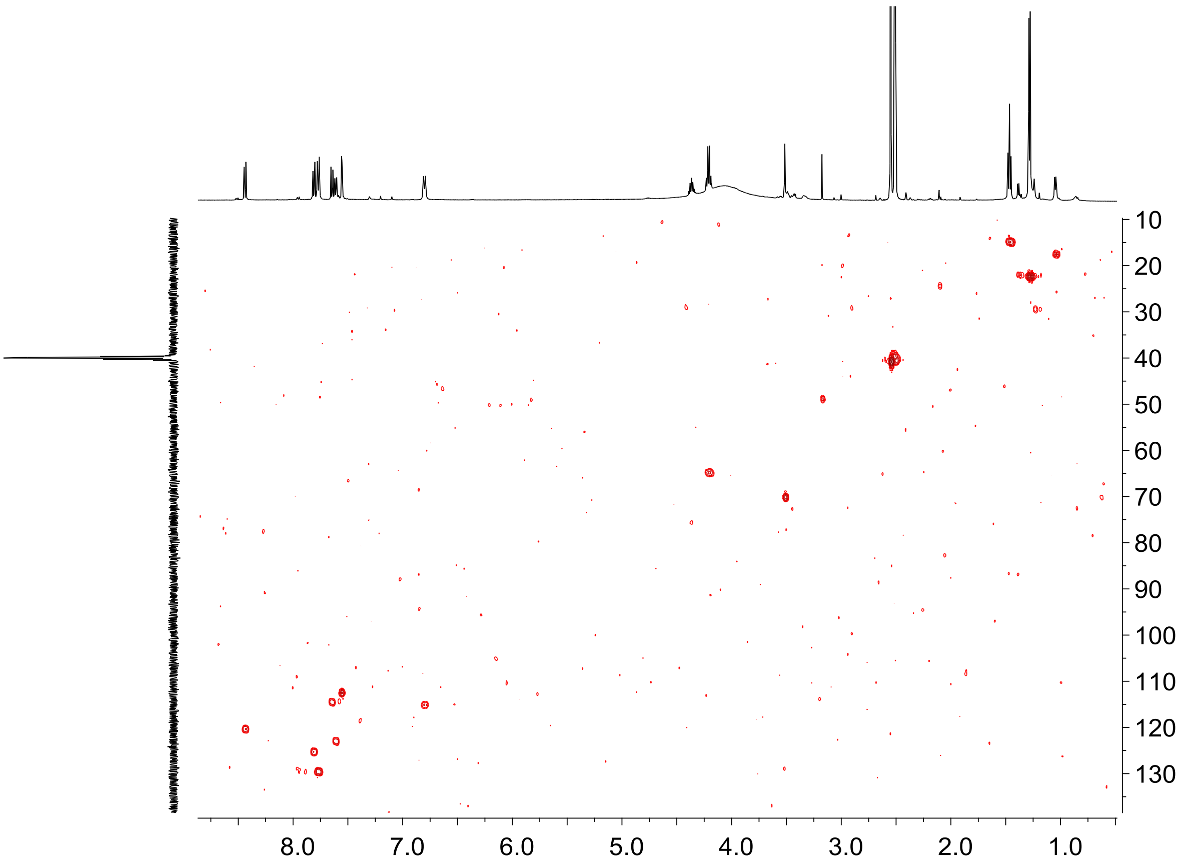

Figure S55. HMQC spectrum of coralmycin I (**7**) measured in DMSO-d_6 at 500 MHz.

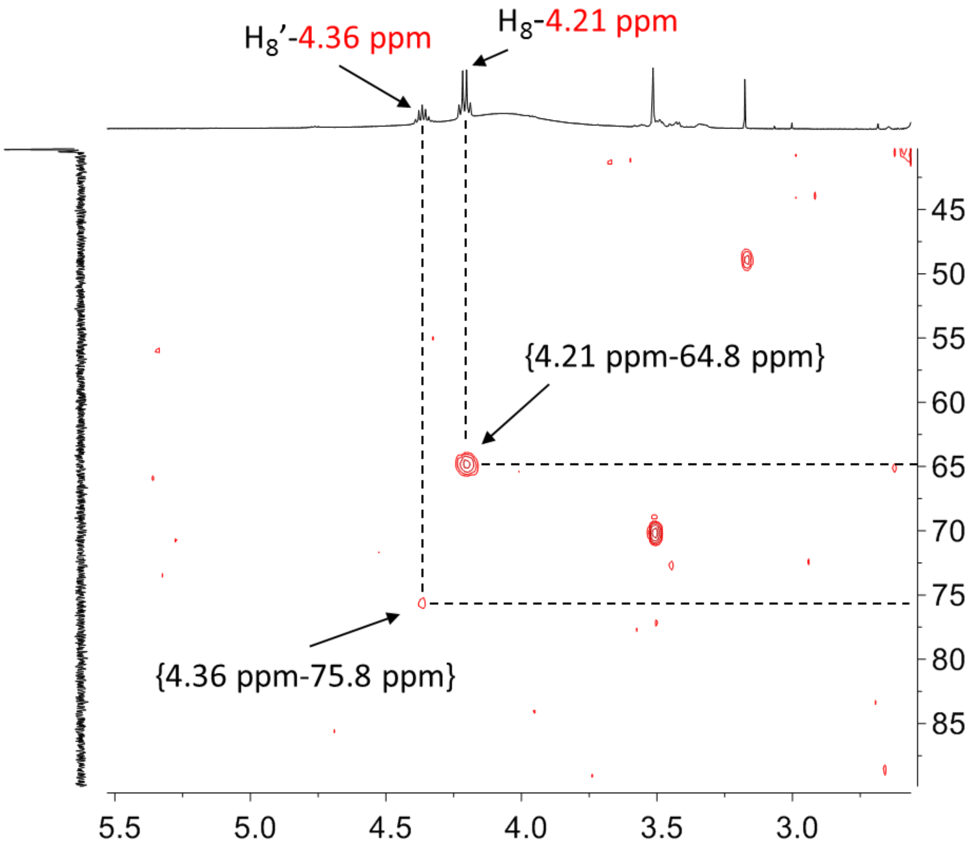

Figure S56. Expansion of HMQC spectrum of coralmycin I (**7**).

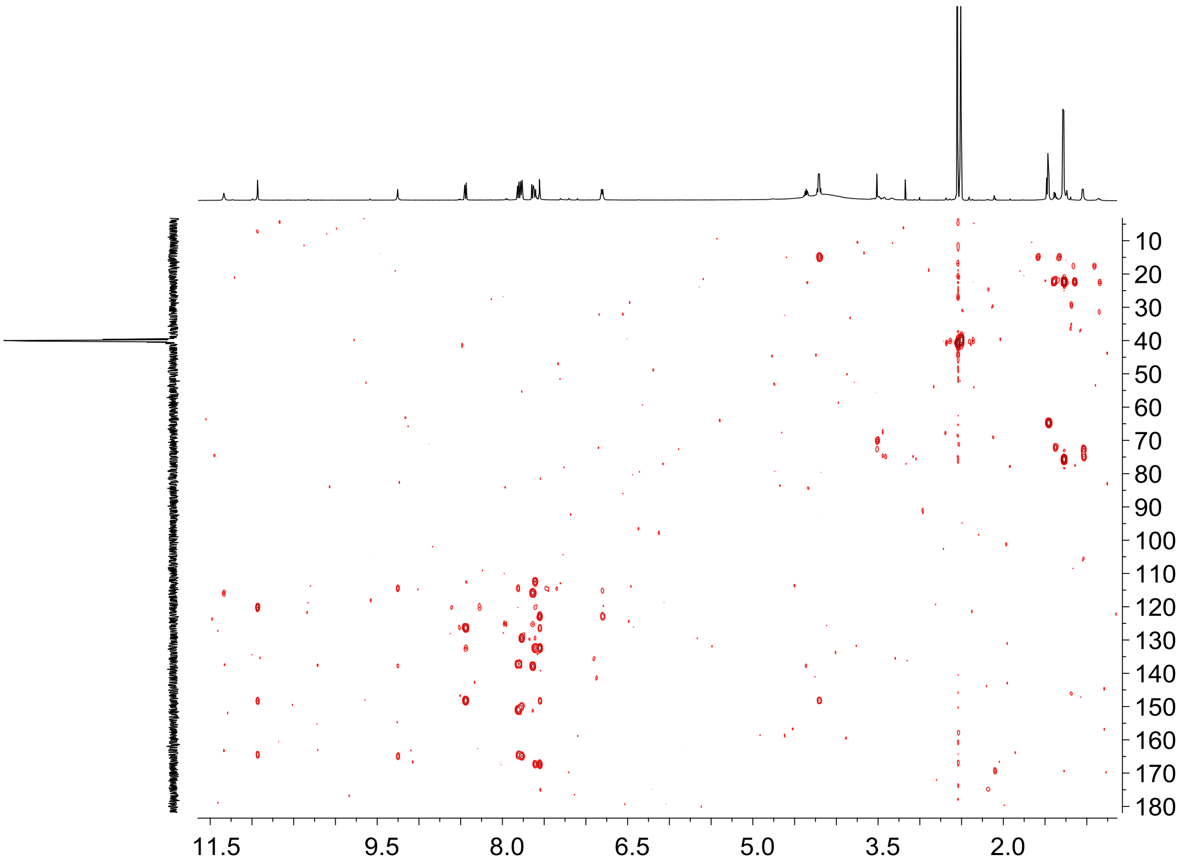

Figure S57. HMBC spectrum of coralmycin I (**7**) measured in DMSO-d_6 at 500 MHz.

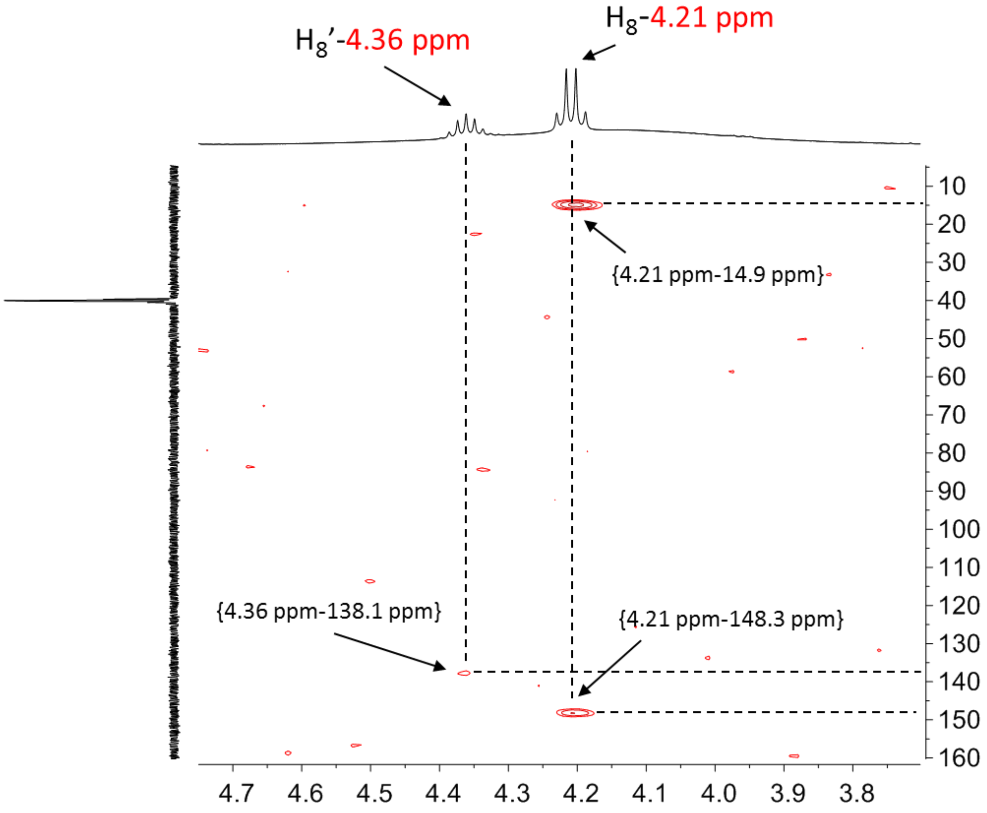

Figure S58. Expansion of HMBC spectrum of coralmycin I (**7**).

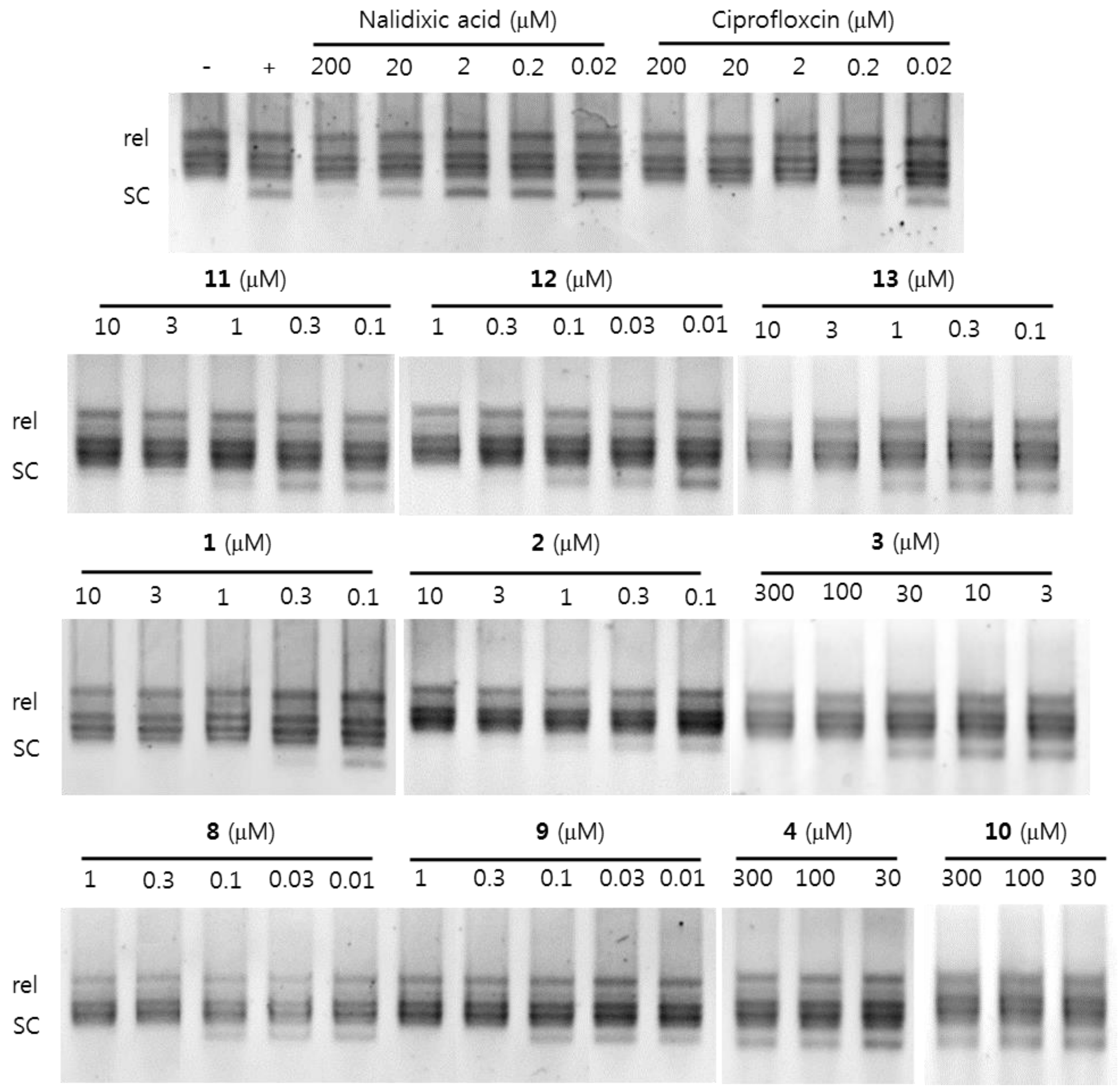

Figure S59. Agarose gels of *E. coli* gyrase supercoiling reactions inhibited by coralmycins (C (**1**), D (**2**), E (**3**), F (**4**), A (**12**), and B (**13**)) and cystobactamids (891-2 (**8**), 905-2 (**9**), 919-2 (**11**), and 507 (**10**)). (-), reaction without *E. coli* gyrase; (+), standard reaction in presence of 5% DMSO; rel, relaxed plasmid; SC, supercoiled plasmid.

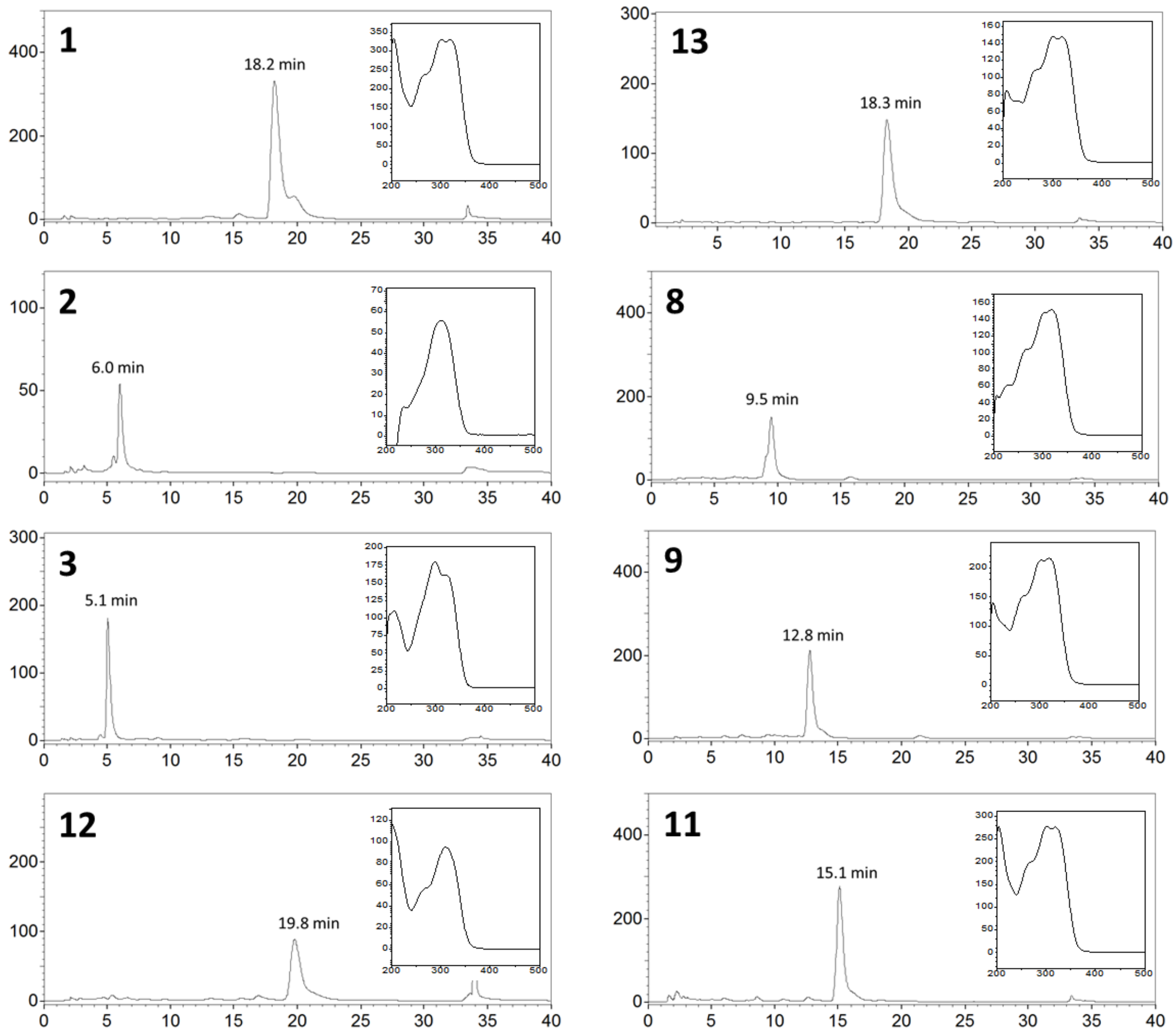

Figure S60. HPLC profiles of coralmycins (C (**1**), D (**2**), E (**3**), A (**12**), and B (**13**)) and cystobactamids (891-2 (**8**), 905-2 (**9**), and 919-2 (**11**)). J'sphere ODS-H80 (150x4.6 mm I.D), 50% ACN + 0.01% TFA, 0.8 mL/min, 25°C

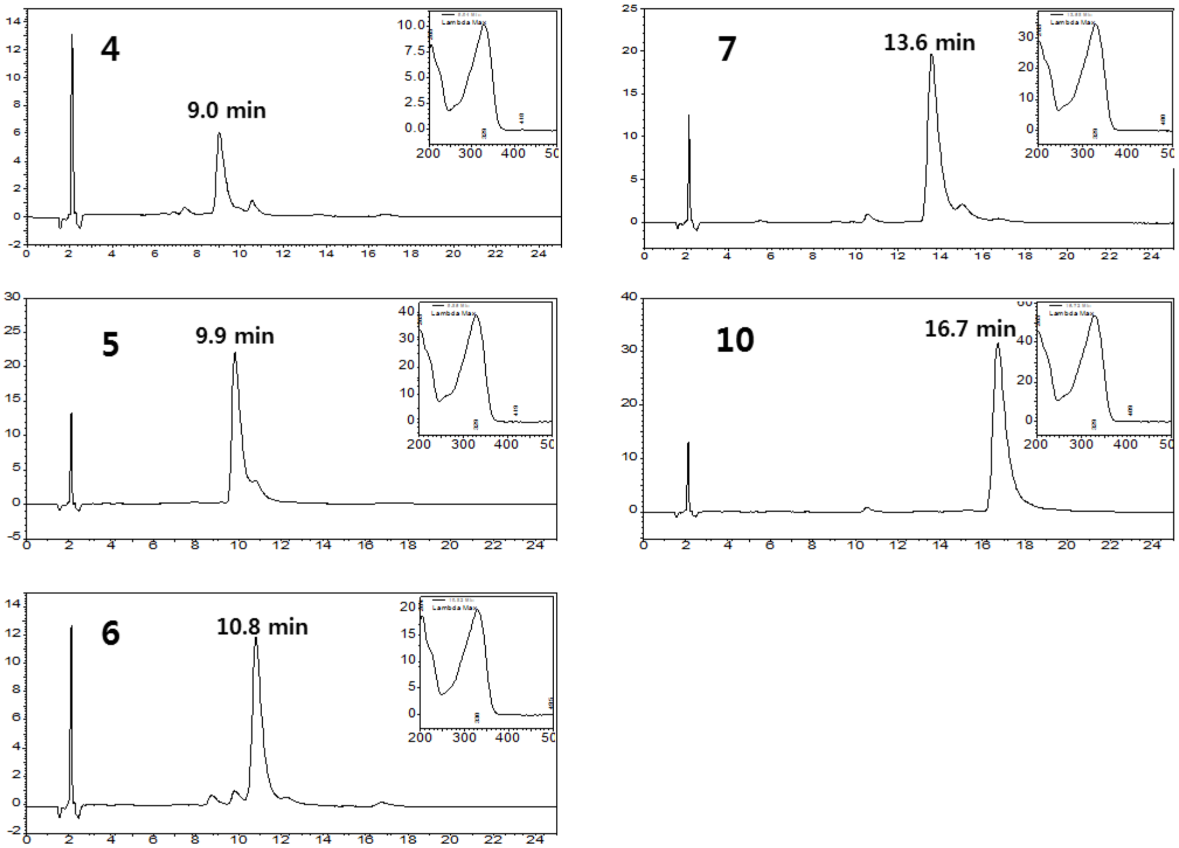

Figure S61. HPLC profiles of coralmycins F (**4**), G (**5**), H (**6**), and I (**7**), and cystobactamid 507 (**10**). J'sphere ODS-H80 (150x4.6 mm I.D), 50% ACN + 0.01% TFA, 0.8 mL/min, 25°C.
